# Optimising scale-up of injectable lenacapavir for HIV pre-exposure prophylaxis in South Africa: A modelling study and economic evaluation

**DOI:** 10.1371/journal.pmed.1004882

**Published:** 2026-07-21

**Authors:** Lise Jamieson, Leigh F. Johnson, Jeffrey W. Imai-Eaton, Hasina Subedar, Linda-Gail Bekker, Gesine Meyer-Rath

**Affiliations:** 1 Health Economics and Epidemiology Research Office, School of Clinical Medicine, Faculty of Health Sciences, University of the Witwatersrand, Johannesburg, South Africa; 2 South African Centre for Epidemiological Modelling and Analysis (SACEMA), Centre for Epidemic Response and Innovation (CERI), School for Data Science and Computational Thinking, Stellenbosch University, Stellenbosch, South Africa; 3 Centre for Integrated Data and Epidemiological Research, School of Public Health, University of Cape Town, Cape Town, South Africa; 4 Center for Communicable Disease Dynamics, Department of Epidemiology, Harvard TH Chan School of Public Health, Boston, Massachusetts, United States of America; 5 MRC Centre for Global Infectious Disease Analysis, School of Public Health, Imperial College London, London, United Kingdom; 6 National Department of Health, Pretoria, South Africa; 7 Desmond Tutu HIV Centre, University of Cape Town, Cape Town, South Africa; 8 Department of Global Health, Boston University School of Public Health, Boston, Massachusetts, United States of America; Institut Pasteur, FRANCE

## Abstract

**Background:**

South Africa accounts for 20% of global HIV infections. Six-monthly injectable lenacapavir (LEN) for HIV pre-exposure prophylaxis (PrEP) has superior efficacy to oral tenofovir disoproxil fumarate/emtricitabine (TDF/FTC), and similar efficacy to 2-monthly injectable cabotegravir (CAB). With LEN’s regulatory approval, South Africa faces critical implementation decisions amid constrained domestic resources and reduced international funding. We evaluated the epidemiological impact, cost-effectiveness, and optimal populations for LEN roll-out in South Africa.

**Methods and findings:**

Using Thembisa v4.8, a deterministic compartmental HIV transmission model, we simulated the impact of LEN scale-up, expanded oral TDF/FTC, and CAB scale-up, compared to a baseline of current TDF/FTC provision over 20 years (2026–2045) in South Africa. We modelled PrEP among women, including adolescent girls and young women (AGYW), female sex workers (FSW), pregnant and breastfeeding women (PBFW), men who have sex with men (MSM), and heterosexual men. For TDF/FTC scale-up, we doubled baseline initiation rates. For LEN and CAB, conservative and optimistic scenarios assumed initiation rates similar to or double those under TDF/FTC scale-up, respectively. Duration of use varied by subpopulation under TDF/FTC (3–6 months), conservative (LEN: 6–12 months, CAB: 4–8 months), and optimistic (LEN: 12–24 months, CAB: 8–16 months) scenarios. We modelled strategies to maximise impact of ~500,000 LEN doses allocated for 2026–2027, and national roll-out by subpopulations. We compared LEN cost-effectiveness to other HIV interventions, including antiretroviral treatment (ART). Costs are presented from the South African government’s perspective in undiscounted 2025 United States Dollars (USD).

Providing LEN to 1.7–2.9 million South Africans annually averted 19%–31% of infections and saved 3%–5% of life years lost to HIV, reaching incidence <0.1% in 2039–2043, 10–14 years earlier than baseline. TDF/FTC and conservative LEN scale-up increased HIV programme costs by 3%; however, LEN was more cost-effective, costing $2,301–$3,567/life year saved (LYS) versus $8,143/LYS (TDF/FTC scale-up) and $11,114–$16,118/LYS (CAB). While promising for HIV prevention, scaling up condoms, ART, HIV testing, and medical male circumcision may be more cost-effective than LEN; notably ART at 95% coverage, saved 4.8-8-fold more life years compared to LEN, costing $577/LYS. Prioritising AGYW, MSM, and FSW for the initial allocation maximised infections averted, while national roll-out prioritising FSW and MSM were most cost-effective, and even cost-saving under scale-up to FSW. Study limitations include uncertainty in achieving modelled uptake, risk-differentiated uptake of long-acting products, and real-world implementation costs.

**Conclusions:**

Delivering LEN to persons with elevated HIV risk in South Africa is more cost-effective than existing oral PrEP and can speed up HIV incidence reduction. Prioritising uptake among groups at highest risk is essential to maximise impact and cost-effectiveness.

## Author summary

**Why was this study done?:**

- Lenacapavir has shown remarkable efficacy in preventing HIV acquisition in clinical trials and recently became available in South Africa for early implementation, backed by the Global Fund.
- Recent price negotiations with generic manufacturers have resulted in an agreed price for South Africa of $40 per person per year and $17 for the loading dose.
- South Africa has the world’s largest HIV epidemic but faces severe funding constraints, making it critical to understand whether lenacapavir represents good value for money and which populations should be prioritised for roll-out.

**What did the researchers do and find?:**

- We used a mathematical model of South Africa’s HIV epidemic to simulate the epidemiological impact and costs of rolling out lenacapavir compared to existing oral HIV prevention pills, or another injectable option, cabotegravir, over 20 years.
- We compared lenacapavir to existing HIV interventions, including antiretroviral treatment (ART), condoms, HIV testing, and male circumcision, to assess its value for money in the broader South African HIV response, and found that while lenacapavir outperforms other PrEP options, these existing interventions remain more cost-effective overall; expanding ART substantially reduced both HIV infections and life years lost to AIDS.
- Lenacapavir was more effective and better value for money than existing oral prevention pills, particularly when targeted at female sex workers and men who have sex with men.
- For the initial allocation of ~500,000 person-years of lenacapavir, funded by the Global Fund, prioritising adolescent girls and young women alongside key populations maximised the number of infections prevented.

**What do these findings mean?:**

- Lenacapavir could meaningfully accelerate the reduction of HIV infections in South Africa, but prioritising people at the highest risk is essential to maximise its impact and cost-effectiveness.
- Under funding constraints, maintaining and expanding access to ART offers greater value for money over new prevention scale-up, as it saves substantially more life years at lower cost.
- Our findings are based on modelled rather than real-world data, and actual impact will depend on how many people initiate and stay on lenacapavir, the true costs of delivering injectable drugs at scale, and how well services can reach the highest-risk individuals across different regions of the country.

## Introduction

South Africa is home to the largest population with HIV, with an estimated 8 million people living with HIV (PLHIV), and, despite a 53% reduction in new infections since 2010, remains the country with the second highest rate of HIV acquisition worldwide [1,2]. Lenacapavir (LEN), a 6-monthly injectable antiretroviral drug, for HIV as pre-exposure prophylaxis (PrEP) has demonstrated superior efficacy to oral PrEP with tenofovir diphosphate/emtricitabine (TDF/FTC) in the PURPOSE-1 AND PURPOSE-2 randomised clinical trials, both of which were unblinded early due to the extremely high efficacy of LEN [3,4]. LEN is promising for HIV prevention, particularly in South Africa, which leads the world in oral TDF/FTC uptake, accounting for 20% (roughly ~600,000) of annual PrEP initiations.

LEN was recently approved by the South African Health Products Regulatory Authority for use as PrEP [5]. To support rapid initial LEN implementation in South Africa, the Global Fund has reallocated $29 million of existing funding to provide ~500,000 person-years on LEN in 2026 and 2027 at a subsidised drug price to the South African HIV programme of $60 per person per year (PPPY) [6]. Beyond this, affordable access could increase through generic manufacturing: six generic manufacturers have received voluntary licenses from the originator company to produce product for selected low- and middle-income markets, including South Africa, and two have committed to an introductory price of $40 PPPY (and $17 for an oral loading dose at initiation) on expectation of sufficient market volume, a similar annual commodity price to oral TDF/TFC [7]. Production cost estimates suggest the eventual generic manufacturing price could be further lowered to around $25 PPPY for a market demand of 5–10 million people globally [8,9].

However, strategic decisions for and the success of LEN implementation will depend on several factors, including the pace of manufacturing, supply chain logistics, and the creation of sufficient demand among and effective delivery models for those populations at highest risk of acquiring HIV infection who stand to benefit the most [10]. Additionally, the readiness of public-sector facilities, including provider training, digital system adaptations, and capacity to manage injectable PrEP at scale, will directly affect achievable uptake. South Africa largely funds its HIV programme from domestic resources, making considerations of cost-effectiveness and affordability paramount. LEN’s $40 PPPY generic price and long-acting dosing may make it not only a cost-effective option within the existing PrEP landscape, but potentially a competitive investment compared to other HIV interventions, some of which may require substantially higher costs or coverage to achieve comparable impact.

Recent studies modelling the potential impact of LEN have shown that it could substantially reduce HIV incidence in sub-Saharan Africa. An analysis covering Eastern and Southern Africa—including South Africa, Zimbabwe, and western Kenya—projected that LEN could avert 12%−18% of infections, over 10 years, at 1.6%−4.0% coverage, with higher coverage (3.2%–8.1%) resulting in higher impacts (21%−33% of infections averted) [11]. In South Africa, a nonprioritised roll-out strategy, providing 25 million person years of PrEP over a 35 years, was estimated to avert 4.4% of infections, while a risk-prioritised approach, which allocated to female sex workers (FSW) first, averted three to five times more infections [12]. Both studies generated threshold estimates for a LEN price that would render it cost-effective under a cost-effectiveness threshold of US$500 per disability-adjusted life-year. Results varied by epidemic setting according to underlying incidence, risk prioritisation, and coverage achieved: Kaftan and colleagues estimated for South Africa a price range between $23–$106 PPPY and Wu and colleagues estimated $177–$213 PPPY [11,12], with both studies estimating substantially lower pricing for Zimbabwe and Western Kenya overall.

To inform the prioritisation of the planned roll-out of LEN in the South Africa, we conducted three economic evaluations: (1) we estimated the cost and cost-effectiveness of a large-scale roll-out of LEN at the generic price of $40 PPPY, compared to CAB and oral TDF/FTC scale-up, and produced budget estimates for the South African government; (2) we compared both LEN and CAB roll-out to scaling up other existing HIV interventions, including testing, prevention and treatment; and (3) we modelled several roll-out strategies to different subpopulations to optimise the epidemiologic impact of the initial 2-year LEN allocation (~500,000 person-years annually in 2026 and 2027) (Phase 1) and the planned large-scale roll-out from 2028 onwards (Phase 2).

## Methods

### Epidemiological model

We used Thembisa (version 4.8), a deterministic compartmental HIV transmission model of the South African HIV epidemic [13]. This modelling framework is particularly appropriate for a mature, large-scale epidemic, as it captures population-level dynamics while representing HIV acquisition, disease progression, and mortality. The compartmental structure allows integration of prevention and treatment interventions across heterogeneous risk groups, providing a transparent and reproducible basis for evaluating epidemiological impact and cost-effectiveness under different implementation scenarios. Thembisa 4.8 is fitted using a Bayesian approach in which prior distributions are specified for the parameters governing sexual behaviour, HIV transmission and disease progression, and then calibrated against several data sources for HIV prevalence (antenatal clinic data, national household surveys, FSW and MSM prevalence studies) as well as data on recorded deaths, antiretroviral metabolites and age distributions for adult ART patients. The model population is stratified by age, sex, sexual experience, sexual behaviour, marital status, HIV testing history, and male circumcision status. The sexually experienced population is divided into two broad sexual risk groups: ‘high-risk’ (people who engage in concurrent partnerships and/or commercial sex) and ‘low-risk’; FSWs are modelled as a subset of high-risk unmarried women, and men who have sex with men (MSM) are modelled as subsets of the unmarried low-risk and high-risk groups. PrEP uptake rates vary by age, sex, and sexual risk group. Regarding sexual risk, the model assumes that PrEP uptake is proportional to the product of the individual’s annual number of partners and the HIV prevalence in their partners, resulting in higher uptake of PrEP of higher risk individuals. The model assumes that PrEP users (all modalities have a 10% lower rate of condom use than individuals of the same age, sex, and risk who are not using PrEP). Oral TDF/FTC effectiveness incorporates both efficacy and adherence, and was assumed to be 85% for MSM, and 65% for FSW, other women and heterosexual men [14–16]. LEN effectiveness was assumed to be 99% for all populations modelled, based on data from the PURPOSE trials [3,4]. CAB effectiveness was assumed to be 95% across all populations [17,18]. For both LEN and CAB, we assumed that a pharmacokinetic tail protection is present beyond the respective 6- and 2-month protection that a single injection provides. For the main analysis, we assumed the duration of this tail protection to be 6 months (LEN) and 3 months (CAB), based on available pharmacokinetic studies; we varied these in sensitivity analysis [4,19]. Additionally, the model captures variability in tail duration using an exponential distribution, so effectiveness declines gradually after a missed dose rather than dropping abruptly, reflecting pharmacokinetic evidence of reduced tail-phase protection [19]. The model assumed that the relative rates of uptake between the subpopulations modelled remain similar with injectable PrEP to that of oral TDF/FTC. To ensure comparability, all PrEP options were modelled within the same harmonised Thembisa framework using identical population structure, risk stratification, and transmission dynamics, with behavioural response to PrEP applied uniformly across all modalities. Differences in effectiveness, duration of use, visit schedules, and tail protection reflect evidence-based product characteristics, derived from South African programme data for oral TDF/FTC and from clinical trial data for injectable PrEP. More details on the epidemiological model structure, and details of model calibration and sources for assumptions, are available online at Thembisa.org [13].

### Scenarios and assumptions

For the first analysis, we modelled LEN, CAB, and TDF/FTC scale-up over a 20-year time horizon, starting from 2026, with uptake in all women, particularly adolescent girls and young women (AGYW) aged 15–24 years, pregnant and breastfeeding women (PBFW), FSW, MSM, and heterosexual men. These options capture South Africa’s full current and emerging biomedical prevention landscape, helping decision-makers evaluate whether transitioning to newer long-acting formulations provides sufficient value-for-money compared to established regimens. The baseline scenario was the present HIV programme with the current TDF/FTC roll-out, amounting to a coverage of 7% for AGYW, 0.1% (PBFW), 11% (FSW), 10% (MSM), and 0.2% (heterosexual men) (Table 1). For TDF/FTC and conservative LEN or CAB scale-up, we assumed initiation rates doubled compared to baseline, while under our optimistic LEN or CAB scale-up scenarios, we doubled these rates again. These scenarios imply a similar or higher preference for long-acting products compared to TDF/FTC, a decision that can be justified by preference studies conducted in the local or regional context [20,21]. PBFW has low rates of PrEP uptake at baseline, but we assumed 29% coverage under TDF/FTC, 16% under LEN, and 21%−22% under CAB. Under LEN and CAB scenarios, we assumed continuation of the current baseline rates of TDF/FTC initiation remains unchanged. We assumed an average duration of use for TDF/FTC of 3 months for all women and heterosexual men, and 6 months for MSM. Duration of use for LEN under the conservative scenario was assumed to be 6 months (women, heterosexual men) and 12 months (MSM), and under the optimistic scenario this doubled with 12 months (women, heterosexual men) and 24 months (MSM). Under CAB the duration assumed was less, with MSM having the longer assumed duration (conservative: 4–8 months; optimistic: 8–16 months). We evaluated the impact on life years lost due to AIDS, new HIV infections, and incremental cost-effectiveness for each of these outcomes. These assumptions resulted in varying coverage levels across scenarios and subpopulations. Results presented are deterministic point estimates and key assumptions are detailed in Table 1.

**Table 1:** Key assumptions* on uptake, duration, and effectiveness for baseline and PrEP scale-up scenarios.

	Scenarios						
**Key assumption**	**Baseline**	**TDF/FTC**	**LEN conservative**	**LEN optimistic**	**CAB conservative**	**CAB optimistic**	**Source**
**Effectiveness in preventing HIV infection**	85% (MSM) 65% (non-MSM)		99% (all populations)		95% (all populations)		[3,4,14–18]
**Uptake rates of PrEP**	Based on current TDF/FTC programme data [13]; no LEN or CAB rolled out	Doubled uptake rates of TDF/FTC	LEN uptake rates doubled baseline TDF/FTC; TDF/FTC uptake rates remain at baseline	LEN uptake rates doubled conservative LEN scale-up; TDF/FTC uptake rates remain at baseline	CAB uptake rates doubled baseline TDF/FTC; TDF/FTC uptake rates remain at baseline	CAB uptake rates doubled conservative CAB scale-up; TDF/FTC uptake rates remain at baseline	[13] *for baseline initiation rates, relative uptake between populations*
**Duration of use**	6 months (MSM) 3 months (non-MSM)		12 months (MSM) 6 months (non-MSM)	24 months (MSM) 12 months (non-MSM)	8 months (MSM) 4 months (non-MSM)	16 months (MSM) 8m months (non-MSM)	
**Tail protection**	0 months		6 months		3 months		[4,19]
Cost of provision of TDF/FTC per person initiated**	$57 (non-MSM), $72 (MSM)		As baseline		As baseline		[26,35]
Cost of provision of LEN or CAB per person initiated**	Not applicable		$60–65 (non-MSM), $85 (MSM)	$86–91 (non-MSM), $137 (MSM)	$146–147 (non-MSM), $226 (MSM)	$227–228 (non-MSM), $388 (MSM)	[26,35]

*A more detailed version of this table depicting modelled coverage levels by subpopulation is presented in Table A in S1 Appendix. Abbreviations: CAB = cabotegravir; LEN = lenacapavir; PrEP = pre-exposure prophylaxis; TDF/FTC = tenofovir/emtricitabine; USD = United States Dollars. **Full service cost presented includes costs for staff, HIV testing, laboratory testing, drugs, consumables and overheads. Assumed price for LEN (drug only) = $40 PPPY (4 injections/year) and $17 for a loading dose (1,200 mg LEN tablets); price for CAB (drug only) = $30/injection or ~$180–210 per person per year (6–7 injections/year)

For the second analysis, we used Thembisa to model the impact and cost per life-year saved of PrEP scale-up to expansion of other existing HIV interventions, similar to the previous analysis for the annual South African HIV Investment Case [22,23]. We constructed a simple league table by ranking interventions based on their incremental cost-effectiveness ratio (ICER) per LYS. Interventions were scaled up separately, with each compared to baseline: ART (95% coverage, an increase from the current 81% coverage in 2025 among PLHIV who know their status), condom distribution (from 418 million to 725 million condoms/year), medical male circumcision (MMC) (from ~210,000/year to ~500,000/year), general HIV testing (from 16.5 million to 18 million), an HIV self-screening package consisting of distributing HIV self-test kits mostly to partners of ART patients (from 333,000/year to 1 million/year) [24], polymerase chain reaction (PCR) testing of infants at 10 weeks (from 80% to 95% coverage), PCR testing of infants at 6 months (from 40% to 95% coverage), and rapid HIV testing of infants at 18 months (from 30% to 95% coverage).

The third analysis modelled LEN strategies maximise the epidemiologic impact of (i) the current Global Fund LEN allocation (covering ~500,000 person-years over 2026–2027) (Phase 1) [6], and (ii) the large-scale roll-out (Phase 2). The National Department of Health (NDOH) had indicated that the planned priority populations for Phase 1 were AGYW, PBFW, FSW, MSM, and transgender individuals, reaching up to 500,000 initiations over 2026–2027. We modelled the impact of Phase 1 with the objective to maximise new HIV infections averted over a 5-year time horizon (2026–2030), reflecting primary and short-term secondary infections averted. The binding constraint was having 500,000-person years of LEN for 2026–2027, and no LEN from 2028 onwards as to isolate the impact of Phase 1 alone. For simplicity, we assumed all populations had an average duration on LEN for 1 year. We modelled 490 combinations of discrete increments of LEN initiations across subpopulations, subject to pre-defined feasibility constraints: maximum coverages of 60% for FSW (45,000 initiations/year) and 30% for MSM (93,500 initiations/year), reflecting realistic upper bounds on reachable key population size (Table B in S1 Appendix). We constrained the annual initiations to 250,000, implying a maximum number of initiations each of 250,000 for women, of which most were AGYW and PBFW, corresponding to population coverages of 4% and 20%, respectively. Transgender individuals were not included as Thembisa does not currently model this population dynamically.

For Phase 2, we modelled a large-scale LEN roll-out delivered to each subpopulation separately, as well as selected combinations of mostly AGYW, PBFW, FSW, and MSM, over a 20-year time horizon (2026–2045), and compared the impact and cost to baseline. Our uptake assumptions were intentionally conservative, aligning with baseline TDF/FTC initiation numbers, and somewhat higher for key populations (FSW, MSM) to explore the potential impact under more optimistic uptake scenarios. We assumed conservative average durations: 6 months for women and heterosexual men, and 12 months for MSM. Overall, this analysis resulted in a number of initiations reaching AGYW: ~700,000/year, FSW: ~100,000/year, PBFW: ~400,000/year, MSM: ~150,000/year and heterosexual men: ~600,000/year.

### Cost analysis

Costs were analysed from the perspective of the South African government and reported in 2025 United States Dollar (USD; exchange rate 18.22 South African Rand (ZAR) per 1 USD) [25]. Costs are undiscounted, representing data use for budgeting purposes. The cost of PrEP provision was estimated using an ingredients-based approach; the full methodology has been described elsewhere [26]. Briefly, PrEP is provided in primary healthcare clinics and includes HIV testing, counselling, provision of condoms, syndromic management of sexually transmitted infections with treatment referral, training, outreach, mobilisation, monitoring, and evaluation costs. Visit schedules varied according to each product (normalised to a 12-month duration; LEN: 2 per year; CAB: 7 per year; oral TDF/FTC: 5 per year). The cost of LEN was assumed to be $40 PPPY for the injections and $17 for the loading dose, as per the recently announced agreement with generic manufacturers and funders [27]. The cost of CAB was $180 PPPY ($30 per injection) [28], while oral TDF/FTC was $41 PPPY ($3.38 for one month’s supply) at current generic prices. The full-service cost of PrEP provision for all scenarios is summarised in Table 1. The estimation of costs of the other HIV interventions, as well as the HIV programme overall followed the same approach as the South African HIV Investment Case [22,29]. We estimated cost-effectiveness over a 20-year time horizon (2026–2045) as the incremental cost per HIV infection averted and incremental cost per LYS, compared to baseline.

### Sensitivity analysis

We conducted a one-way sensitivity analysis within our main analysis to evaluate the impact of key PrEP parameters, including uptake, differential risk targeting across PrEP options, TDF/FTC duration, LEN/CAB tail protection, and costs. We also conducted sensitivity analysis to examine whether improved or poorer risk targeting, or higher service delivery costs for FSW or MSM, affected the impact of different subpopulation distributions in Phase 1, or the relative cost-effectiveness of LEN scale-up in Phase 2 of our third analysis. Furthermore, a probabilistic sensitivity analysis was conducted to evaluate the impact of the uncertainty of 58 key parameters in Thembisa on the main cost-effectiveness analysis [13], including PrEP-specific parameters such as PrEP effectiveness, duration on TDF/FTC, tail protection for injectable PrEP, and reduction in condom use while on PrEP. We varied the cost of LEN service delivery (excluding drug costs) with lower and upper bounds varying by 50% from our average estimate. A Monte Carlo simulation, encompassing 1,000 iterations, sampled data from pre-determined distributions associated with PrEP-related key parameters as described in Table C in S1 Appendix. We report median estimates across cost and health outcomes with 95% uncertainty bounds (UB) estimated using 2.5th and 97.5th percentiles.

This study is reported as per the Consolidated Health Economic Evaluation Reporting Standards (CHEERS 2022) Statement (S1 Checklist).

### Ethics

As this study did not include primary human subjects data, no ethical clearance was sought.

### Use of artificial intelligence tools and technologies

During the preparation of this manuscript, the authors utilised Microsoft Co-Pilot (GPT-4) exclusively to refine grammar and improve phrasing fluidity. No artificial intelligence tools were used to generate original scientific concepts, or perform modelling simulations or analyses. The authors critically evaluated and verified all language refinements to ensure scientific accuracy and contextual integrity. The authors retain full responsibility for the final content and integrity of the completed manuscript.

## Results

### Epidemiological impact

**Fig 1:**
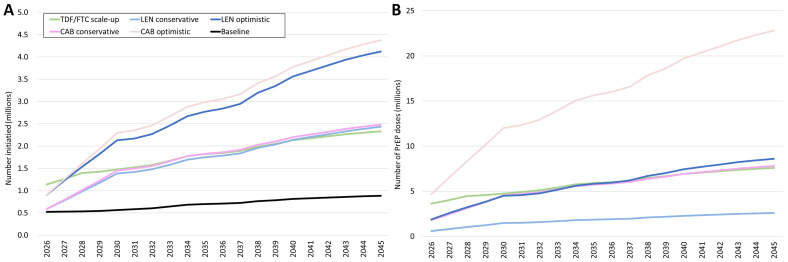
Number of (A) PrEP initiations and (B) doses by PrEP type per year for large-scale roll-out. Dosing frequency differs by PrEP type: TDF/FTC is represented by monthly packs; CAB requires an injection at initiation, month 1, and then 2-monthly; LEN requires an injection 6-monthly, loading dose for every initiation.

The numbers initiating PrEP increased substantially in all modelled scenarios. Compared to the current number of TDF/FTC initiations of ~600,000/year, our TDF/FTC scale-up scenario and conservative CAB and LEN scenarios would increase initiations to up to 2.4 million initiations per year by 2045- an average of 1.7 million annually (Fig 1). The optimistic CAB and LEN scale-up scenarios required between 900,000 and 4.4 million initiations per year (average 2.9 million annually). As CAB and LEN have different dosing frequencies and assumptions regarding continuation duration, the number of doses required differs between scenarios (Fig 1).

**Fig 2:**
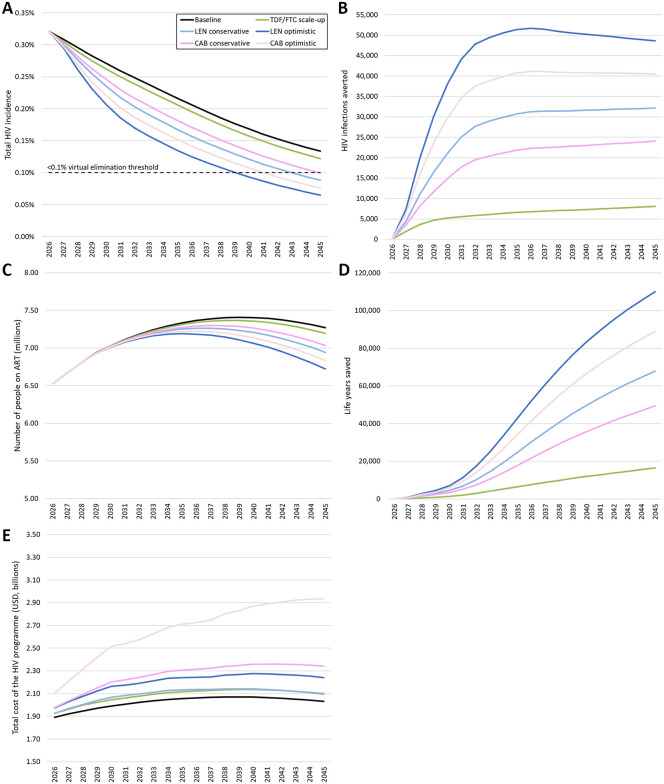
Impact of PrEP scale-up on (A) annual total HIV incidence, (B) HIV infections averted annually, (C) total number of people on ART, (D) life years saved over baseline, and (E) total HIV programme cost (in 2025 USD).

In the baseline scenario, population-wide HIV incidence was projected to continue declining and reach <0.1%, the virtual elimination threshold towards ending AIDS, by 2053. In the optimistic scenario, LEN scale-up accelerated this downward trajectory to reach <0.1% incidence by 2039, and avert on average 44,200 new HIV infections annually (Fig 2). Under conservative LEN scale-up, incidence reached <0.1% by 2043, and averted on average 26,900 infections/year (Fig 2). Injectable CAB averted slightly fewer infections, primarily because of the shorter continuation duration, with an average of 35,500 (optimistic) and 19,500 (conservative) infections averted annually. CAB reached <0.1% incidence in 2042 (optimistic) and 2045 (conservative). In comparison, TDF/FTC scale-up averted an average of 6,300 infections/year, and only reached <0.1% incidence by 2050 (Fig 2). Compared to baseline, LEN averted 19%−31% of HIV infections, and CAB averted 14%–25% of HIV infections over the 20-year period, and LEN saved 593,000–989,300 (3%–5%) life years, while CAB saved 428,700–792,600 (2%–4%) life years (Table 2). The number of people needed to initiate PrEP to avert one HIV infection, after the initial 4-year scale-up period, was ~280 initiations for TDF/FTC, and considerably less under LEN (conservative: ~65; optimistic: ~35) and CAB (conservative: ~125; optimistic: ~45) (Fig A in S1 Appendix).

**Table 2:** Impact and cost-effectiveness estimates* of oral TDF/FTC, injectable LEN or CAB scale-up compared with baseline over 2026–45.

	Total Cost of the HIV programme		Incremental cost-effectiveness		New HIV infections		Life years lost due to AIDS	
Scenario	Cost (billions, USD)	Incremental cost over baseline, %	Cost per infection averted (USD)	Cost per life year saved (USD)	Number (millions)	% averted over baseline	Number (millions)	% saved over baseline
**Baseline**	40.49	–	–	–	2.72	–	19.89	–
**Oral TDF/FTC scale-up**	41.68	3%	$9,907	$8,143	2.60	4%	19.74	1%
**Lenacapavir**								
Conservative	41.85	3%	$2,666	$2,301	2.21	19%	19.29	3%
Optimistic	44.02	9%	$4,202	$3,567	1.88	31%	18.90	5%
**Cabotegravir**								
Conservative	45.25	12%	$12,877	$11,114	2.35	14%	19.46	2%
Optimistic	53.26	32%	$18,918	$16,118	2.05	25%	19.09	4%

*Results represent deterministic point estimates from the central scenario. Probabilistic sensitivity analysis results with 95% uncertainty bounds are presented in Table H in S1 Appendix. Abbreviations: AIDS = Acquired Immunodeficiency Syndrome; CAB = cabotegravir; HIV = human immunodeficiency virus; LEN = lenacapavir; TDF/FTC = tenofovir/emtricitabine; USD = United States Dollars.

### Cost and cost-effectiveness of LEN and CAB

The cost of TDF/FTC provision was $57–$72 per person initiated, while the cost of LEN and CAB provision ranged from $60–$137 and $146–$388 per person initiated, respectively. Costs varied by population and were highly dependent on the assumed duration of the product. The primary cost driver was drugs for LEN (65%) and CAB (69%), while staff costs (36%) were the largest component for TDF/FTC (Table D in S1 Appendix). Both TDF/FTC and conservative LEN scale-up increased total HIV programme cost by 3%; however, LEN resulted in substantially greater health impact, rendering it more cost-effective (Table 2). The ICER for scaling-up LEN was $2,666–$4,202/infection averted and $2,301–$3,567/life year saved (LYS), compared to TDF/FTC with $9,907/infection averted and $8,143/LYS (Table 2). CAB was less cost-effective than both LEN and oral TDF/FTC scale-up with an ICER of $12,877–$18,918/infection averted and $11,114–$16,118/LYS over baseline (Table 2).

Compared to further scaling-up other HIV interventions, LEN had a larger impact on both life years saved and infections averted than most, second only to scaling up ART coverage to 95% (Table 3). However, it was only moderately cost-effective, ranking 6th (conservative) and 8th (optimistic) among modelled interventions when comparing cost per LYS (Table 3). Interventions more cost-effective than LEN under this metric were scaling-up condoms (cost-saving), ART coverage, HIV testing of the general population, MMC, and HIV self-screening, with ICERs ranging from $577–$1,081/LYS. In terms of cost per infection averted, LEN ranked fourth, but remained less cost-effective than condoms, MMC, and ART (Table 3).

**Table 3:** Cost-effectiveness league table comparing 20-year impact (2026-2045) and cost-effectiveness of LEN and CAB scale-up scenarios to existing HIV interventions, with incremental cost-effectiveness ratios compared to baseline.

Intervention	Incremental cost over baseline, USD millions (% change)	Incremental life years saved over baseline (% change)	Incremental HIV infections averted over baseline (% change)	Cost per life year saved (USD)	*Rank (cost/ LYS)*	Cost per HIV averted (USD)	*Rank (cost/ HIV)*
Condom distribution (725 m/year)	−301 (−0.7%)	348,165 (1.8%)	260,263 (9.6%)	Cost-saving	*1*	Cost-saving	*1*
ART (95%)	2,752 (6.8%)	4,770,659 (24.0%)	1,414,990 (52.0%)	577	*2*	1,945	*3*
General HTS (18 m/year)	149 (0.4%)	167,249 (0.8%)	38,844 (1.4%)	889	*3*	3,827	*5*
MMC (500 k/year)	103 (0.3%)	101,903 (0.5%)	101,324 (3.7%)	1,007	*4*	1,012	*2*
HIV self-screening package (1 m/year)	213 (0.5%)	196,712 (1.0%)	35,935 (1.3%)	1,081	*5*	5,917	*7*
LEN conservative (avg 1.8 m/year)	1,365 (3.4%)	593,025 (3.0%)	511,955 (18.8%)	2,301	*6*	2,666	*4*
Infant PCR testing at 10 weeks (95%)	18 (<0.1%)	6,412 (<0.1%)	17 (<0.1%)	2,850	*7*	1,074,866	*12*
LEN optimistic (avg 2.9 m/year)	3,529 (8.7%)	989,334 (5.0%)	839,898 (30.9%)	3,567	*8*	4,202	*6*
Oral TDF/FTC (avg 1.8 m/year)	1,189 (2.9%)	145,977 (0.7%)	119,982 (4.4%)	8,143	*9*	9,907	*8*
Infant rapid HIV testing at 18 months (95%)	76 (0.2%)	8,770 (<0.1%)	496 (<0.1%)	8,645	*10*	152,862	*11*
CAB conservative (avg 1.8 m/year)	4,764 (11.8%)	428,674 (2.2%)	369,997 (13.6%)	11,114	*11*	12,877	*9*
CAB optimistic (avg 2.9 m/year)	12,775 (31.6%)	792,593 (4.0%)	675,315 (24.8%)	16,118	*12*	18,918	*10*
Infant PCR testing at 6 months (95%)	66 (0.2%)	1,484 (<0.1%)	55 (<0.1%)	44,192	*13*	1,192,390	*13*

Abbreviations: ART = antiretroviral therapy; avg = average; CAB = cabotegravir; HIV = human immunodeficiency virus; HTS = HIV testing services; ICER = incremental cost-effectiveness ratio; k = thousand; LEN = lenacapavir; LYS = life years saved; m = million; MMC = medical male circumcision; PCR = polymerase chain reaction; TDF/FTC = tenofovir disoproxil fumarate/emtricitabine; USD = United States dollars.

### Budget impact

The conservative LEN scale-up scenario required an additional $38–$91 million/year (2%–5% over the current projected HIV programme cost) over financial years 2025/26–2029/30 (Table E in S1 Appendix). This incremental cost was similar to the TDF/FTC scale-up scenario, but with a substantially larger impact (Table 2). Under an optimistic scenario, $84–$198 million/year (4%−10% increase) was required for scale-up (Table E in S1 Appendix).

### Optimisation of LEN to subpopulations and most cost-effective long-term strategies

**Fig 3:**
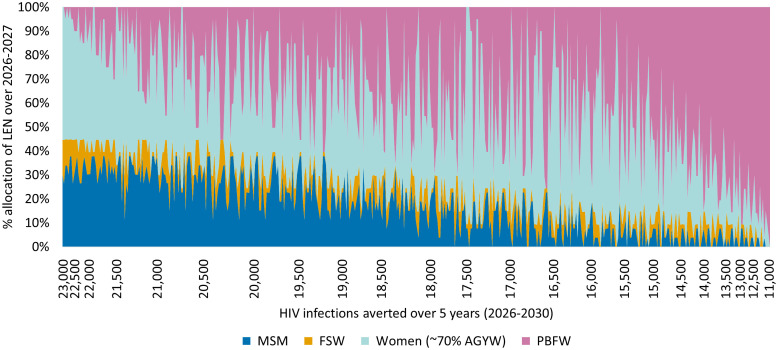
Five-year impact of different combinations of subpopulation uptake of LEN for initial allocation volume (~500,000 person-years on LEN) over 2026-2027. Each of the 490 different combinations of LEN distribution is represented by vertical bars, with all results sorted by descending order of impact; therefore, the leftmost combinations represent the highest impact strategies. The complete, unedited allocation metrics and infection outcomes for all modelled runs are provided in S1 Data.

The highest impact allocation, of 500,000 PYs of LEN delivered in 2026−2027, was 55% of LEN doses allocated to women (within these, 70% were AGYW), 30% to MSM, 15% to FSW, and 0% to PBFW (Fig 3). This averted ~23,100 new infections over 2026−2030 relative to baseline and a 19% improvement on the NDOH planned targets. However, alternative allocation strategies that included PBFW had a comparable impact. When incrementally shifting LEN initiation towards PBFW (10% increments representing ~25,000 initiations annually), LEN would avert between 20,800 and 22,700 infections (8%−17% more than the NDOH plan) (Table F in S1 Appendix). For a large-scale roll-out, the most cost-effective strategies over a 20-year time horizon were delivering to FSW only (cost-saving), FSW and MSM concurrently ($252/LYS and $290/infection averted), and MSM only ($331/LYS and $375/infection averted) (Table 4). Amongst strategies modelled, the largest impacts resulted from including LEN scale-up to pregnant women (in addition to FSW and MSM) and AGYW with ICER values ranging $1,102–$2,220/LYS and $1,380–$1,582/infection averted (Table 4).

**Table 4:** Impact and cost-effectiveness of specific subpopulation uptake of LEN, compared to baseline, over 20 years (2026-2045).

Scenario	Total cost of the HIV programme *(USD, billions)*	Incremental cost over baseline *(USD, millions)*	Life years lost to AIDS *(millions)*	Total new HIV infections *(millions)*	Incremental life years saved *(thousands)*	Incremental HIV infections averted (thousands)	Cost per life year saved *(USD)*	Cost per HIV averted *(USD)*
Baseline	40.49	*–*	19.89	2.72	*–*	*–*	**–**	**–**
FSW	40.49	−1 (- < 0.01%)	19.82	2.67	62 (0.3%)	52 (1.9%)	Cost-saving	Cost-saving
FSW and MSM	40.54	54 (0.13%)	19.67	2.53	216 (1.1%)	187 (6.9%)	252	290
MSM	40.54	53 (0.13%)	19.72	2.58	162 (0.8%)	143 (5.2%)	331	375
FSW, MSM, and pregnant women	40.88	394 (0.97%)	19.53	2.44	358 (1.8%)	286 (10.5%)	1,102	1,380
FSW and AGYW	40.86	369 (0.91%)	19.60	2.45	282 (1.4%)	272 (10.0%)	1,305	1,354
AGYW	40.85	365 (0.90%)	19.65	2.49	232 (1.2%)	231 (8.5%)	1,577	1,582
PBFW	40.82	334 (0.82%)	19.74	2.62	150 (0.8%)	106 (3.9%)	2,220	3,149
Heterosexual men	41.05	565 (1.39%)	19.81	2.67	73 (0.4%)	50 (1.8%)	7,700	11,354

Abbreviations: AGYW = adolescent girls and young women; AIDS = acquired immunodeficiency syndrome; FSW = female sex workers; HIV = human immunodeficiency virus; ICER = incremental cost-effectiveness ratio; MSM = men who have sex with men; PBFW = pregnant and breastfeeding women; USD = United States dollars.

### Sensitivity analysis

One-way sensitivity analyses showed that the cost-effectiveness and impact of LEN, relative to TDF/FTC scale-up, were robust (Fig B in S1 Appendix). Across both conservative and optimistic scenarios, the cost/LYS ratio comparing LEN with TDF/FTC consistently remained below 1, confirming LEN’s cost-effectiveness advantage. Likewise, the LYS ratio remained above 1, indicating greater effectiveness of LEN. The parameters with the greatest influence on cost-effectiveness were cost and risk-stratified uptake, while LEN uptake rates and TDF/FTC duration had the biggest impact on LYS (Fig B in S1 Appendix). In addition, CAB remained less cost-effective than TDF/FTC scale-up, unless CAB prices were reduced or CAB use was more strongly targeted toward higher risk populations (Fig C in S1 Appendix). When risk-targeted LEN delivery within the AGYW population was assumed to be less efficient within the Phase 1 Global Fund-backed roll-out, the relative priority of AGYW versus PBFW became less clear; however, FSW and MSM consistently remained high-priority populations for achieving maximum impact (Fig D in S1 Appendix). In the Phase 2 national roll-out, varying high-risk uptake amongst AGYW and increasing service delivery costs for FSW and MSM, altered ICER magnitudes, but did not materially change the ranking of strategies, with prioritisation of FSW and MSM remaining most cost-effective strategies (Table G in S1 Appendix).

In a probabilistic sensitivity analysis, the relative impact of PrEP scenarios was not sensitive to uncertainty in several epidemic calibration and PrEP intervention parameters; the reduction of HIV infections for LEN (conservative UB: 13.8%−24.1%; optimistic UB: 25.5%−35.7%) and CAB (conservative UB: 10.0%−18.8%; optimistic UB: 20.1%−30.0%) still exceeded TDF/FTC (UB 3.4%−5.5%) (Table H in S1 Appendix). LEN remained the most cost-effective option with consistently lower ICERs but wider uncertainty (conservative UB: $803–$7,142/LYS; optimistic UB: $1,761–$9,897), compared to TDF/FTC (UB $5,863–$19,157/LYS) and CAB (conservative UB: $6,516–$28,606/LYS; optimistic UB: $10,455–$38,496) (Fig C in S1 Appendix). CAB was consistently less cost-effective than LEN in all simulations, and less cost-effective than TDF/FTC scale-up in 88% (conservative) and 99.8% (optimistic) of simulations (Fig C in S1 Appendix).

## Discussion

Widespread use of LEN among those with elevated exposure to HIV could substantially decrease HIV infections and accelerate efforts to end AIDS in South Africa, achieving the <0.1% incidence threshold required to end AIDS 10–14 years earlier than through continuing current intervention levels, and 7–11 years sooner than further aggressive TDF/FTC scale-up. At $40 PPPY and $17 for the loading dose and an average delivery cost of $30 PPPY, LEN scale-up options were more cost-effective than both TDF/FTC and CAB scale-up but increased the HIV programme medium-term budget by 4%–8%. CAB was the least cost-effective option, due to its high price.

While the newly negotiated price for LEN substantially improves affordability, large-scale roll-out would still require substantial additional investment, which may particularly strain national funding in the current context of reduced US foreign aid. Compared to other interventions, expanding ART to 95% coverage of all diagnosed PLHIV was the only intervention exhibiting greater cost-effectiveness and higher impact in terms of both life years (4.8-8-fold more life years saved) and HIV infections (1.7-2.8-fold more infections averted) compared to LEN scale-up. However, significant implementation challenges remain in achieving 95% ART coverage, and the feasibility and resource requirements of reaching this target are not well established. Although we did not explicitly compare interventions to budget constraints here, previous results imply that if HIV programme resources are threatened, maintaining people on ART should be safeguarded before scaling-up LEN [30]. Increasing condom distribution and MMC also remained more cost-effective prevention options than LEN and should be implemented alongside PrEP scale-up as part of a comprehensive HIV prevention strategy for South Africa; however, the overall incremental epidemiologic impact of further expanding these interventions was limited compared to LEN.

Similar to other modelling analyses, we assumed PrEP uptake would be risk-differentiated, reaching individuals at disproportionately higher risk among AGYW, PBFW, and heterosexual men. Even with universal access, such differentiated uptake has defined efficient and impactful oral PrEP programmes in some settings [12], but evidence is less clear in African epidemic settings. We tested the sensitivity of our findings to less efficient high-risk targeting within AGYW (i.e., more low risk individuals take up LEN), and found that the relative of priority in terms of biggest reduction in HIV infections averted comparing AGYW versus PBFW became less clear, though FSW and MSM consistently remained high priority. Delivery models reaching wider AGYW through sexually transmitted infection (STI) and other sexual and reproductive health services may be more efficient at reaching those at high risk compared to antenatal care (ANC) services to pregnant women, where providers may find it more challenging to differentiate clients.

Ambitious LEN coverage modelled among FSW and MSM may be difficult to attain, particularly in the current context. Termination of US donor funding has disproportionately affected key population services in South Africa, which were primarily managed by organisations supported by PEPFAR or the Global Fund. Key populations often face stigma, which complicates their utilisation of public health facilities, while PBFW are already served within public clinics that can provide LEN. Successful delivery of LEN to FSW and MSM will likely require the South African government to revive key population programming in a way that provides friendly and safe care, potentially through partnering with existing experienced implementing organisations. If doses prioritised for FSW and MSM in the strategic allocation described here are not delivered at the anticipated volumes, impact would be preserved best by allocating additional available stocks to AGYW at higher risk of acquisition and then PBFW in high incidence locations, rather than allowing product to go unused or expire. These lower volumes could be due to, for example, smaller populations sizes than modelled, lower uptake than modelled coverage targets, or lower continuation than assumed, all of which could result from the ongoing defunding of key population programmes. Learning and dynamically adapting programme implementation to reach those at highest risk of acquisition during early programme roll-out will be critical to ensure programmes attain impacts close to those suggested by our modelling. In addition, geospatial prioritisation of areas with the highest HIV incidence could enhance both the cost-effectiveness and overall impact of LEN implementation at scale [31]. Geographic prioritisation could also refine the Phase 1 LEN roll-out more broadly: in high-incidence settings, a greater share of the available doses could be directed towards populations facing the most acute risk, while in lower incidence regions, the marginal benefit of additional initiations may be limited across all subpopulations. In addition, the size of key populations may vary markedly by geography, with FSW and MSM being more concentrated in urban settings, potentially limiting the number of initiations available in lower-density or rural settings and concentrating the cost-effectiveness gains of FSW- and MSM-targeted strategies in urban delivery platforms.

Our costing approach differs from previous modelling studies of LEN in the region, with Wu and colleagues and Kaftan and colleagues using a provision cost of $8.55 per dose from a study in Kenya [32]. This cost is based on a streamlined 6-month dispensing model of oral PrEP with interim HIV self-testing. While this approach is valid for modelling efficiency gains, it may underestimate the costs of implementing new injectable products at scale in routine settings. Our ingredients-based approach resulted in an average provision cost of $30 PPPY (excluding drugs) in the routine public-sector clinics, and indicates that, despite these higher delivery cost estimates, LEN remains highly cost-effective at the negotiated price of $40 PPPY, suggesting its value proposition is robust under conservative costing assumptions. Furthermore, while our findings provide useful and practical information for policymakers, our cost-effectiveness results may not be generalisable to other countries in eastern and southern Africa, as South Africa’s health system is fundamentally different in terms of healthcare capacity, domestic funding and resources, as well as underlying HIV incidence and risk-group dynamics. This has been reflected by the variation in price thresholds estimated across the region [11,12]. Country-specific analyses would better capture the programmatic priorities of LEN roll-out in other settings.

Our analysis is subject to several limitations. First, the feasibility of delivering our modelled scenarios is currently unknown. We included costs for demand creation, but these may not be sufficient to reach the assumed large-scale uptake. Though clients could be reached through existing platforms within primary and antenatal healthcare, South Africa may need to re-establish key population services for reaching MSM and FSW, and this may require additional resources. The volumes modelled are large, and could put additional pressure on the delivery and health infrastructure overall. Several implementation constraints are not captured structurally in our model, including clinic throughput, availability of trained personnel, and consumable supply continuity. Should any of these constraints bind in practice, realised coverage would fall below modelled levels, reducing infections averted and narrowing LEN’s cost-effectiveness advantage over oral PrEP. Second, with limited data available on costs of implementing long-acting products, we opted for an ingredients-based approach. The biggest cost difference between PrEP options lies in the drugs, but further research is needed to determine the true implementation costs and explore innovative delivery methods for injectable PrEP to enhance its cost-effectiveness, especially amongst key populations. However, sensitivity analyses varying delivery costs up to 3-fold did not materially alter the cost-effectiveness advantage of targeting key populations. Third, while we did vary LEN use duration from 6 to 24 months across scenarios and populations, we assumed that AGYW, PBFW, and heterosexual men would maintain similar duration of use across scenarios, while MSM had longer durations, loosely based on what we know from the oral PrEP programme. In reality, persistence may differ by subgroup and this differential persistence could shift the optimal targeting of LEN. Real-world data from the upcoming roll-out will be critical to better characterising population-specific persistence and refining these assumptions in future modelling. Fourth, our modelling assumed continuation of a baseline trajectory and intervention coverages that pre-date recent funding cuts. While the relative comparisons between interventions and overall conclusions remain valid, key populations were disproportionately affected by funding cuts and may have experienced increased HIV incidence as a result of disrupted prevention services [30]. A higher incidence would translate to greater reductions of HIV if effective prevention were scaled up in these populations. Fifth, we have used a frequency-dependent compartmental model to represent heterogeneity in HIV risk; such models are likely to understate the fraction of transmission that can be prevented by targeting interventions to high-risk groups [33]. This might partially explain why our modelled impacts of injectable PrEP are lower than those estimated using network models [34]. Stansfield and colleagues also show that Thembisa is more conservative in its estimates of effective coverage than those in comparable models such as Synthesis or EMOD, suggesting that Thembisa’s assumptions about risk-differentiated uptake are more conservative than those of comparable models, meaning our estimates of infections averted may already represent a cautious lower bound [34].

Immediate and concurrent monitoring and evaluation of the upcoming LEN roll-out are essential to generate evidence of uptake patterns and preferences, especially across different subpopulations. Such evidence is needed to inform efficient scale-up strategies as the PrEP product landscape continues to expand. As the LEN roll-out and continued insights will help guide national decision-making, South Africa’s scale and epidemic burden mean it is not merely a participant in the PrEP market – it is a market shaper whose adoption choices will influence global pricing and supply trajectories.

## Supporting information

S1 Data Model output data of different combinations of subpopulation distribution of ~500,000 person-years on LEN over 2026–2027 and new HIV infections over 2026–2030. Model estimates represent the outputs plotted in Fig 3 in the main text.
(XLSX)

S1 Checklist Consolidated health economic evaluation reporting standards 2022 (CHEERS 2022) Checklist. (Husereau D, Drummond M, Augustovski F, de Bekker-Grob E, Briggs AH, Carswell C, Caulley L, Chaiyakunapruk N, Greenberg D, Loder E, Mauskopf J, Mullins CD, Petrou S, Pwu RF, Staniszewska S; CHEERS 2022 ISPOR Good Research Practices Task Force. Consolidated Health Economic Evaluation Reporting Standards 2022 (CHEERS 2022) statement: updated reporting guidance for health economic evaluations. *BMJ*. 2022 Jan 11;376:e067975. https://doi.org/10.1136/bmj-2021-067975. PMID: 35017145; PMCID: PMC8749494.).
(DOCX)

S1 Appendix Includes the following Table A–H and Figure A–E. **Table A.** Key assumptions on coverage, duration and effectiveness for main scenarios (expanded version). **Table B**. Subpopulation prioritisation: number of initiations assumed for each population. **Table C.** Probability distributions used for parameters varied in the probabilistic sensitivity analysis. **Table D.** Cost breakdown of each PrEP modality by population and duration. **Table E.** Budget impact analysis of LEN scale-up. **Table F.** Selected distribution scenarios of LEN allocation of ~500,000 person-years on LEN over 2026–27 for maximum impact on HIV infections. **Table G.** Sensitivity of high-risk uptake and service delivery costs for FSW and MSM on the cost-effectiveness of subpopulation uptake of LEN, compared to baseline, over 20 years (2026–2045). **Table H.** Median, lower, and upper uncertainty bounds around the impact and cost-effectiveness of TDF/FTC, LEN, and CAB over a 20-year time horizon (2026–2045); based on 1,000 Monte Carlo simulations in a probabilistic sensitivity analysis. **Fig A.** (A) Number of people needed to initiate, and (B) doses required, to avert one HIV infection, by scenario. Acronyms: CAB = cabotegravir, LEN = lenacapavir, TDF/FTC = tenofovir disoproxil fumarate/emtricitabine. **Fig B.** Relative impact and cost-effectiveness comparing LEN and TDF/FTC scale-up in a one-way sensitivity analysis varying key risk, uptake and cost parameters. Results depict the ratio of cost per LYS in (A) conservative and (B) optimistic scenarios, and ratio of life years saved in LEN versus TDF/FTC scale-up in (C) conservative and (D) optimistic scenarios. Acronyms: LEN = lenacapavir, TDF/FTC = tenofovir disoproxil fumarate/emtricitabine, ICER = incremental cost-effectiveness ratio, LYS = life years saved, PPPY = per person per year. **Fig C.** Relative impact and cost-effectiveness comparing CAB and TDF/FTC scale-up in a sensitivity analysis varying key risk, uptake and cost parameters. Results depict the ratio of cost per LYS in (A) conservative and (B) optimistic scenarios, and ratio of life years saved in CAB versus TDF/FTC scale-up in (C) conservative and (D) optimistic scenarios. Acronyms: CAB = cabotegravir, TDF/FTC = tenofovir disoproxil fumarate/emtricitabine, ICER = incremental cost-effectiveness ratio, LYS = life years saved, PPPY = per person per year. **Fig D.** One-way sensitivity analysis of 5-year impact of different combinations of subpopulation uptake when (A) reducing, and (B) increasing the rate of high-risk uptake of LEN within the general population. Total LEN is constrained to (~500,000 person-years on LEN) over 2026–2027. Each of the 490 different combinations of LEN distribution is represented by vertical bars, with all results sorted by descending order of impact; therefore, the leftmost combinations represent the highest impact strategies. **Fig E.** Probabilistic sensitivity analysis results comparing the incremental cost per LYS over 2026–2045 for TDF/FTC scale-up to that of the LEN (A) conservative and (B) optimistic scale-up scenarios, and the CAB (C) conservative, and (D) optimistic scale-up scenarios. Each dot represents a single simulation of a total of 1,000 simulations, the diagonal line represents equal cost-effectiveness between TDF/FTC and LEN/CAB. The price of LEN was $40 PPPY (4 injections) and $17 for the loading dose tablets; price of CAB is the current manufacturer offered price of $180–210 PPPY (6–7 injections). Acronyms: CAB = cabotegravir, LEN = lenacapavir, TDF/FTC = tenofovir disoproxil fumarate/emtricitabine, USD = United States Dollars.
(DOCX)

## Abbreviations

AGYW: adolescent girls and young women
ANC: antenatal care
ART: antiretroviral treatment
CAB: cabotegravir
FSW: female sex workers
ICER: incremental cost-effectiveness ratio
LEN: lenacapavir
LYS: life year saved
MMC: medical male circumcision
MSM: men who have sex with men
NDOH: National Department of Health
PBFW: pregnant and breastfeeding women
PCR: polymerase chain reaction
PLHIV: people living with HIV
PPPY: per person per year
PrEP: pre-exposure prophylaxis
STI: sexually transmitted infection
TDF/FTC: fumarate/emtricitabine
UB: uncertainty bounds
USD: United States Dollars.

## References

[1] Carter A, Zhang M, Tram KH, Walters MK, Jahagirdar D, Brewer ED. Global, regional, and national burden of HIV/AIDS, 1990–2021, and forecasts to 2050, for 204 countries and territories: the Global Burden of Disease Study 2021. The Lancet HIV. 2024;11:e807–22. doi: 10.1016/S2352-3018(24)00212-1 PMC11612058 39608393

[2] UNAIDS. 2025 Global AIDS Update — AIDS, crisis and the power to transform. Available from: https://www.unaids.org/en/resources/documents/2025/2025-global-aids-update. Accessed 2025 July 18.

[3] Kelley CF, Acevedo-Quiñones M, Agwu AL, Avihingsanon A, Benson P, Blumenthal J, et al. Twice-yearly lenacapavir for HIV prevention in men and gender-diverse persons. N Engl J Med. 2025;392(13):1261–76. doi: 10.1056/NEJMoa2411858 39602624

[4] Bekker L-G, Das M, Abdool Karim Q, Ahmed K, Batting J, Brumskine W, et al. Twice-yearly lenacapavir or daily F/TAF for HIV prevention in cisgender women. N Engl J Med. 2024;391(13):1179–92. doi: 10.1056/NEJMoa2407001 39046157

[5] SAHPRA Registers Lenacapavir. Available from: https://www.sahpra.org.za/news-and-updates/sahpra-registers-lenacapavir/. Accessed 2025 October 29.

[6] Malan M. SA gets R520-million to buy the twice-a-year anti-HIV jab — but there’s a snag. In: Bhekisisa. Available from: Available from: https://bhekisisa.org/health-news-south-africa/2025-07-15-sa-gets-r520-million-to-buy-the-twice-a-year-anti-hiv-jab-but-theres-a-snag/. 2025. Accessed 2025 October 3.

[7] Unitaid, CHAI, Wits RHI. Unitaid, CHAI, and Wits RHI enter into a landmark agreement with Dr. Reddy’s to make HIV prevention tool lenacapavir affordable in LMICs. Available from: https://unitaid.org/news-blog/lenacapavir-for-hiv-prevention/. Accessed 2025 October 29.

[8] Hill A, Levi J, Fairhead C, Pilkington V, Wang J, Johnson M, et al. Lenacapavir to prevent HIV infection: current prices versus estimated costs of production. J Antimicrob Chemother. 2024;79(11):2906–15. doi: 10.1093/jac/dkae305 39225016

[9] Fairhead C, Fortunak J, Layne J, Johnson M, Smalley S, Lutterodt A, et al. 174. Generic lenacapavir HIV Pre-exposure prophylaxis could be produced for $25 per person per Year. Open Forum Infect Dis. 2026;13:ofaf695.004. doi: 10.1093/ofid/ofaf695.004

[10] Meyer-Rath G, Jamieson L, Mudimu E, Snyman K, Ong JJ, Corlis J, et al. Who pays and what pays off in sexual and reproductive health? A review of the cost and cost-effectiveness of interventions and implications for future funding and markets. Lancet. 2025;406(10515):2152–67. doi: 10.1016/S0140-6736(25)01724-6 41176394

[11] Wu L, Kaftan D, Wittenauer R, Arrouzet C, Patel N, Saravis AL, et al. Health impact, budget impact, and price threshold for cost-effectiveness of lenacapavir for HIV pre-exposure prophylaxis in eastern and southern Africa: a modelling analysis. Lancet HIV. 2024;11(11):e765–73. doi: 10.1016/S2352-3018(24)00239-X 39312933 PMC11519315

[12] Kaftan D, Sharma M, Resar D, Milali M, Mudimu E, Wu L, et al. Cost thresholds for anticipated long-acting HIV pre-exposure prophylaxis products in Eastern and Southern Africa: a mathematical modelling study. J Int AIDS Soc. 2025;28(2):e26427. doi: 10.1002/jia2.26427 39995017 PMC11850439

[13] Johnson L, Dorrington R. Thembisa version 4.8: a model for evaluating the impact of HIV/AIDS in South Africa. 2025. Available from: https://thembisa.org/content/downloadPage/Thembisa4_8report

[14] Molina J-M, Capitant C, Spire B, Pialoux G, Cotte L, Charreau I, et al. On-demand preexposure prophylaxis in men at high risk for HIV-1 infection. N Engl J Med. 2015;373(23):2237–46. doi: 10.1056/NEJMoa1506273 26624850

[15] McCormack S, Dunn DT, Desai M, Dolling DI, Gafos M, Gilson R, et al. Pre-exposure prophylaxis to prevent the acquisition of HIV-1 infection (PROUD): effectiveness results from the pilot phase of a pragmatic open-label randomised trial. Lancet. 2016;387(10013):53–60. doi: 10.1016/S0140-6736(15)00056-2 26364263 PMC4700047

[16] Bekker L-G, Roux S, Sebastien E, Yola N, Amico KR, Hughes JP, et al. Daily and non-daily pre-exposure prophylaxis in African women (HPTN 067/ADAPT Cape Town Trial): a randomised, open-label, phase 2 trial. Lancet HIV. 2018;5(2):e68–78. doi: 10.1016/S2352-3018(17)30156-X 28986029 PMC6107917

[17] Delany-Moretlwe S, Hughes JP, Bock P, Ouma SG, Hunidzarira P, Kalonji D, et al. Cabotegravir for the prevention of HIV-1 in women: results from HPTN 084, a phase 3, randomised clinical trial. Lancet. 2022;399(10337):1779–89. doi: 10.1016/S0140-6736(22)00538-4 35378077 PMC9077443

[18] Landovitz RJ, Donnell D, Clement ME, Hanscom B, Cottle L, Coelho L, et al. Cabotegravir for HIV prevention in cisgender men and transgender women. N Engl J Med. 2021;385:595–608. doi: 10.1056/NEJMoa2101016 34379922 PMC8448593

[19] Landovitz RJ, Li S, Eron JJ Jr, Grinsztejn B, Dawood H, Liu AY, et al. Tail-phase safety, tolerability, and pharmacokinetics of long-acting injectable cabotegravir in HIV-uninfected adults: a secondary analysis of the HPTN 077 trial. Lancet HIV. 2020;7(7):e472–81. doi: 10.1016/S2352-3018(20)30106-5 32497491 PMC7859863

[20] Pfau B, Saravis A, Cox SN, Wu L, Wittenauer R, Callen E, et al. User preferences on long-acting pre-exposure prophylaxis for HIV prevention in Eastern and Southern Africa: a scoping review. BMC Public Health. 2025;25(1):2361. doi: 10.1186/s12889-025-23529-y 40611019 PMC12225200

[21] Delany-Moretlwe S. Initial PrEP product choice: results from the HPTN 084 open-label extension. In: Brisbane. 2023. Available from: ps://www.hptn.org/sites/default/files/inline-files/220725%20IAS%202023%20product%20choice%20revised.pdf

[22] Health Economics and Epidemiology Research Office (HE2RO). South African HIV investment case: 2023 full report. 2023. Available from: https://www.heroza.org/publications/south-african-hiv-investment-case/

[23] Chiu C, Johnson LF, Jamieson L, Larson BA, Meyer-Rath G. Designing an optimal HIV programme for South Africa: does the optimal package change when diminishing returns are considered? BMC Public Health. 2017;17(1):143. doi: 10.1186/s12889-017-4023-3 28143525 PMC5282636

[24] Jamieson L, Johnson LF, Matsimela K, Sande LA, d’Elbée M, Majam M, et al. The cost effectiveness and optimal configuration of HIV self-test distribution in South Africa: a model analysis. BMJ Glob Health. 2021;6(Suppl 4):e005598. doi: 10.1136/bmjgh-2021-005598 34275876 PMC8287627

[25] South African Reserve Bank. Selected historical rates. Available from: https://www.resbank.co.za/en/home/what-we-do/statistics/key-statistics/selected-historical-rates. 2025. Accessed 2025 April 14.

[26] Jamieson L, Johnson LF, Nichols BE, Delany-Moretlwe S, Hosseinipour MC, Russell C, et al. Relative cost-effectiveness of long-acting injectable cabotegravir versus oral pre-exposure prophylaxis in South Africa based on the HPTN 083 and HPTN 084 trials: a modelled economic evaluation and threshold analysis. Lancet HIV. 2022;9(12):e857–67. doi: 10.1016/S2352-3018(22)00251-X 36356603 PMC9708606

[27] Two drugmakers will sell the 6-monthly anti-HIV jab for the price of the daily HIV prevention pill. Available from: https://bhekisisa.org/health-news-south-africa/2025-09-24-two-drugmakers-will-sell-the-6-monthly-anti-hiv-jab-for-the-price-of-the-daily-hiv-prevention-pill/. Accessed 2025 October 3.

[28] Tomlinson C. In the spotlight | HIV prevention injections exist, but hardly anyone can get them. Spotlight. Available from: https://www.spotlightnsp.co.za/2024/06/11/inthespotlight-hiv-prevention-injections-exist-but-hardly-anyone-can-get-them/. 2024. Accessed 2025 September 10.

[29] Meyer-Rath G, van Rensburg C, Chiu C, Leuner R, Jamieson L, Cohen S. The per-patient costs of HIV services in South Africa: systematic review and application in the South African HIV Investment Case. PLoS One. 2019;14(2):e0210497. doi: 10.1371/journal.pone.0210497 30807573 PMC6391029

[30] Meyer-Rath G, Jamieson L, Mudimu E, Imai-Eaton JW, Johnson LF. The cost of the plunge: the impact and cost of a cessation of PEPFAR-supported services in South Africa. AIDS. 2025;39(10):1476–80. doi: 10.1097/QAD.0000000000004272 40549501 PMC12262123

[31] Anderson S-J, Cherutich P, Kilonzo N, Cremin I, Fecht D, Kimanga D, et al. Maximising the effect of combination HIV prevention through prioritisation of the people and places in greatest need: a modelling study. Lancet. 2014;384(9939):249–56. doi: 10.1016/S0140-6736(14)61053-9 25042235

[32] Mangale DI, Heitner J, Ortblad KF, Mogere P, Kiptinness C, Mugo NR, et al. Opportunity for cost savings with a novel differentiated model of PrEP delivery: a comparative costing analysis of six-month PrEP supported by interim HIV self-testing and standard of care PrEP dispensing in Kenya. BMC Health Serv Res. 2025;25(1):865. doi: 10.1186/s12913-025-12891-7 40598383 PMC12220747

[33] Johnson LF, Geffen N. A comparison of two mathematical modeling frameworks for evaluating sexually transmitted infection epidemiology. Sex Transm Dis. 2016;43:139–46. doi: 10.1097/OLQ.0000000000000412 26859800

[34] Stansfield SE, Moore M, Jamieson L, Meyer-Rath G, Johnson LF, Kaftan D, et al. Estimated impact of long-acting injectable PrEP in South Africa: a model comparison analysis. J Int AIDS Soc. 2025;28 Suppl 2(Suppl 2):e26453. doi: 10.1002/jia2.26453 40600502 PMC12215805

[35] Jamieson L, Gomez GB, Rebe K, Brown B, Subedar H, Jenkins S, et al. The impact of self-selection based on HIV risk on the cost-effectiveness of preexposure prophylaxis in South Africa. AIDS. 2020;34(6):883–91. doi: 10.1097/QAD.0000000000002486 32004205

