# Supplementary material for: Optimising scale-up of injectable lenacapavir for HIV pre-exposure prophylaxis in South Africa: A modelling study and economic evaluation
Source: PLoS Med. 2026 Jul 21;23(7):e1004882. doi: 10.1371/journal.pmed.1004882 (PMC13421763; doi:10.1371/journal.pmed.1004882)
Supplement: S1 Checklist — (Husereau D, Drummond M, Augustovski F, de Bekker-Grob E, Briggs AH, Carswell C, Caulley L, Chaiyakunapruk N, Greenberg D, Loder E, Mauskopf J, Mullins CD, Petrou S, Pwu RF, Staniszewska S; CHEERS 2022 ISPOR Good Research Practices Task Force. Consolidated Health Economic Evaluation Reporting Standards 2022 (CHEERS 2022) statement: updated reporting guidance for health economic evaluations. BMJ. 2022 Jan 11;376:e067975. https://doi.org/10.1136/bmj-2021-067975. PMID: 35017145; PMCID: PMC8749494.). (DOCX) [file pmed.1004882.s002.docx]

# S1 Checklist: CHEERS

**Consolidated Health Economic Evaluation Reporting Standards 2022 (CHEERS 2022) Checklist**

| **Topic** | **No.** | **Item** | **Location where item is reported** | **Text excerpt or explanation** |
| --- | --- | --- | --- | --- |
| **Title** |  |  |  |  |
|  | 1 | Identify the study as an economic evaluation and specify the interventions being compared. | Title | Title: Taking injectable PrEP to scale: A modelling study and economic evaluation to optimise lenacapavir for South Africa’s HIV response |
| **Abstract** |  |  |  |  |
|  | 2 | Provide a structured summary that highlights context, key methods, results, and alternative analyses. | Abstract, Page 1 | See page 1 for detailed description of Why Was This Study Done, What Did the Researchers Do and Find, and What Do These Findings Mean. |
| **Introduction** |  |  |  |  |
| **Background and objectives** | 3 | Give the context for the study, the study question, and its practical relevance for decision making in policy or practice. | Introduction, Paragraph 3 and 5 | “To inform the prioritization of the planned roll-out of LEN in the South Africa, we conducted three economic evaluations: (1) we estimated the cost and cost-effectiveness of a large-scale roll-out of LEN at the generic price of $40 PPPY, compared to CAB and oral TDF/FTC scale-up, and produced budget estimates for the South African government; (2) we compared both LEN and CAB roll-out to scaling up other existing HIV interventions, including testing, prevention and treatment; and (3) we modelled several roll-out strategies to different subpopulations to optimise the epidemiologic impact of the initial 2-year LEN allocation (~500,000 person-years annually in 2026 and 2027) (Phase 1) and the planned large-scale roll-out from 2028 onwards (Phase 2).” |
| **Methods** |  |  |  |  |
| **Health economic analysis plan** | 4 | Indicate whether a health economic analysis plan was developed and where available. | N/A | A formal, pre-registered health economic analysis plan was not developed for this modeling study. Instead, the complete health economic framework, evaluation scenarios, and costing methodologies were prospective and are detailed comprehensively in the Methods section under the 'Scenarios and assumptions' and 'Cost analysis' subsections. |
| **Study population** | 5 | Describe characteristics of the study population (such as age range, demographics, socioeconomic, or clinical characteristics). | Methods, Pages 5-6 | “The model population is stratified by age, sex, sexual experience, sexual behaviour, marital status, HIV testing history and male circumcision status. The sexually experienced population is divided into two broad sexual risk groups: ‘high-risk’ (people who engage in concurrent partnerships and/or commercial sex) and ‘low-risk’; female sex workers (FSWs) are modelled as a subset of high-risk unmarried women, and men who have sex with men (MSM) are modelled as subsets of the unmarried low-risk and high-risk groups.”  “For the first analysis, we modelled LEN, CAB and TDF/FTC scale-up over a 20-year time horizon, starting from 2026, with uptake in all women, particularly adolescent girls and young women (AGYW) aged 15-24 years, pregnant and breastfeeding women (PBFW), FSW, MSM and heterosexual men.” |
| **Setting and location** | 6 | Provide relevant contextual information that may influence findings. | Introduction, paragraph 3; Methods (cost analysis) | “South Africa largely funds its HIV programme from domestic resources, making considerations of cost-effectiveness and affordability paramount”  “Costs were analysed from the perspective of the South African government and reported in 2025 United States Dollar (USD; exchange rate 18.22 South African Rand (ZAR) per 1 USD).”  “Briefly, PrEP is provided in primary healthcare clinics and includes HIV testing, counselling, provision of condoms, syndromic management of sexually transmitted infections with treatment referral, training, outreach, mobilisation, monitoring and evaluation costs.” |
| **Comparators** | 7 | Describe the interventions or strategies being compared and why chosen. | Methods, Scenarios and assumptions section on pages 6-7 | Interventions and scenarios described in detail, with justification “These options capture South Africa's full current and emerging biomedical prevention landscape, helping decision-makers evaluate whether transitioning to newer long-acting formulations provides sufficient value-for-money compared to established regimens” |
| **Perspective** | 8 | State the perspective(s) adopted by the study and why chosen. | Methods, lines 252-253 | “Costs were analysed from the perspective of the South African government and reported in 2025 United States Dollar (USD; exchange rate 18.22 South African Rand (ZAR) per 1 USD).” |
| **Time horizon** | 9 | State the time horizon for the study and why appropriate. | Methods, lines 187, 228-230, 241-242 | “For the first analysis, we modelled LEN, CAB and TDF/FTC scale-up over a 20-year time horizon…”  “We modelled the impact of Phase 1 with the objective to maximize new HIV infections averted over a 5-year time horizon (2026-2030), reflecting primary and short-term secondary infections averted.”  “For Phase 2, we modelled a large-scale LEN roll-out delivered to each subpopulation separately, as well as selected combinations of mostly AGYW, PBFW, FSW and MSM, over a 20-year time horizon” |
| **Discount rate** | 10 | Report the discount rate(s) and reason chosen. | Methods lines 244-245 | “Costs are undiscounted, representing data of use for budgeting purposes.” |
| **Selection of outcomes** | 11 | Describe what outcomes were used as the measure(s) of benefit(s) and harm(s). | Methods lines 196-197 | “We evaluated impact on life years lost due to AIDS, new HIV infections and incremental cost effectiveness for each of these outcomes.” |
| **Measurement of outcomes** | 12 | Describe how outcomes used to capture benefit(s) and harm(s) were measured. | Methods Pages 5-6 | Paragraph starting with “We used Thembisa (version 4.8), a deterministic compartmental HIV transmission model of the South African HIV epidemic” describes how the model generates health outcomes: HIV acquisition as a function of partnerships/prevalence/risk group, product effectiveness assumptions, and the model's calibration approach. |
| **Valuation of outcomes** | 13 | Describe the population and methods used to measure and value outcomes. | Methods Pages 5-6 (model and scenarios described), pages 8-9 (cost analysis) | In addition to above, the ‘Scenarios and assumptions’ paragraph details the populations we are focused on and scenarios modelled and the ‘Cost analysis’ section describes the cost-effectiveness outcomes of interest: “We estimated cost-effectiveness over a 20-year time horizon (2026-2045) as the incremental cost per HIV infection averted and incremental cost per life year saved, compared to baseline.” |
| **Measurement and valuation of resources and costs** | 14 | Describe how costs were valued. | Methods Page 8, lines 251-267 (Cost analysis) | Paragraph starting with “Costs were analysed from the perspective of the South African government and reported in 2025 United States Dollar (USD; exchange rate 18.22 South African Rand (ZAR) per 1 USD) [25].” |
| **Currency, price date, and conversion** | 15 | Report the dates of the estimated resource quantities and unit costs, plus the currency and year of conversion. | Methods Page 8, lines 251-267 (Cost analysis) | Paragraph starting with “Costs were analysed from the perspective of the South African government and reported in 2025 United States Dollar (USD; exchange rate 18.22 South African Rand (ZAR) per 1 USD) [25].” |
| **Rationale and description of model** | 16 | If modelling is used, describe in detail and why used. Report if the model is publicly available and where it can be accessed. | Methods Page 5, Data sharing statement | “This modelling framework is particularly appropriate for a mature, large-scale epidemic, as it captures population-level dynamics while representing HIV acquisition, disease progression, and mortality. The compartmental structure allows integration of prevention and treatment interventions across heterogeneous risk groups, providing a transparent and reproducible basis for evaluating epidemiological impact and cost-effectiveness under different implementation scenarios.”  “Data availability: The C++ version of Thembisa used for this analysis is publicly available at https://doi.org/10.5281/zenodo.20744379” |
| **Analytics and assumptions** | 17 | Describe any methods for analysing or statistically transforming data, any extrapolation methods, and approaches for validating any model used. | Methods Page 5, lines 152-156 | “Thembisa 4.8 is fitted using a Bayesian approach in which prior distributions are specified for the parameters governing sexual behaviour, HIV transmission and disease progression, and then calibrated against several data sources for HIV prevalence (antenatal clinic data, national household surveys, FSW and MSM prevalence studies) as well as data on recorded deaths, antiretroviral metabolites and age distributions for adult ART patients.” |
| **Characterising heterogeneity** | 18 | Describe any methods used for estimating how the results of the study vary for subgroups. | Methods Pages 7 and 9 | Paragraph starting with “The third analysis modelled LEN strategies maximise the epidemiologic impact of (i) the current Global Fund LEN allocation (covering ~500,000 person-years over 2026-2027) (Phase 1) [6], and (ii) the large-scale roll-out (Phase 2).”  Sensitivity analysis paragraph (beginning "We conducted a one-way sensitivity analysis within our main analysis..."), specifically the sentence: "We also conducted sensitivity analysis to examine whether improved or poorer risk targeting, or higher service delivery costs for FSW or MSM, affected impact of different subpopulation distributions in Phase 1, or the relative cost-effectiveness of LEN scale-up in Phase 2..." |
| **Characterising distributional effects** | 19 | Describe how impacts are distributed across different individuals or adjustments made to reflect priority populations. | Methods Pages 7 | Paragraph starting with “The third analysis modelled LEN strategies maximise the epidemiologic impact of (i) the current Global Fund LEN allocation (covering ~500,000 person-years over 2026-2027) (Phase 1) [6], and (ii) the large-scale roll-out (Phase 2).” And more description for the Phase 2 modelling: “For Phase 2, we modelled a large-scale LEN roll-out delivered to each subpopulation separately, as well as selected combinations of mostly AGYW, PBFW, FSW and MSM, over a 20-year time horizon (2026-2045), and compared the impact and cost to baseline.” |
| **Characterising uncertainty** | 20 | Describe methods to characterise any sources of uncertainty in the analysis. | Methods Page 9 (Sensitivity analysis) | Sensitivity analysis paragraph starting with "We conducted a one-way sensitivity analysis within our main analysis...") |
| **Approach to engagement with patients and others affected by the study** | 21 | Describe any approaches to engage patients or service recipients, the general public, communities, or stakeholders (such as clinicians or payers) in the design of the study. | Not applicable | Not applicable |
| **Results** |  |  |  |  |
| **Study parameters** | 22 | Report all analytic inputs (such as values, ranges, references) including uncertainty or distributional assumptions. | Methods Page 5-9, Table 1, Appendix | Besides the description of analytical inputs, specific key assumptions are presented in Table 1 as well as the Supplementary appendix. |
| **Summary of main results** | 23 | Report the mean values for the main categories of costs and outcomes of interest and summarise them in the most appropriate overall measure. | Table 2; Results on Pages 10-11 (lines 300-358) | Summary results presented in Table 2. Several paragraphs in the text starting with “The numbers initiating PrEP increased substantially in all modelled scenarios. Compared to the current number of TDF/FTC initiations of ~600,000/year, our TDF/FTC scale-up scenario and conservative CAB and LEN scenarios would increase initiations to up to 2.4 million initiations per year by 2045- an average of 1.7 million annually (Figure 1).” |
| **Effect of uncertainty** | 24 | Describe how uncertainty about analytic judgments, inputs, or projections affect findings. Report the effect of choice of discount rate and time horizon, if applicable. | Page 12, lines 360-382 (Sensitivity Analysis); Appendix | Paragraph starting with “One-way sensitivity analyses showed that the cost-effectiveness and impact of LEN, relative to TDF/FTC scale-up, were robust (Figure S2).” |
| **Effect of engagement with patients and others affected by the study** | 25 | Report on any difference patient/service recipient, general public, community, or stakeholder involvement made to the approach or findings of the study | Not applicable | N/A |
| **Discussion** |  |  |  |  |
| **Study findings, limitations, generalisability, and current knowledge** | 26 | Report key findings, limitations, ethical or equity considerations not captured, and how these could affect patients, policy, or practice. | Pages 12-14 | Key findings, limitations, considerations for more vulnerable key populations are all included in the Discussion. |
| **Other relevant information** |  |  |  |  |
| **Source of funding** | 27 | Describe how the study was funded and any role of the funder in the identification, design, conduct, and reporting of the analysis | Lines 486-492 | Funding paragraph starting with “LJ, LFJ, JWI-E, HS and GMR were funded for this work by the Gates Foundation (grant number INV-063625) (www.gatesfoundation.org). HS and LGB received no funding for this manuscript.” |
| **Conflicts of interest** | 28 | Report authors conflicts of interest according to journal or International Committee of Medical Journal Editors requirements. | Lines 480-484 | “I have read the journal's policy and the authors of this manuscript have the following competing interests: LGB reports receiving institutional research funding from Gilead Sciences for clinical trials of lenacapavir. All other authors (LJ, LFJ, JWI-E, HS and GMR) have declared that no competing interests exist.” |

*The original checklist is available at: Husereau D, Drummond M, Augustovski F, de Bekker-Grob E, Briggs AH, Carswell C, Caulley L, Chaiyakunapruk N, Greenberg D, Loder E, Mauskopf J, Mullins CD, Petrou S, Pwu RF, Staniszewska S; CHEERS 2022 ISPOR Good Research Practices Task Force. Consolidated Health Economic Evaluation Reporting Standards 2022 (CHEERS 2022) statement: updated reporting guidance for health economic evaluations. BMJ. 2022 Jan 11;376:e067975. doi: 10.1136/bmj-2021-067975. PMID: 35017145; PMCID: PMC8749494.*
