# Supplementary material for: Optimising scale-up of injectable lenacapavir for HIV pre-exposure prophylaxis in South Africa: A modelling study and economic evaluation
Source: PLoS Med. 2026 Jul 21;23(7):e1004882. doi: 10.1371/journal.pmed.1004882 (PMC13421763; doi:10.1371/journal.pmed.1004882)
Supplement: S1 Appendix — Table A. Key assumptions on coverage, duration and effectiveness for main scenarios (expanded version). Table B. Subpopulation prioritisation: number of initiations assumed for each population. Table C. Probability distributions used for parameters varied in the probabilistic sensitivity analysis. Table D. Cost breakdown of each PrEP modality by population and duration. Table E. Budget impact analysis of LEN scale-up. Table F. Selected distribution scenarios of LEN allocation of ~500,000 person-years on LEN over 2026–27 for maximum impact on HIV infections. Table G. Sensitivity of high-risk uptake and service delivery costs for FSW and MSM on the cost-effectiveness of subpopulation uptake of LEN, compared to baseline, over 20 years (2026–2045). Table H. Median, lower, and upper uncertainty bounds around the impact and cost-effectiveness of TDF/FTC, LEN, and CAB over a 20-year time horizon (2026–2045); based on 1,000 Monte Carlo simulations in a probabilistic sensitivity analysis. Fig A. (A) Number of people needed to initiate, and (B) doses required, to avert one HIV infection, by scenario. Acronyms: CAB = cabotegravir, LEN = lenacapavir, TDF/FTC = tenofovir disoproxil fumarate/emtricitabine. Fig B. Relative impact and cost-effectiveness comparing LEN and TDF/FTC scale-up in a one-way sensitivity analysis varying key risk, uptake and cost parameters. Results depict the ratio of cost per LYS in (A) conservative and (B) optimistic scenarios, and ratio of life years saved in LEN versus TDF/FTC scale-up in (C) conservative and (D) optimistic scenarios. Acronyms: LEN = lenacapavir, TDF/FTC = tenofovir disoproxil fumarate/emtricitabine, ICER = incremental cost-effectiveness ratio, LYS = life years saved, PPPY = per person per year. Fig C. Relative impact and cost-effectiveness comparing CAB and TDF/FTC scale-up in a sensitivity analysis varying key risk, uptake and cost parameters. Results depict the ratio of cost per LYS in (A) conservative and (B) optimistic scenarios, [file pmed.1004882.s003.docx]

# S1 Appendix

**Optimising scale-up of injectable lenacapavir for HIV pre-exposure prophylaxis in South Africa: A modelling study and economic evaluation**

Lise Jamieson^*1,2^, Leigh F. Johnson^3^, Jeffrey W. Imai-Eaton^4,5^, Hasina Subedar^6^, Linda-Gail Bekker^7^, Gesine Meyer-Rath^1,2,8^

1. Health Economics and Epidemiology Research Office, School of Clinical Medicine, Faculty of Health Sciences, University of the Witwatersrand, Johannesburg, South Africa
2. South African Centre for Epidemiological Modelling and Analysis (SACEMA), Centre for Epidemic Response and Innovation (CERI), School for Data Science and Computational Thinking, Stellenbosch University, Stellenbosch, South Africa
3. Centre for Integrated Data and Epidemiological Research, School of Public Health, University of Cape Town, Cape Town, South Africa
4. Center for Communicable Disease Dynamics, Department of Epidemiology, Harvard TH Chan School of Public Health, Boston, Massachusetts, United States of America
5. MRC Centre for Global Infectious Disease Analysis, School of Public Health, Imperial College London, London, United Kingdom
6. National Department of Health, Pretoria, South Africa
7. Desmond Tutu HIV Centre, University of Cape Town, Cape Town, South Africa
8. Department of Global Health, Boston University School of Public Health, Boston, Massachusetts, United States of America

**Table A. Key assumptions on coverage, duration and effectiveness for main scenarios (expanded version)**

|  | **Scenarios** |  |  |  |  |  |  |
| --- | --- | --- | --- | --- | --- | --- | --- |
|  | **Baseline (TDF/FTC only)** | **TDF/FTC scale-up**  Doubled initiation rates compared to baseline | **LEN conservative scale-up**  *Same initiation rates as TDF/FTC scale-up,*  *6-12m duration* | **LEN optimistic scale-up**  *Doubled initiation rates than TDF/FTC scale-up,*  *12-24m duration* | **CAB conservative scale-up**  *Same initiation rates as TDF/FTC scale-up,*  *4-8m duration* | **CAB optimistic scale-up**  *Doubled initiation rates than TDF/FTC scale-up,*  *8-16m duration* | **Source** |
| **Coverage scenarios *(% coverage in population)*** |  |  |  |  |  |  |  |
| *Coverage of TDF/FTC in 2030* | *11% FSW* | *22% FSW* | *6% FSW* | *3% FSW* | *7% FSW* | *4% FSW* | [1] for baseline initiation rates, relative uptake between populations; other initiation rates assumed |
|  | *10% MSM* | *21% MSM* | *7% MSM* | *4% MSM* | *7% MSM* | *5% MSM* |  |
|  | *7% AGYW* | *16% AGYW* | *5% AGYW* | *3% AGYW* | *5% AGYW* | *4% AGYW* |  |
|  | *0.1% pregnant women* | *29% pregnant women* | *16% pregnant women* | *16% pregnant women* | *21% pregnant women* | *22% pregnant women* |  |
|  | *0.2% heterosexual men* | *0.5% heterosexual men* | *0.2% heterosexual men* | *0.2% heterosexual men* | *0.2% heterosexual men* | *0.2% heterosexual men* |  |
| *Coverage of injectable PrEP in 2030*  *(LEN or CAB)* | *0% all populations* | *0% all populations* | *40% FSW* | *65% FSW* | *30% FSW* | *55% FSW* | Assumed same or double initiation rates as TDF/FTC scale-up |
|  |  |  | *28% MSM* | *49% MSM* | *22% MSM* | *43% MSM* |  |
|  |  |  | *31% AGYW* | *51% AGYW* | *23% AGYW* | *43% AGYW* |  |
|  |  |  | *41% pregnant women* | *54% pregnant women* | *40% pregnant women* | *54% pregnant women* |  |
|  |  |  | *1% heterosexual men* | *3% heterosexual men* | *1% heterosexual men* | *2% heterosexual men* |  |
| **Duration of protection (+ tail coverage) in months (m)** | 3m (FSW, AGYW, heterosexual men) | 3m (FSW, AGYW, heterosexual men) | 6m (+6m tail) (FSW, AGYW, heterosexual men) | 12m (+6m tail protection) (FSW, AGYW, heterosexual men) | 4m (+3m tail) (FSW, AGYW, heterosexual men) | 8m (+3m tail) (FSW, AGYW, heterosexual men) | [2,3] |
|  | 6m (MSM) | 6m (MSM) | 12m (+6m tail) (MSM) | 24m (+6m tail) (MSM) | 8m (+3m tail) (MSM) | 16m (+3m tail) (MSM) |  |
| **Effectiveness in preventing HIV infection** | 65% (FSW, AGYW, heterosexual men) | 65% (FSW, AGYW, heterosexual men) | 99% (all populations) | 99% (all populations) | 95% (all populations) | 95% (all populations) | [2,4–9] |
|  | 85% (MSM) | 85% (MSM) |  |  |  |  |  |
| **Cost of provision of PrEP incl. drugs (per person initiated) **** |  |  |  |  |  |  |  |
| *TDF/FTC* | $57 (women, heterosexual men), $72 (MSM) | $57 (women, heterosexual men), $72 (MSM) | $57(women, heterosexual men), $72 (MSM) | $57(women, heterosexual men), $72 (MSM) | $57 (women, heterosexual men), $72 (MSM) | $57 (women, heterosexual men), $72 (MSM) | [10,11] |
| *LEN or CAB* | Not applicable | Not applicable | $60-$65 (women, heterosexual men), $85 (MSM) | $86-$91 (women, heterosexual men), $137 (MSM) | $146-$147 (women, heterosexual men), $226 (MSM) | $227-$228 (women, heterosexual men), $388 (MSM) | [10,11] |

*Abbreviations: AGYW = adolescent girls and young women, CAB = cabotegravir, FSW = female sex workers, HIV = human immunodeficiency virus, LEN = lenacapavir, m = months, MSM = men who have sex with men, PrEP = pre-exposure prophylaxis, TDF/FTC = tenofovir disoproxil fumarate/emtricitabine. **Full service cost presented includes costs for staff, HIV testing, laboratory testing, drugs, consumables and overheads. Assumed price for LEN (drug only) = $40 per person per year (PPPY) (4 injections/year) and $17 for a loading dose (1200mg LEN tablets); price for CAB (drug only) = $30/injection or approx. $180-210 per person per year (6-7 injections/year)

**Table B. Subpopulation prioritization: number of initiations assumed for each population**

All possible combinations of the below targets modelled where the total number of initiations ranged between 246,000 and 250,000 per year (totalling to 492,000-500,000 initiations over 2026/27). All populations are assumed to remain on LEN for 12 months. FSW and MSM were capped at a maximum coverage of 60% and 30% of HIV negative populations, respectively.

| **Adolescent girls and young women (AGYW)** | **Pregnant/breastfeeding women (PBFW)** | **Female sex workers (FSW)** | **Men who have sex with men (MSM)** |
| --- | --- | --- | --- |
| 0 | 0 | 0 | 0 |
| 12,500 | 12,500 | 4,500 | 9,360 |
| 25,000 | 25,000 | 9,000 | 18,720 |
| 37,500 | 37,500 | 13,500 | 28,080 |
| 50,000 | 50,000 | 18,000 | 37,440 |
| 62,500 | 62,500 | 22,500 | 46,800 |
| 75,000 | 75,000 | 27,000 | 56,160 |
| 87,500 | 87,500 | 36,000 | 65,520 |
| 100,000 | 100,000 | 45,000 | 74,880 |
| 112,500 | 112,500 |  | 84,240 |
| 125,000 | 125,000 |  | 93,600 |
| 137,500 | 137,500 |  |  |
| 150,000 | 150,000 |  |  |
| 162,500 | 162,500 |  |  |
| 175,000 | 175,000 |  |  |
| 187,500 | 187,500 |  |  |
| 200,000 | 200,000 |  |  |
| 212,500 | 212,500 |  |  |
| 225,000 | 225,000 |  |  |
| 237,500 | 237,500 |  |  |
| 250,000 | 250,000 |  |  |

**Table C. Probability distributions used for parameters varied in the probabilistic sensitivity analysis**

| **Variable** | **Population** | **Distribution** | **Mean,**  **standard**  **deviation** | **Source/ assumption** |
| --- | --- | --- | --- | --- |
| Reduction in condom use while on PrEP | All populations | Beta (0.80, 7.20) | 0.10, 0.10 | [1] |
| TDF/FTC effectiveness | Women and heterosexual men | Beta (14.14,7.61) | 0.65, 0.10 |  |
|  | Pregnant women | Beta (10.69,13.06) | 0.65, 0.10 |  |
|  | MSM | Beta (9.99, 1.76) | 0.85, 0.10 |  |
| CAB effectiveness | All populations | Beta (111.86, 5.89) | 0.95, 0.05 | [3] |
| CAB tail protection (months) | All populations | Gamma (2.25,0.75) | 3.0, 2.0 |  |
| LEN effectiveness | All populations | Beta (69.11, 1.12) | 0.984, 0.014 | [2] |
| LEN tail protection (months) | All populations | Gamma (2.25,0.38) | 6.0, 4.0 |  |
| Cost of LEN 6-12-month duration (including drugs) | Women (6 months) | Uniform ($42, $87) | $65, $13 | Service delivery costs were varied by a 50% decrease and increase on midpoint estimate, while maintaining the same cost of drugs |
|  | Heterosexual men (6 months) | Uniform ($40, $80) | $60, $12 |  |
|  | MSM (12 months) | Uniform ($63, $108) | $85, $13 |  |
| Cost of LEN 12-24-month duration (including drugs) | Women (12 months) | Uniform ($66, $117) | $91, $15 |  |
|  | Heterosexual men (12 months) | Uniform ($63, $109) | $86, $13 |  |
|  | MSM (24 months) | Uniform ($109, $166) | $137, $17 |  |
| Cost of CAB 4-8-month duration (including drugs) | Women (4 months) | Uniform ($124, $170) | $147, $13 |  |
|  | Heterosexual men (4 months) | Uniform ($123, $168) | $146, $13 |  |
|  | MSM (8 months) | Uniform ($197, $255) | $226, $17 |  |
| Cost of CAB 8-16-month duration (including drugs) | Women (8 months) | Uniform ($198, $259) | $228, $18 |  |
|  | Heterosexual men (8 months) | Uniform ($197, $257) | $227, $17 |  |
|  | MSM (16 months) | Uniform ($345, $432) | $388, $25 |  |

*Abbreviations: CAB = cabotegravir, LEN = lenacapavir, MSM = men who have sex with men, PrEP = pre-exposure prophylaxis, TDF/FTC = tenofovir disoproxil fumarate/emtricitabine

We ran 1,000 Monte Carlo simulations, sampling the variables from their distributions and shape parameters in each run. Table S2 summarizes the distributions from which the PrEP parameters are sampled in the uncertainty analysis. In addition to these, we sample from the posterior distributions generated in the calibration of the Thembisa model to various HIV data sources, so that the uncertainty ranges also reflect uncertainty regarding epidemiological processes. These include the uncertainty in HIV incidence trends, the relative HIV incidence across age/sex/risk groups, rates of HIV disease progression and mortality, and rates of HIV testing and ART linkage. More details regarding the specific parameters and their posterior distributions are included in the Thembisa 4.8 report^[[1]](#footnote-1)^: Table 8.1 of the report summarizes the posterior distributions for adult HIV transmission and disease progression, Table B9 summarizes the posterior distributions for the HIV testing parameters, Table E6 summarizes the posterior distributions for the paediatric HIV parameters, and section 7.4 summarizes the distributions used to represent uncertainty in the future ART coverage, medical male circumcision uptake and condom use.

**Figure A. (A) Number of people needed to initiate, and (B) doses required, to avert one HIV infection, by scenario.** *Acronyms: CAB=cabotegravir, LEN=lenacapavir, TDF/FTC = tenofovir disoproxil fumarate/emtricitabine*

**A**

**B**

**
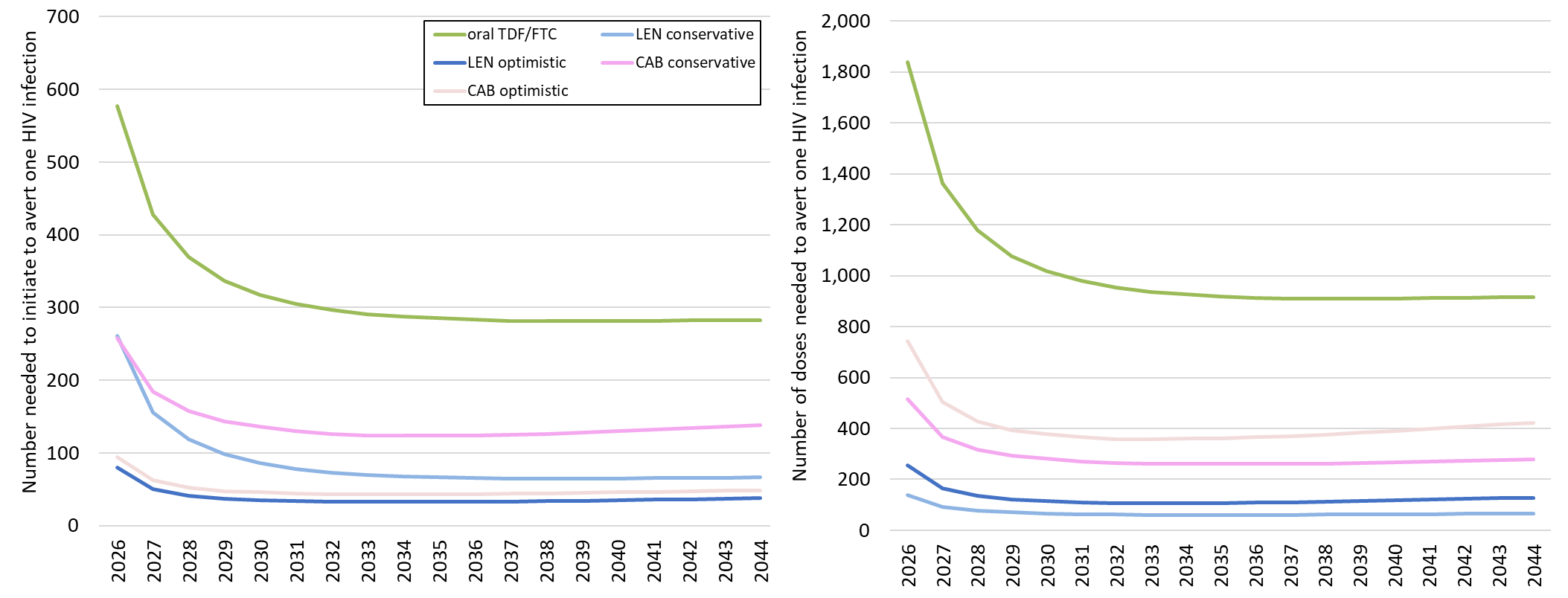
**

**Figure B. Relative impact and cost-effectiveness comparing LEN and TDF/FTC scale up in a one-way sensitivity analysis varying key risk, uptake and cost parameters.** *Results depict the ratio of ICER/LYS in (A) conservative and (B) optimistic scenarios, and ratio of life years saved in LEN vs TDF/FTC scale-up in (C) conservative and (D) optimistic scenarios. Acronyms: LEN=lenacapavir, TDF/FTC = tenofovir disoproxil fumarate/emtricitabine, ICER = incremental cost effectiveness ratio, LYS = life years saved, PPPY = per person per year.***
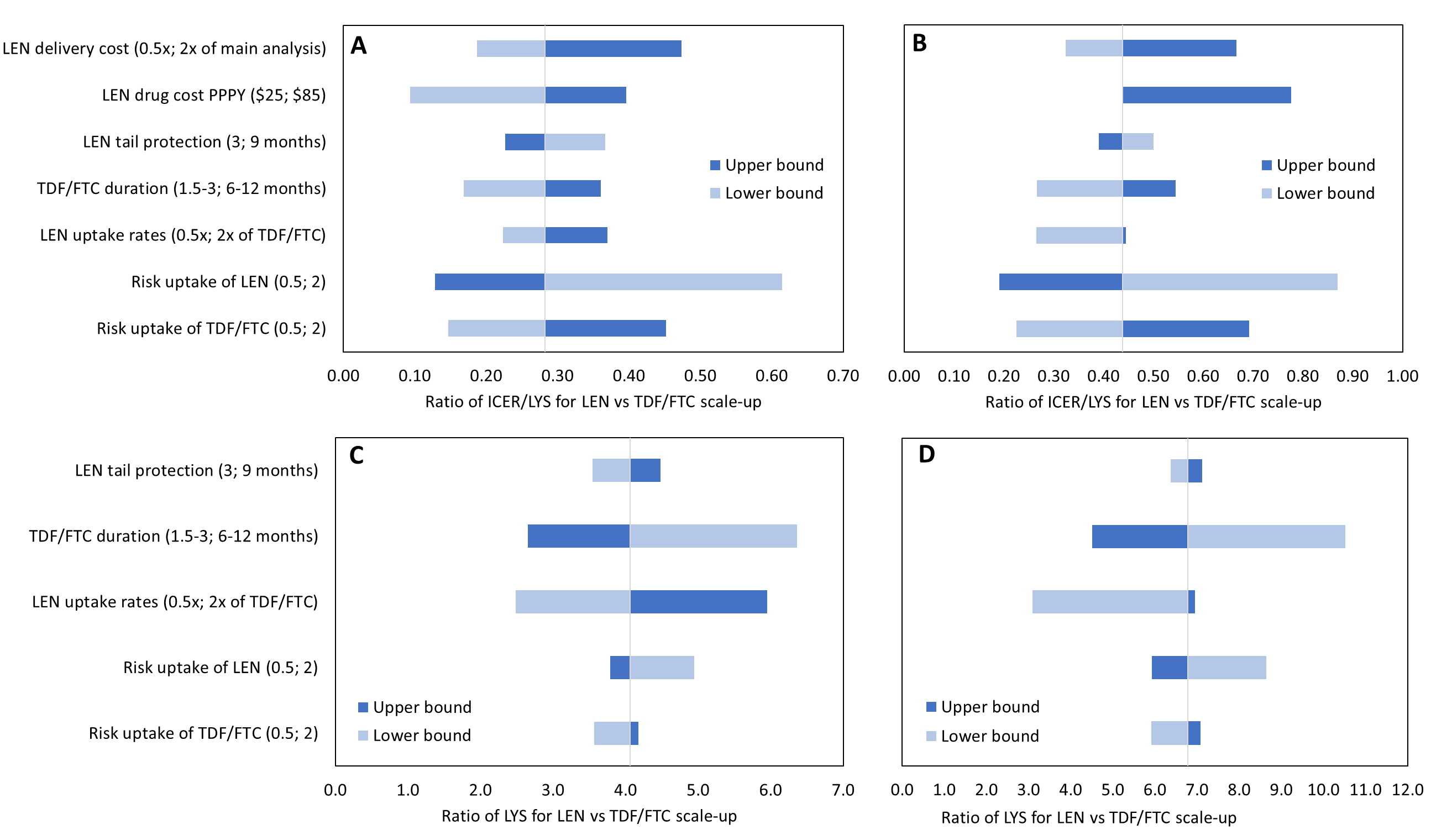
**

**Figure C. Relative impact and cost-effectiveness comparing CAB and TDF/FTC scale up in a sensitivity analysis varying key risk, uptake and cost parameters.** *Results depict the ratio of ICER/LYS in (A) conservative and (B) optimistic scenarios, and ratio of life years saved in CAB vs TDF/FTC scale-up in (C) conservative and (D) optimistic scenarios. Acronyms: CAB=cabotegravir, TDF/FTC = tenofovir disoproxil fumarate/emtricitabine, ICER = incremental cost effectiveness ratio, LYS = life years saved**, PPPY = per person per year.*

*
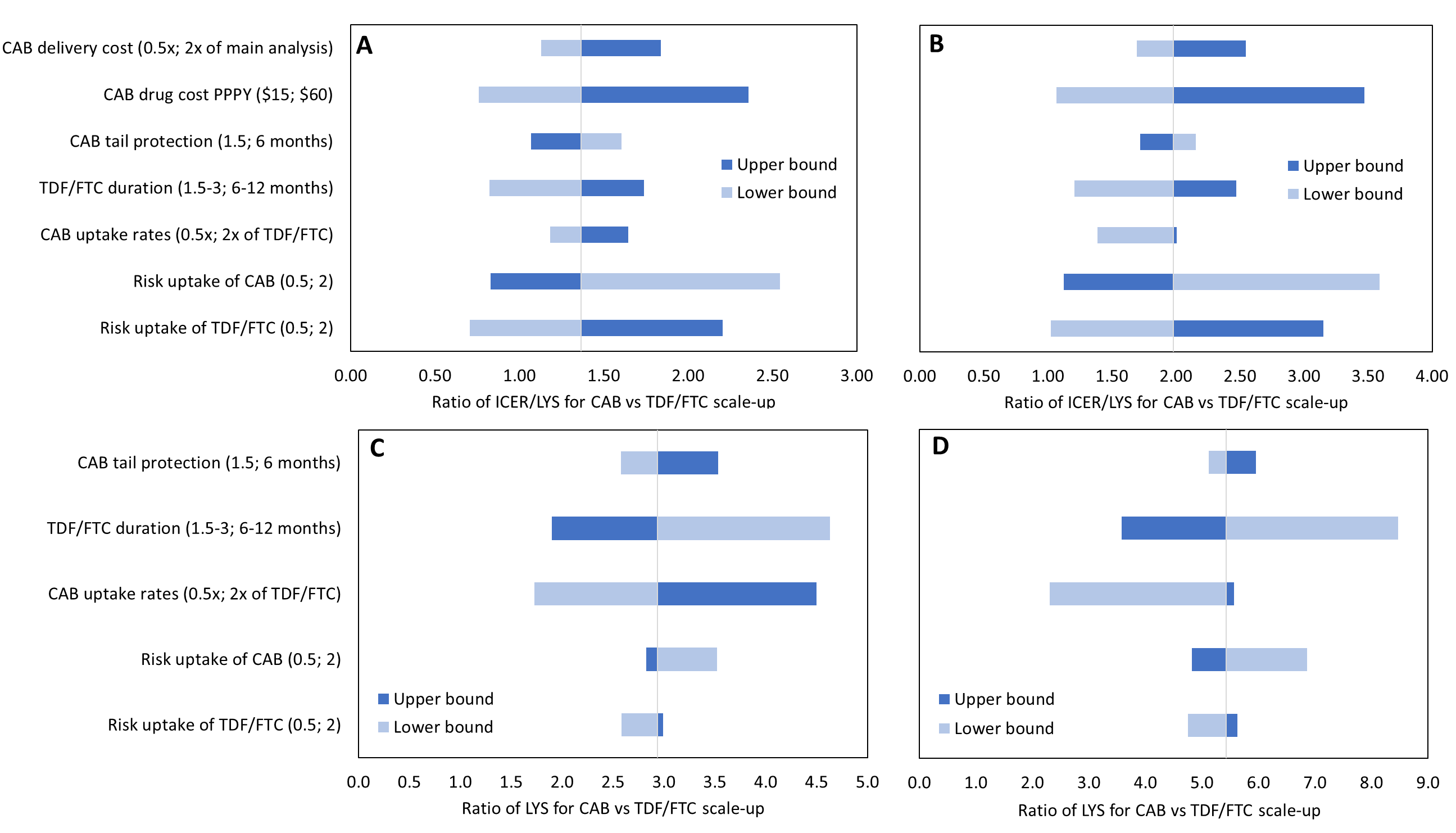
*

**Figure D. One-way sensitivity analysis of five-year impact of different combinations of subpopulation uptake when (A) reducing, and (B) increasing the rate of high-risk uptake of lenacapavir (LEN) within the general population.** *Total LEN is constrained to (~500,000 person-years on LEN) over 2026-2027. Each of the 490 different combinations of LEN distribution are represented by vertical bars, with all results sorted by descending order of impact, therefore the leftmost combinations represent the highest impact strategies.*


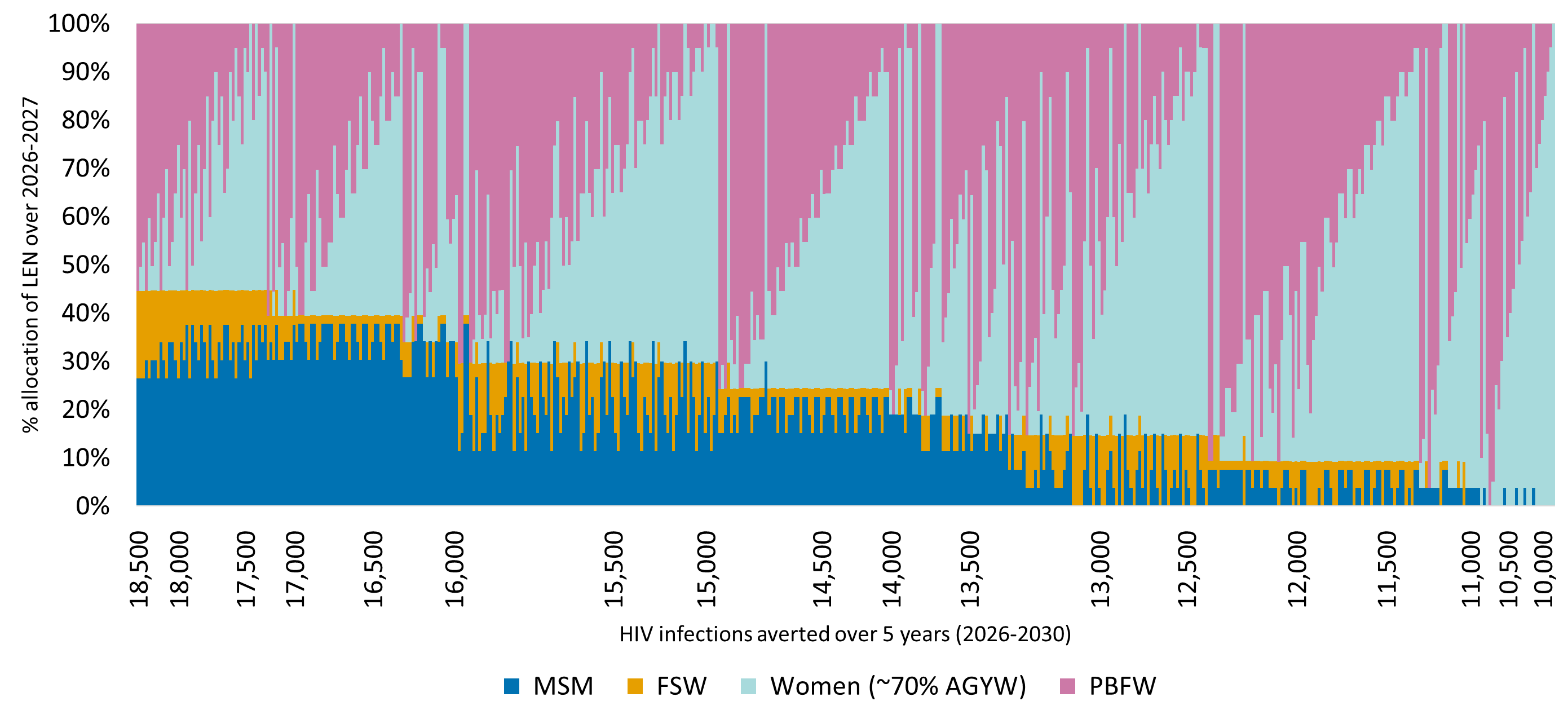

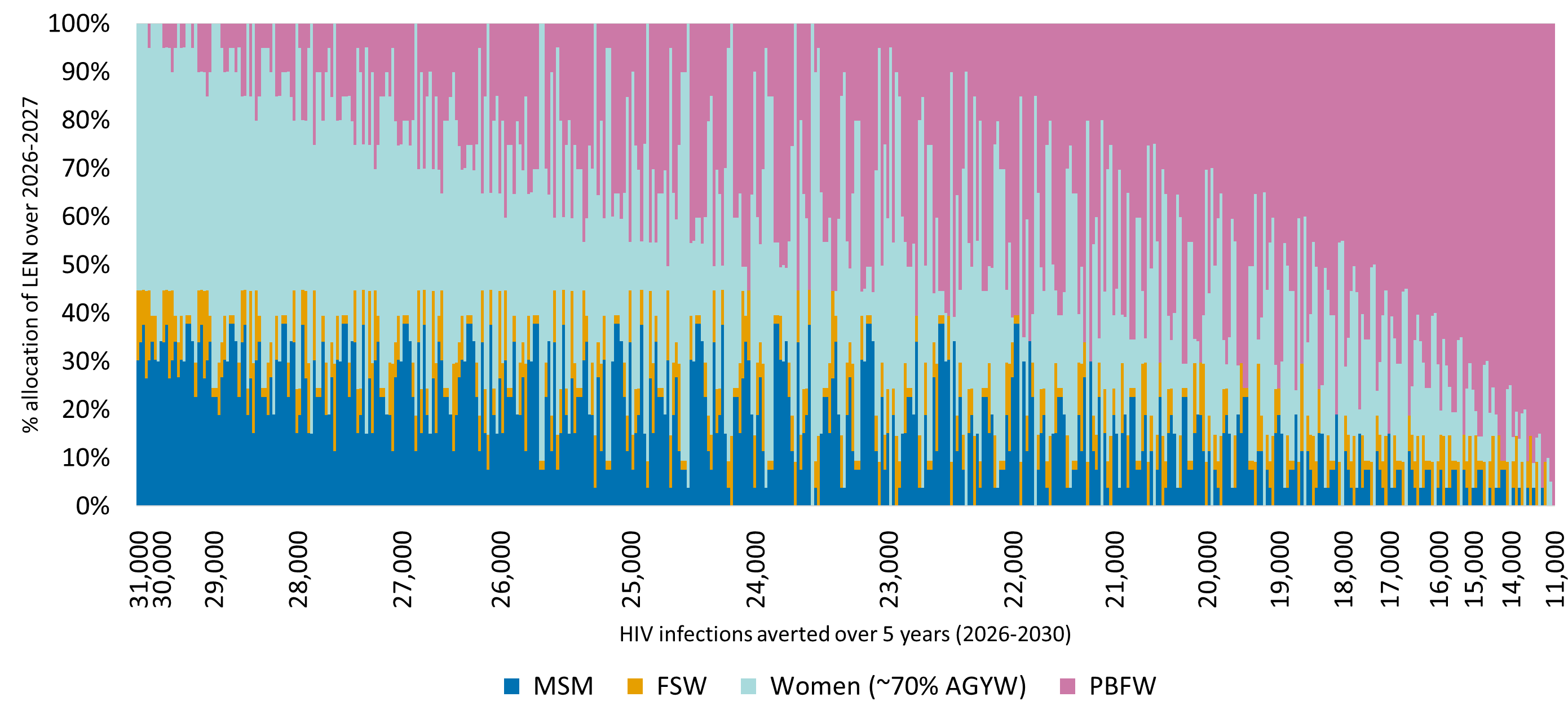


**A**

**B**

**Figure E. Probabilistic sensitivity analysis results comparing the incremental cost per life year saved over 2026-2045 for TDF/FTC scale-up to that of the LEN (A) conservative and (B) optimistic scale-up scenarios, and the CAB (C) conservative, and (D) optimistic scale-up scenarios**

*Each dot represents a single simulation of a total of 1000 simulations, the diagonal line represents equal cost-effectiveness between TDF/FTC and LEN/CAB. The price of LEN was $40 per person per year (PPPY) (4 injections) and $17 for the loading dose tablets; price of CAB is the current manufacturer offered price of $180-210 PPPY (6-7 injections). Acronyms: CAB=cabotegravir, LEN=lenacapavir, TDF/FTC = tenofovir disoproxil fumarate/emtricitabine, USD = United States Dollars.*


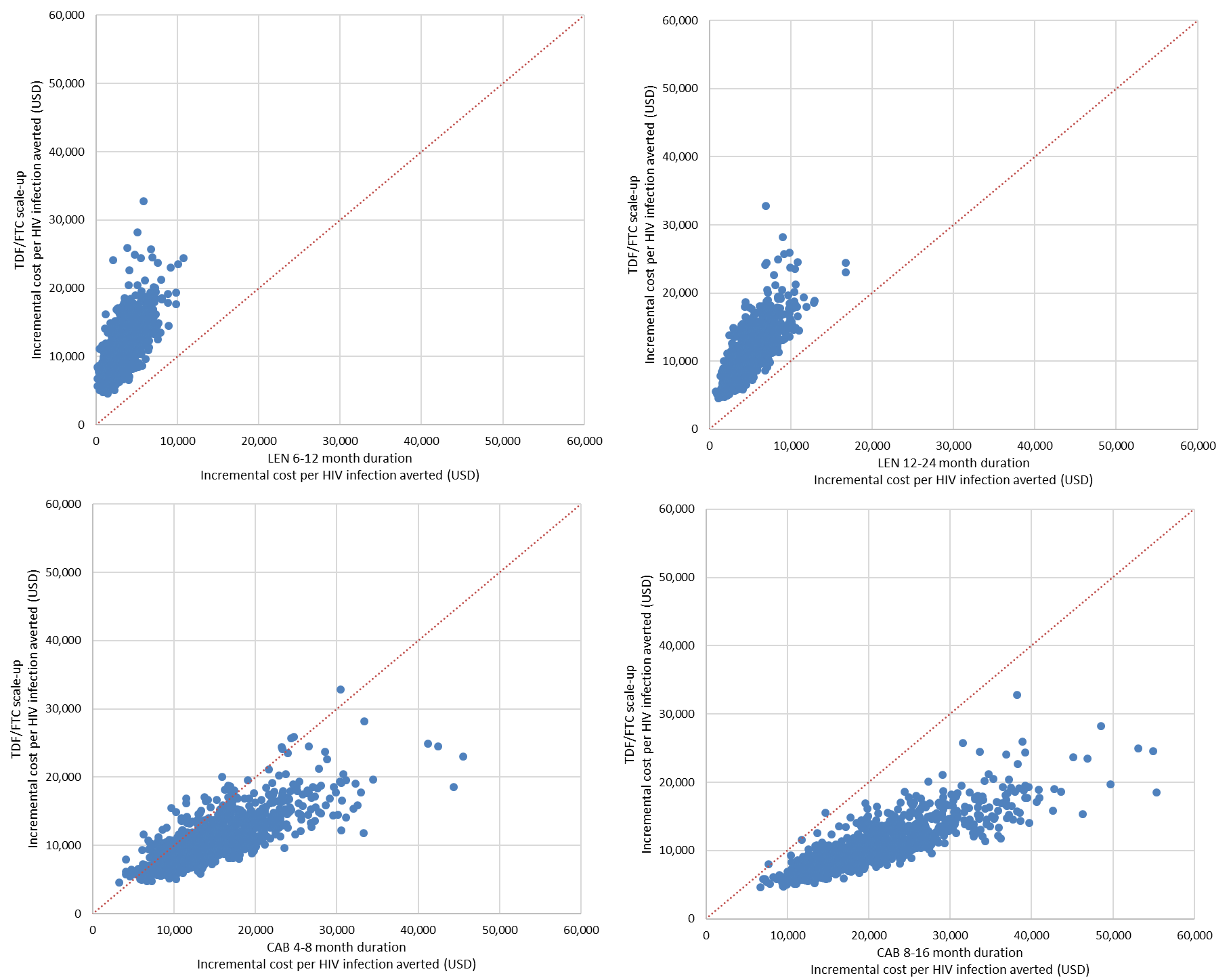


**B**

**A**

**D**

**C**

**Table D. Cost breakdown of each PrEP modality by population and duration**

| **Cost of LEN provision** | | | | | | |
| --- | --- | --- | --- | --- | --- | --- |
| **Cost category** | **Women** | | **Heterosexual men** | | **MSM** | |
|  | **6 months duration** | **12 months duration** | **6 months duration** | **12 months duration** | **12 months duration** | **24 months duration** |
| Drugs | $37.00 | $57.00 | $37.00 | $57.00 | $57.00 | $97.00 |
| Staff | $20.02 | $23.63 | $15.80 | $19.40 | $19.63 | $26.87 |
| HIV tests | $3.11 | $3.81 | $3.14 | $3.83 | $2.41 | $3.81 |
| Consumables | $0.47 | $0.85 | $0.20 | $0.30 | $0.33 | $0.53 |
| Overheads | $4.39 | $6.12 | $4.08 | $5.78 | $5.70 | $9.12 |
| **Total** | **$64.99** | **$91.40** | **$60.22** | **$86.32** | **$85.07** | **$137.33** |
| **Cost of TDF/FTC provision** | | | | | | |
| **Cost category** | **Women (3 months duration)** | | **Heterosexual men (3 months duration)** | | **MSM (6 months duration)** | |
| Drugs | $13.52 | | $13.52 | | $23.66 | |
| Staff | $20.70 | | $20.02 | | $23.73 | |
| Laboratory testing | $13.98 | | $14.75 | | $14.57 | |
| Consumables | $0.96 | | $0.55 | | $0.96 | |
| Overheads | $8.50 | | $8.47 | | $9.46 | |
| **Total** | **$57.65** | | **$57.32** | | **$72.38** | |
| **Cost of CAB provision** | | | | | | |
| **Cost category** | **Women** | | **Heterosexual men** | | **MSM** | |
|  | **4 months duration** | **8 months duration** | **4 months duration** | **8 months duration** | **8 months duration** | **16 months duration** |
| Drugs | $100.54 | $167.56 | $100.54 | $167.56 | $167.56 | $301.61 |
| Staff | $27.24 | $34.47 | $27.25 | $34.46 | $34.73 | $49.21 |
| Laboratory testing | $7.80 | $9.19 | $7.82 | $9.20 | $7.80 | $10.60 |
| Consumables | $1.30 | $2.11 | $0.48 | $0.73 | $0.76 | $1.27 |
| Overheads | $9.73 | $15.08 | $9.67 | $14.98 | $14.91 | $25.53 |
| **Total** | **$146.61** | **$228.41** | **$145.76** | **$226.93** | **$225.77** | **$388.23** |

Abbreviations: CAB = cabotegravir, LEN = lenacapavir, MSM = men who have sex with men, TDF/FTC = tenofovir disoproxil fumarate/emtricitabine

**Table E. Budget impact analysis of LEN scale-up**

*Two budget impact analyses are presented: (1) at the currently negotiated price of $40 PPPY for injections and $17 for the loading dose, and (2) a lower bound estimated price of $25 PPPY. All costs include service provision and drugs, and are presented in 2025 USD.*

| **Conservative lenacapavir scale-up, same initiation rates as TDF/FTC, 6-12-month duration** | | | | | |
| --- | --- | --- | --- | --- | --- |
|  | **2025/26** | **2026/27** | **2027/28** | **2028/29** | **2029/30** |
| Clients initiated (millions) | 0.59 | 0.78 | 0.98 | 1.17 | 1.39 |
| Doses required (millions) | 0.61 | 0.82 | 1.03 | 1.24 | 1.47 |
| **Total cost in USD, millions (% increase over current HIV programme cost)** | | | | | |
| Currently negotiated generic price of $40 PPPY ^a^ | 38 (2%) | 51 (3%) | 64 (3%) | 77 (4%) | 91 (5%) |
| Lower bound estimate of $25 PPPY ^b^ | 23 (1%) | 30 (2%) | 38 (2%) | 46 (2%) | 54 (3%) |
| **Optimistic lenacapavir scale-up, higher initiation rates than TDF/FTC, 12-24-month duration** | | | | | |
|  | **2025/26** | **2026/27** | **2027/28** | **2028/29** | **2029/30** |
| Clients initiated (millions) | 0.91 | 1.23 | 1.53 | 1.82 | 2.13 |
| Doses required (millions) | 1.88 | 2.59 | 3.23 | 3.84 | 4.50 |
| **Total cost in USD, millions (% increase over current HIV programme cost)** | | | | | |
| Currently negotiated generic price of $40 PPPY ^a^ | 84 (4%) | 115 (6%) | 143 (7%) | 169 (9%) | 198 (10%) |
| Lower bound estimate of $25 PPPY ^b^ | 52 (3%) | 71 (4%) | 89 (5%) | 105 (5%) | 123 (6%) |

^a^ Negotiated drug cost: $40 for injections PPPY (2 x 927mg); $17 per 4x300mg loading dose tablets. Source: Unitaid, CHAI, and Wits RHI enter into a landmark agreement with Dr. Reddy’s to make HIV prevention tool lenacapavir affordable in low- and middle income countries (LMIC) - Unitaid. Available: https://unitaid.org/news-blog/lenacapavir-for-hiv-prevention. This results in a cost of provision (including drugs) per person initiated of $60-$65 (average duration of 6 months), $85-$91 (12-month duration), $137 (24-month duration).

^b^ Estimated by Fairhead et al., Open Forum Infect Dis. 2026;13:ofaf695.004.

Abbreviations: HIV = human immunodeficiency virus, PPPY = per person per year, TDF/FTC = tenofovir disoproxil fumarate/emtricitabine, USD = United States dollars

**Table F. Selected distribution scenarios of LEN allocation of ~500,000 person-years on LEN over 2026-27 for maximum impact on HIV infections**

|  |  | **% of LEN allocation distributed to subpopulations**  *(number of person-years on LEN)* | | | |
| --- | --- | --- | --- | --- | --- |
| **Scenario** | **HIV infections averted**  **over 2026-2030** | **FSW** | **MSM** | **Women**  **(~70% AGYW)** | **PBFW** |
| *NDOH planned scenario* | **19,400** | 9.1% (45,000) | 15.1% (74,880) | 55.6% (275,000) | 20.2% (100,000) |
| **High impact scenarios** |  |  |  |  |  |
| *Overall highest impact* | **23,100** | 14.5% (72,000) | 30.1% (149,760) | 55.4% (275,000) | 0.0% (0) |
| **Highest impact at set distribution to PBFW** | | | | | |
| *~10% to PBFW* | **22,700** | 14.5% (72,000) | 30.1% (149,760) | 45.3% (225,000) | 10.1% (50,000) |
| *~20% to PBFW* | **22,200** | 7.2% (36,000) | 37.6% (187,200) | 35.1% (175,000) | 20.1% (100,000) |
| *~30% to PBFW* | **21,800** | 14.5% (72,000) | 30.1% (149,760) | 25.2% (125,000) | 30.2% (150,000) |
| *~40% to PBFW* | **21,400** | 14.5% (72,000) | 30.1% (149,760) | 15.1% (75,000) | 40.3% (200,000) |
| *~50% to PBFW* | **20,800** | 14.5% (72,000) | 30.1% (149,760) | 5.0% (25,000) | 50.3% (250,000) |

Abbreviations: AGYW = adolescent girls and young women, FSW = female sex workers, HIV = human immunodeficiency virus, LEN = lenacapavir, MSM = men who have sex with men, NDOH = National Department of Health, PBFW = pregnant and breastfeeding women

**Table G. Sensitivity of high-risk uptake and service delivery costs for FSW and MSM on the cost-effectiveness of sub-population uptake of LEN, compared to baseline, over 20 years (2026-2045)**

|  | **Incremental cost effectiveness ratio (ICER) per life year saved (LYS) in 2025 USD** | | | | |
| --- | --- | --- | --- | --- | --- |
| **Scenario** | **Main analysis** | **Reduced high risk uptake*** | **Increased high risk uptake*** | **Service delivery costs for FSW, MSM doubled** | **Service delivery costs for FSW, MSM tripled** |
| FSW | Cost-saving | Cost-saving | Cost-saving | 566 | 1,154 |
| FSW and MSM | 252 | 393 | 77 | 705 | 1,158 |
| MSM | 331 | 524 | 98 | 741 | 1,151 |
| FSW, MSM and pregnant women | 1,102 | 1,203 | 951 | 1,379 | 1,656 |
| FSW and AGYW | 1,305 | 1,802 | 714 | 1,435 | 1,565 |
| AGYW | 1,577 | 2,130 | 892 | 1,607 | 1,637 |
| PBFW | 2,220 | 2,220 | 2,220 | 2,243 | 2,266 |
| Heterosexual men | 7,700 | 9,649 | 4,525 | 7,796 | 7,891 |

*The degree of risk stratification in LEN uptake was varied by applying a power of 0.5 (flatter risk gradient, low-risk uptake closer to high-risk) and 2.0 (steeper risk gradient, greater relative difference between high- and low-risk uptake) to the relative uptake rates.

Abbreviations: AGYW = adolescent girls and young women, FSW = female sex workers, ICER = incremental cost-effectiveness ratio, LEN = lenacapavir, LYS = life years saved, MSM = men who have sex with men, PBFW = pregnant and breastfeeding women, USD = United States dollars

**Table H. Median, lower and upper uncertainty bounds around the impact and cost-effectiveness of TDF/FTC, LEN and CAB over a 20-year time horizon (2026-2045); based on 1,000 Monte Carlo simulations in a probabilistic sensitivity analysis*.** *Numbers represent median estimate across simulations and in brackets 2.5^th^ and 97.5^th^ percentiles.*

|  | **Total Cost of the HIV programme** | | **Incremental cost effectiveness** | | | **New HIV infections** | | | **Life years lost due to AIDS** | | |
| --- | --- | --- | --- | --- | --- | --- | --- | --- | --- | --- | --- |
| **Scenario** | **Cost**  **(billions, USD)** | **Incremental cost over baseline, %** | | **Cost per infection averted (USD)** | **Cost per life year saved (USD)** | | **Number (millions)** | **% averted over baseline** | | **Number (millions)** | **% saved over baseline** |
| **Baseline** | 39.77 (37.02-43.27) | - | | - | - | | 2.61 (1.78-3.83) | - | | 19.28 (16.52-22.96) | - |
| **Oral TDF/FTC scale up** | 40.97 (38.42-44.29) | 3.0% (2.4%-3.8%) | | 10,336 (5,863-19,157) | 8,445 (4,958-15,023) | | 2.50 (1.71-3.67) | 4.4% (3.4%-5.5%) | | 19.14 (16.42-22.78) | 0.7% (0.5%-0.9%) |
| **Lenacapavir** |  |  | |  |  | |  |  | |  |  |
| Conservative | 41.22 (38.60-44.40) | 3.6% (2.6%-4.3%) | | 2,930 (803-7,142) | 2,539 (669-5,904) | | 2.12 (1.48-3.08) | 18.1% (13.8%-24.1%) | | 18.70 (16.18-22.18) | 2.8% (1.9%-4.1%) |
| Optimistic | 43.45 (40.86-46.29) | 9.2% (7.0%-10.4%) | | 4,481 (1,761-9,897) | 3,803 (1,530-8,072) | | 1.81 (1.29-2.58) | 30.4% (25.5%-35.7%) | | 18.32 (15.91-21.61) | 4.9% (3.5%-6.3%) |
| **Cabotegravir** |  |  | |  |  | |  |  | |  |  |
| Conservative | 44.63 (42.27-47.49) | 12.2% (9.8%-14.2%) | | 14,010 (6,516-28,606) | 12,050 (5,700-23,394) | | 2.26 (1.56-3.27) | 13.2% (10.0%-18.8%) | | 18.86 (16.27-22.43) | 2.1% (1.4%-3.1%) |
| Optimistic | 52.83 (50.44-55.35) | 32.8% (27.9%-36.3%) | | 20,272 (10,455-38,496) | 17,154 (9,249-31,212) | | 1.96 (1.37-2.81) | 24.6% (20.1%-30.0%) | | 18.50 (16.02-21.92) | 3.9% (2.8%-5.2%) |

*We sampled for 58 parameters for the sensitivity analysis, including key PrEP-related parameters (PrEP effectiveness, reduction in condom use while on PrEP, tail protection duration) and the cost of service provision of LEN and CAB, and sampled from pre-determined distributions for each of the 1,000 model runs (see Table S1). Abbreviations: AIDS = Acquired Immunodeficiency Syndrome, CAB = cabotegravir, HIV = human immunodeficiency virus, LEN = lenacapavir, TDF/FTC = tenofovir/emtricitabine, USD = United States Dollars.

**References**

1. Johnson L, Dorrington R. Thembisa version 4.8: a model for evaluating the impact of HIV/AIDS in South Africa. 2025. Available: https://thembisa.org/content/downloadPage/Thembisa4_8report

2. Bekker L-G, Das M, Abdool Karim Q, Ahmed K, Batting J, Brumskine W, et al. Twice-Yearly Lenacapavir or Daily F/TAF for HIV Prevention in Cisgender Women. N Engl J Med. 2024. doi:10.1056/NEJMoa2407001

3. Landovitz RJ, Li S, Eron JJ, Grinsztejn B, Dawood H, Liu AY, et al. Tail-phase safety, tolerability, and pharmacokinetics of long-acting injectable cabotegravir in HIV-uninfected adults: a secondary analysis of the HPTN 077 trial. Lancet HIV. 2020;7: e472–e481. doi:10.1016/S2352-3018(20)30106-5

4. Kelley CF, Acevedo-Quiñones M, Agwu AL, Avihingsanon A, Benson P, Blumenthal J, et al. Twice-Yearly Lenacapavir for HIV Prevention in Men and Gender-Diverse Persons. N Engl J Med. 2025;392: 1261–1276. doi:10.1056/NEJMoa2411858

5. Molina J-M, Capitant C, Spire B, Pialoux G, Cotte L, Charreau I, et al. On-Demand Preexposure Prophylaxis in Men at High Risk for HIV-1 Infection. N Engl J Med. 2015;373: 2237–2246. doi:10.1056/NEJMoa1506273

6. McCormack S, Dunn DT, Desai M, Dolling DI, Gafos M, Gilson R, et al. Pre-exposure prophylaxis to prevent the acquisition of HIV-1 infection (PROUD): effectiveness results from the pilot phase of a pragmatic open-label randomised trial. The Lancet. 2016;387: 53–60. doi:10.1016/S0140-6736(15)00056-2

7. Bekker L-G, Roux S, Sebastien E, Yola N, Amico KR, Hughes JP, et al. Daily and non-daily pre-exposure prophylaxis in African women (HPTN 067/ADAPT Cape Town Trial): a randomised, open-label, phase 2 trial. Lancet HIV. 2018;5: e68–e78. doi:10.1016/S2352-3018(17)30156-X

8. Delany-Moretlwe S, Hughes JP, Bock P, Ouma SG, Hunidzarira P, Kalonji D, et al. Cabotegravir for the prevention of HIV-1 in women: results from HPTN 084, a phase 3, randomised clinical trial. The Lancet. 2022;399: 1779–1789. doi:10.1016/S0140-6736(22)00538-4

9. Landovitz RJ, Donnell D, Clement ME, Hanscom B, Cottle L, Coelho L, et al. Cabotegravir for HIV Prevention in Cisgender Men and Transgender Women. N Engl J Med. 2021;385: 595–608. doi:10.1056/NEJMoa2101016

10. Jamieson L, Johnson LF, Nichols BE, Delany-Moretlwe S, Hosseinipour MC, Russell C, et al. Relative cost-effectiveness of long-acting injectable cabotegravir versus oral pre-exposure prophylaxis in South Africa based on the HPTN 083 and HPTN 084 trials: a modelled economic evaluation and threshold analysis. The Lancet HIV. 2022;9: e857–e867. doi:10.1016/S2352-3018(22)00251-X

11. Jamieson L, Gomez GB, Rebe K, Brown B, Subedar H, Jenkins S, et al. The impact of self-selection based on HIV risk on the cost-effectiveness of preexposure prophylaxis in South Africa. AIDS. 2020;34: 883–891. doi:10.1097/QAD.0000000000002486

1. Johnson L, Dorrington R. Thembisa version 4.8: a model for evaluating the impact of HIV/AIDS in South Africa. 2025 https://thembisa.org/content/downloadPage/Thembisa4_8report (accessed April 14, 2025) [↑](#footnote-ref-1)
