# Supplementary material for: Optimising scale-up of injectable lenacapavir for HIV pre-exposure prophylaxis in South Africa: A modelling study and economic evaluation
Source: PLoS Med. 2026 Jul 21;23(7):e1004882. doi: 10.1371/journal.pmed.1004882 (PMC13421763; doi:10.1371/journal.pmed.1004882)
Supplement: Attachment — Submitted filename: Response to reviewers 19 Dec 2025.pdf [file pmed.1004882.s005.pdf]

Health Economics and Epidemiology Research Office

32 Princess of Wales Terrace, Sunnyside Office Park, Building C,First Floor.
Parktown, Johannesburg, 2193, South Africa
Tel +27 (0)10 0017930

A division of the Wits Health Consortium (Pty) Ltd, a wholly owned subsidiary of the University of the Witwatersrand.

19 December 2025

Response to reviewers

Below details our responses to the initial feedback to our manuscript entitled **"Taking injectable PrEP to scale: optimising the value of lenacapavir for South Africa's HIV response"**,

Feedback	Response
Please also provide some additional brief explanatory information in the Background section of the Abstract for the general reader, and pertaining to why this analysis is important at this time, in this population.	Thank you. We have updated the abstract to include more justification of the importance of this work.
Please remove use of vast (X2) from the abstract and use more objective language.	We have removed these words in the Abstract.
Please note the study limitations in the last sentence of the Methods & Findings section of the Abstract.	We have included a short sentence highlighting the limitations of this work in the Abstract (lines 54-56)
Please explain what Thembisa v4.8 is for the general reader.	We have changed the first sentence in the Methods and Findings to briefly describe the model.
Please provide greater clarity in the Discussion on potential discrepancies in cost estimates with related studies that you cite.	We have included more on this in the Discussion (see lines 340-347) as well as included more in the Results describing the costs (lines 250-254) and a more detailed cost breakdown in the Supplementary appendix (Table S2).
Please provide some additional clarifying information to the sentence beginning on line 43 of the Abstract.	We have updated the sentence to clarify this analysis (starting in line 47)
Please correct phrasing on line 51.	Thank you. This has been corrected.
Line 27--please qualify impact (impact on what?).	We have clarified this is this epidemiological impact on HIV infections and life years lost (see lines 30-32).

Sincerely,

Lise Jamieson, PhD
Health Economics and Epidemiology Research Office (HE²RO)
University of the Witwatersrand, Johannesburg, South Africa
Email: ljamieson@heroza.org
