# Supplementary material for: Optimising scale-up of injectable lenacapavir for HIV pre-exposure prophylaxis in South Africa: A modelling study and economic evaluation
Source: PLoS Med. 2026 Jul 21;23(7):e1004882. doi: 10.1371/journal.pmed.1004882 (PMC13421763; doi:10.1371/journal.pmed.1004882)
Supplement: Attachment — Submitted filename: Responses to reviewers and editorial requests FINAL 05May26.pdf [file pmed.1004882.s006.pdf]

Health Economics and Epidemiology Research Office

32 Princess of Wales Terrace, Sunnyside Office Park, Building C,First Floor.
Parktown, Johannesburg, 2193, South Africa
Tel +27 (0)10 0017930

A division of the Wits Health Consortium (Pty) Ltd, a wholly owned subsidiary of the University of the Witwatersrand.

05 May 2026

RE: Response to reviewers

The below table contains our detailed responses to each of the reviewers.

Comment	Response
**Reviewer #1**	
General comment: This is a timely, policy-relevant modelling analysis addressing an immediate implementation decision- how South Africa should deploy long-acting injectable lenacapavir for PrEP under constrained resources and reduced external funding. The work is clearly motivated, uses an established national HIV model, and provides decision-facing outputs on impact, cost-effectiveness, and prioritisation of limited initial supply. My comments focus on the issues that most affect the validity and policy interpretation of the results.	
1) Targeting performance is the dominant structural uncertainty and needs explicit stress-testing<br><br>Your conclusions about LEN's efficiency and the prioritisation of early supply rely heavily on how well uptake is concentrated among those at highest acquisition risk. This is not a minor uncertainty, even modest leakage of uptake towards lower-risk individuals within eligible groups can materially reduce infections averted per initiation and weaken cost-effectiveness.<br><br>At present, the analysis implicitly assumes a strong relationship between risk and uptake within groups. In real-world South African PrEP delivery, targeting is imperfect, and scale-up typically expands beyond the highest-incidence individuals first reached.<br><br>Perhaps include at least one structural sensitivity analysis where uptake is less well targeted within groups (e.g., a weaker correlation between uptake and underlying incidence, or a defined fraction of initiations occurring among lower-risk individuals). Report whether this changes (i) the ranking of prioritised groups for early LEN, and (ii) the relative advantage of LEN vs CAB and oral PrEP.	Thank you for pointing this out. Indeed, the efficiency of LEN or CAB depends on the level of risk of those who choose to initiate it, and how it relatively compares to oral PrEP.<br><br>We have now included one-way sensitivity analyses in the paper, which assess the impact of several parameters and evaluate the impact on the relative cost-effectiveness of injectable PrEP options vs TDF/FTC scale up (represented by the ratio of incremental cost effectiveness ratio per life year saved where a value <1 means injectable PrEP is more cost-effective than TDF/FTC, and a value of >1 means that TDF/FTC is more cost-effective injectable PrEP). We took a similar approach with a ratio of life years saved. In addition to this we have also varied the impact of differential high risk uptake in LEN vs TDF/FTC on the cost-effectiveness of scaling up LEN between different subgroups nationally, and whether the early LEN roll-out prioritization is affected.<br><br>We added the following in Methods, lines 258-263:<br>*"We conducted a one-way sensitivity analysis within our main analysis to evaluate the impact of key PrEP parameters, including uptake, differential risk targeting across PrEP options,*

Comment	Response
This would substantially strengthen the policy reliability of the prioritisation conclusions.	*TDF/FTC duration, LEN/CAB tail protection, and costs. We also conducted sensitivity analysis to examine whether improved or poorer risk targeting, or higher service delivery costs for FSW or MSM, affected impact of different subpopulation distributions in Phase 1, or the relative cost-effectiveness of LEN scale-up in Phase 2 of our third analysis."*<br><br>We added a tornado diagram in the supplementary appendix (Figures S2 and S3), and updated the Results section, lines 343-356: *"One-way sensitivity analyses showed that the cost-effectiveness and impact of LEN, relative to TDF/FTC scale-up, were robust (Figure S2). Across both conservative and optimistic scenarios, the ICER/LYS ratio comparing LEN with TDF/FTC consistently remained below 1, confirming LEN's cost-effectiveness advantage. Likewise, the LYS ratio remained above 1, indicating greater effectiveness of LEN. The parameters with the greatest influence on cost-effectiveness were cost and risk-stratified uptake, while LEN uptake rates and TDF/FTC duration had the biggest impact on LYS (Figure S2). In addition, CAB remained less cost-effective than TDF/FTC scale-up, unless CAB prices were reduced or CAB use was more strongly targeted toward higher risk populations (Figure S3). When risk-targeted LEN delivery within the AGYW population was assumed to be less efficient within the Phase 1 Global Fund backed roll-out, the relative priority of AGYW versus PBFW became less clear; however, FSW and MSM consistently remained high-priority populations for achieving maximum impact (Figure S4). In the Phase 2 national roll-out, varying high-risk uptake amongst AGYW and increasing service delivery costs for FSW and MSM, altered ICER magnitudes, but did not materially change the ranking of strategies, with prioritization of FSW and MSM remaining most cost-effective strategies (Table S7)."*<br><br>We further include a point in our Discussion regarding how Thembisa handles risk-

Comment	Response
	differentiated uptake compared to other models (lines 447-451):<br>*"Stansfield et al also show that Thembisa is more conservative in its estimates of effective coverage than those in comparable models such as Synthesis or EMOD, suggesting that Thembisa's assumptions about risk-differentiated uptake are more conservative than those of comparable models, meaning our estimates of infections averted may already represent a cautious lower bound [34]."*
2) The constrained allocation exercise needs clearer decision-analytic specification<br><br>The early-supply allocation analysis is one of the paper's most valuable contributions, but it currently reads more like a set of scenario comparisons than a formally defined constrained optimisation problem. For a policy audience, it is essential to be explicit about:<br><br>the objective function being optimised (e.g., infections averted vs life-years saved),<br>the constraint (e.g., person-years, initiations, doses, budget),<br>the time horizon over which the objective is evaluated, and<br>the feasibility constraints applied<br><br>Without this, "optimised allocation" can be interpreted as universally optimal, when in fact it is conditional on the chosen objective and constraint.	Thank you for pointing this out. We have edited the wording in the methods to clearly outline the objective, time horizons and constraints. The paragraph now reads (lines 171-182):<br>*"We modelled the impact of Phase 1 with the objective to maximize new HIV infections averted over a 5-year time horizon (2026-2030), reflecting primary and short-term secondary infections averted. The binding constraint was having 500,000-person years of LEN for 2026-2027, and no LEN from 2028 onwards, so as to isolate the impact of Phase 1. For simplicity, we assumed all populations had an average duration on LEN for one year. We modelled 490 combinations of discrete increments of LEN initiations across subpopulations, subject to pre-defined feasibility constraints: maximum coverages of 60% for FSW (45,000 initiations/year) and 30% for MSM (93,500 initiations/year), reflecting realistic upper bounds on reachable key population size (Table S2). We constrained the annual initiations to 250,000, implying a maximum number of initiations each of 250,000 for AGYW and PBFW, corresponding to population coverages of 4% and 20%, respectively. Transgender individuals were not included as Thembisa does not currently model this population dynamically."*
3) Continuation/persistence assumptions remain a key driver and should be treated as a core uncertainty, not a scenario detail. LEN's comparative advantage over oral PrEP is strongly mediated by persistence on product and re-initiation patterns, particularly in populations with time-varying risk (e.g., periods of partnership change, pregnancy/postpartum).	We agree that effective protection is a function of initiation and continuation. To address the potential of variability in uptake and/or persistence and its impact, we have included a one-way sensitivity analysis that varies uptake rates of LEN relative to oral PrEP, the uptake of LEN or oral PrEP by high risk targeting, as well as the duration of oral PrEP being equal to the

Comment	Response
While scenario-based continuation assumptions are reasonable, the paper would be strengthened by making explicit that effective protection time (initiation × continuation × return after discontinuation) is the primary determinant of impact, and including at least one mixed performance scenario that reflects a plausible operational reality (e.g., high initiation but lower persistence, or strong persistence but weaker targeting).	conservative LEN scenario. Changes to the manuscript relating to this are made in comment (1) above.
4) The paper presents ambitious scale-up trajectories. The missing step is to interpret what those imply for service delivery in South Africa in terms of:<br><br>clinic throughput<br>staff time requirements<br>stock management and continuity of injectable supply<br>the degree to which injectable PrEP competes for capacity with other high-priority services<br><br>This matters because the feasibility bottleneck for injectables is often not demand, but delivery capacity. If delivery constraints bind, realised coverage and persistence will fall below assumed levels, and the incremental advantage of LEN may narrow. Could the authors include a short operational feasibility discussion that links the projected scale-up volumes to plausible delivery platforms (ANC, PHC, differentiated services, KP services) and explicitly states which constraints are not represented structurally in the model? This does not require new modelling, but materially improves interpretability.	Thank you. We agree that this addition would strengthen policy relevance. While the model does not structurally capture facility capacity, supply chain constraints, staff time requirements or how injectable PrEP competes for capacity with other services, we have included a paragraph in the discussion to outline these as issues that need to be considered for any injectable PrEP roll-out when going to scale.<br><br>We have included the following into the Discussion (lines 423-432):<br>*"First, the feasibility of delivering our modelled scenarios is currently unknown. We included costs for demand creation, but these may not be sufficient to reach the assumed large-scale uptake. Though clients could be reached through existing platforms within primary and antenatal healthcare, South Africa may need to re-establish key population services for reaching MSM and FSW, and this may require additional resources. The volumes modelled are large, and could put additional pressure on the delivery and health infrastructure overall. Several implementation constraints are not captured structurally in our model, including clinic throughput, availability of trained personnel and consumable supply continuity. Should any of these constraints bind in practice, realised coverage would fall below modelled levels, reducing infections averted and narrowing LEN's cost-effectiveness advantage over oral PrEP."*
5) Comparisons between oral PrEP, CAB, and LEN are highly sensitive to assumptions about persistence and discontinuation, switching between products, visit schedules and	Thank you. We agree with the reviewer that persistence, switching between products and behavioural response (such as condom disinhibition)

Comment	Response
behavioural response etc. Even when each modality is modelled reasonably, differences in these assumptions can unintentionally embed a programme advantage for one modality. Please add a brief statement explaining how you ensure comparability across modalities (i.e., that differences in outcomes reflect product and delivery characteristics rather than inconsistent modelling choices). If you already have a harmonised framework, make it explicit in the main text.	is a key driver of comparative results.<br><br>All PrEP options are evaluated within the same harmonised Thembisa framework, using identical population structure, risk stratification, transmission dynamics, ensuring that structural modelling choices do not systematically favour any single product.<br><br>Regarding persistence and duration, we note that the ICERs of each modality relative to baseline are what drive the cost-effectiveness ranking, and the sensitivity analysis demonstrates that the ranking of LEN over oral PrEP is robust across a wide range of duration assumptions. Visit schedules differ across PrEP options but reflect each product's actual dosing regimen (LEN: 2/year; CAB: 7/year; TDF/FTC: 5/year) and are therefore a product characteristic and are accounted for in the costing.<br><br>Thembisa does not model option switching explicitly; however, in the context of an assumed short duration of PrEP, it would not impact ICERs greatly to switch between PrEP modalities (without an interruption) or discontinuation followed by re-initiation (of a potentially different PrEP product). In terms of behavioural response, the model applies the same assumption of a 10% reduction in condom use for all PrEP options. This does not confer an advantage to any single product. One could argue that higher efficacy products might induce greater risk compensation, but given the very high efficacy of LEN (99%), it would still avert more infections than oral PrEP, providing substantial headroom for differential risk compensation before the comparative cost-effectiveness advantage would be reversed.<br><br>To address this comment, the following was added to the Methods (lines 168-173):<br>*"To ensure comparability, all PrEP options were modelled within the same harmonised Thembisa framework using identical population structure, risk stratification, transmission dynamics, with behavioural response to PrEP*

Comment	Response
	applied uniformly across all modalities. Differences in effectiveness, duration of use, visit schedules, and tail protection reflect evidence-based product characteristics, derived from South African programme data for oral TDF/FTC and from clinical trial data for injectable PrEP."
6) The model stratifies populations in a way that is appropriate for national planning, but PrEP cost-effectiveness and prioritisation can be highly sensitive to geographic heterogeneity (incidence hotspots, urban service access, mobility-linked risk) and to within-group risk gradients. A short discussion of how geographic targeting (or hotspot prioritisation) could alter the optimal early allocation of LEN, and whether the main conclusions are expected to be robust or could change under more granular targeting would be useful.	We thank the reviewer for this important point. We agree that hotspot prioritisation would likely increase cost-effectiveness further but is outside the scope of our national-level model. We have expanded the Discussion to address how geographical targeting/prioritisation would likely further improve cost-effectiveness beyond our national estimates, and to note that within-group risk gradients not captured in our population-level stratification could shift prioritisation conclusions at a finer geographical level (lines 407-414):<br>*"Geographic prioritisation could also refine the Phase 1 LEN roll-out more broadly: in high-incidence settings, a greater share of the available doses could be directed towards populations facing the most acute risk, while in lower incidence regions the marginal benefit of additional initiations may be limited across all subpopulations. In addition, the size of key populations may vary markedly by geography, with FSW and MSM being more concentrated in urban settings, potentially limiting the number of initiations available in lower-density or rural settings and concentrating the cost-effectiveness gains of FSW- and MSM-targeted strategies in urban delivery platforms."*
This is a strong submission with substantial policy relevance. The most important improvements to strengthen scientific robustness and decision credibility are:<br><br>1. a structural sensitivity analysis for imperfect targeting within groups,<br>2. a clearer formal definition of the constrained allocation objective and constraint, and<br>3. explicit treatment of persistence and delivery feasibility as the primary determinants of real-world impact.<br><br>Addressing these points would substantially	We thank the reviewer again for all their feedback and hope the above responses are sufficient

<table>
<tr><th>Comment</th><th>Response</th></tr>
<tr><td>increase confidence that the prioritisation conclusions will hold under realistic implementation conditions in South Africa.</td><td></td></tr>
<tr><td colspan="2">Reviewer #2</td></tr>
<tr><td colspan="2">General comment: Jamieson and colleagues examined the epidemiological impact and cost effectiveness of integrating lenacapavir into South Africa's HIV prevention response. This is a timely and important question: lenacapavir has demonstrated higher efficacy than oral PrEP, may help overcome the adherence challenges commonly seen with daily oral regimens, and is now available at a price of 40 USD. The authors used the well established Thembisa model—South Africa's primary HIV transmission model—to simulate the introduction of oral PrEP, cabotegravir, and twice yearly injectable lenacapavir (LEN) from 2026 to 2045. Their analysis focused on key populations at elevated HIV risk, including adolescent girls and young women, sex workers, pregnant and breastfeeding women, men who have sex with men, and heterosexual men.
The study found that LEN produced the greatest epidemiological impact. If 1.7-2.9 million people used LEN annually, it could avert roughly one fifth to one third of new HIV infections and preserve several percent of the life years otherwise expected to be lost to HIV. LEN scale up could also reduce national HIV incidence to below 0.1% more than a decade earlier than projected under current trends.
When LEN supply is limited, prioritising pregnant and breastfeeding women, sex workers, and men who have sex with men yields the largest reduction in infections. For broader national roll out, prioritising sex workers and MSM remains the most cost effective strategy.
Overall, providing LEN to individuals at highest HIV risk in South Africa appears substantially more cost effective than expanding existing PrEP options and could accelerate progress toward reducing HIV incidence.
The manuscript is well written and provides valuable insights. I do, however, have several comments.</td></tr>
<tr><td>1. Assumptions favouring lenacapavir uptake
The model assumes that LEN uptake matches oral TDF/FTC in the conservative scenario and is twice as high in the optimistic scenario. These assumptions imply equal or greater preference for LEN over oral PrEP, which is not clearly supported by existing preference studies in other settings. Can the authors provide evidence that individuals at risk of HIV prefer LEN at these levels? Should a third scenario be included in which LEN uptake is lower than oral PrEP?</td><td>Thank you for highlighting this important. There is some is evidence regarding preferences for LEN vs oral PrEP uptake. While preference studies in SSA have shown a higher preference for long-acting products, how these preferences will translate into real-world uptake, and to what extent, remains uncertain. For this reason, we have added a one-way sensitivity analysis where we vary several parameters, including uptake of LEN relative to oral PrEP with a lower bound where LEN uptake is half of that of oral PrEP, and assess the relative impact on cost-effectiveness and life years saved, compared to an oral PrEP scale-up scenario.

We have included the following in our Methods to justify these assumptions better and citing a scoping review of preference studies done on long-acting products in Eastern and Southern Africa by Pfau et al (2025) as well as a South African study conducted within the CAB trial</td></tr>
</table>

Comment	Response
	(Delany-Moretlwe 2023, presented at AIDS 2023) at lines 184-185:<br>*"These scenarios imply a similar or higher preference for long-acting products compared to TDF/FTC, a decision that can be justified by preference studies conducted in the local or regional context [20, 21]"*<br><br>Related to the sensitivity analyses, we have added the following in Methods, lines 258-263:<br>*"We conducted a one-way sensitivity analysis within our main analysis to evaluate the impact of key PrEP parameters, including uptake, differential risk targeting across PrEP options, TDF/FTC duration, LEN/CAB tail protection, and costs. We also conducted sensitivity analysis to examine whether improved or poorer risk targeting, or higher service delivery costs for FSW or MSM, affected impact of different subpopulation distributions in Phase 1, or the relative cost-effectiveness of LEN scale-up in Phase 2 of our third analysis."*<br><br>We added a tornado diagram in the supplementary appendix (Figures S2 and S3), and updated the Results section, lines 343-356:<br>*"One-way sensitivity analyses showed that the cost-effectiveness and impact of LEN, relative to TDF/FTC scale-up, were robust (Figure S2). Across both conservative and optimistic scenarios, the ICER/LYS ratio comparing LEN with TDF/FTC consistently remained below 1, confirming LEN's cost-effectiveness advantage. Likewise, the LYS ratio remained above 1, indicating greater effectiveness of LEN. The parameters with the greatest influence on cost-effectiveness were cost and risk-stratified uptake, while LEN uptake rates and TDF/FTC duration had the biggest impact on LYS (Figure S2). In addition, CAB remained less cost-effective than TDF/FTC scale-up, unless CAB prices were reduced or CAB use was more strongly targeted toward higher risk populations (Figure S3). When risk-targeted LEN delivery within the AGYW population was assumed to be less efficient within the Phase 1 Global Fund backed roll-out, the relative priority of AGYW versus PBFW became less clear;*

Comment	Response
	however, FSW and MSM consistently remained high-priority populations for achieving maximum impact (Figure S4). In the Phase 2 national roll-out, varying high-risk uptake amongst AGYW and increasing service delivery costs for FSW and MSM, altered ICER magnitudes, but did not materially change the ranking of strategies, with prioritization of FSW and MSM remaining most cost-effective strategies (Table S7).”
2. Longer duration of LEN use<br>LEN is modelled with the longest duration of use (6-12 months), compared with 4-8 months for cabotegravir and 3-6 months for oral PrEP. This inherently gives LEN a longer period of protection. I recommend adding an analysis in which all PrEP modalities are assigned the same duration of use to isolate the effect of the drug itself from the effect of duration.	This is an important point as well that we have attempted to address with the one-way sensitivity analysis in which the upper bound of the duration on oral PrEP is aligned with the 6-12 month duration represented by the LEN scenario. See above additions described in. Changes to the manuscript relating to this are made in comment (1) above.<br><br>In this particular instance, LEN remains more cost-effective and more effective compared to oral PrEP even if the duration of TDF/FTC is similar.
3. Effectiveness during the pharmacokinetic tail<br>The authors appropriately include the pharmacokinetic tail of long acting PrEP. However, it is unclear whether the model incorporates the reduced effectiveness of cabotegravir during the tail phase, which has been documented in the literature (e.g., Landovitz et al., Lancet HIV 2020; Moore et al., IAS 2025). The tail phase for LEN remains unknown.	We appreciate this point and clarify that our model implicitly accounts for reduced effectiveness during the tail phase through its structural assumptions. In Thembisa, we represent individual heterogeneity in tail protection duration by assuming it follows an exponential distribution. The implication of this is that average protection declines continuously with time since the last missed dose, rather than dropping abruptly to zero at the mean tail duration. For example, if the average tail duration is 6 months, average protection at 3 months after the last missed dose is exp(-3/6) = 61% of full protection. The model therefore does not assume full effectiveness throughout the tail; effectiveness reduces gradually and continuously, which is consistent with the pharmacokinetic evidence cited by the reviewer (Landovitz et al., 2020 [19]). We have added a clarifying sentence to the Methods to make this explicit (lines 164-166):<br>*“Additionally, the model captures variability in tail duration using an exponential distribution, so effectiveness declines gradually after a missed dose rather than dropping abruptly,*

Comment	Response
	reflecting pharmacokinetic evidence of reduced tail-phase protection [19].”
4. Length of the tail phase<br>Related to point 3, the model assumes a 3 month tail for cabotegravir, which appears to underestimate the duration reported in published studies. Conversely, the assumed 6 month tail for LEN may be an overestimate. Although LEN's tail is not yet characterised, available pharmacokinetic data (Jogiraju et al., Lancet 2025) suggest that six months may exceed the tail duration for some individuals.	Thank you for pointing this out. Indeed, tail duration remains uncertain and we attempted to account for it in probabilistic sensitivity analysis by varying the length of the tail phase. To help characterize the length of tail protection more clearly and understand its impact on cost-effectiveness and effectiveness on HIV prevention, we conducted a one-way sensitivity analysis where we varied the tail duration with a 50% reduction and 50% increase, with LEN ranging between 3 and 9 months. Overall the results show that LEN remains more cost-effective than oral PrEP scale up. Even with a tail protection of 3 months, the incremental cost-effectiveness per life year saved of LEN scale-up over baseline was 50%-67% less than the same cost-effectiveness metric of oral PrEP scale-up.<br><br>Changes to the manuscript relating to the one-way sensitivity analysis this are made in comment (1) above.
**Reviewer #3**	
General comment: This is a well conducted study on the epidemiological impact on HIV infections and life years lost, cost-effectiveness, and optimal populations for Lenacapavir roll-out in South Africa. The study design, statistical methods and analyses, and presentation (tables and figures) and interpretation of the results are mostly adequate and of a good standard. However, still a few issues needing attention.	
1. Assumption and scenarios. Table 1 shows Key assumptions on coverage, duration and effectiveness for main scenarios. However, there are too many assumptions and combinations which are difficult to follow. Usually, we have projected, worst and best scenarios, but here scenarios are all over the place so that the table becomes less informative and key messages are a bit lost. Is there any innovative way to summarise the key messages in the table?	Thank you for pointing this you. We acknowledge that Table 1 is necessarily complex. We simplified this table and moved the more complex version to the Supplementary Appendix to give additional detail regarding the coverage levels by subpopulation. In addition, we added a footnote to Table 1 to specify this as well: *“A more detailed version of this table depicting modelled coverage levels by subpopulation is presented in the Supplementary Appendix, Table S1.”*
2. Table 2 shows Impact and cost-effectiveness of oral TDF/FTC, injectable LEN or CAB scale-up compared with baseline over 2026-45. What are the models for projections here? deterministic or probabilistic?	The estimates in Table 2 are specifically the deterministic point estimates, whereas probabilistic estimates are presented in Table S8 with 95% uncertainty bounds. We have made this distinction clearer in the manuscript.

<table>
<tr><th>Comment</th><th>Response</th></tr>
<tr><td></td><td>In methods (lines 196-197), we added: “Results presented are deterministic point estimates and key assumptions are detailed in Table 1.”

In addition, we have added a footnote to Table 2 where the deterministic results are presented to say: “Results represent deterministic point estimates from the central scenario. Probabilistic sensitivity analysis results with 95% uncertainty bounds are presented in the Supplementary Appendix, Table S8.”</td></tr>
<tr><td>3. Table 3 comparing 20-year impact (2026-2045) and cost-effectiveness of LEN and CAB scale-up scenarios to existing HIV interventions, with incremental cost effectiveness ratios compared to baseline. Again, what are the models used for ICER calculation? Have Markov models been used for ICER calculation and projection?</td><td>The Thembisa model has also been used for this projection, with resulting impact and costs analysed to estimate ICERs and presented. We have amended the methods to clarify this (lines 199-201): “For the second analysis, we used Thembisa to model the impact and cost per life-year saved of PrEP scale-up to expansion of other existing HIV interventions, similar to previous analysis for the annual South African HIV Investment Case.”</td></tr>
<tr><td colspan="2">Reviewer #4</td></tr>
<tr><td colspan="2">General comment: This is an interesting and relevant modeling study assessing the impact of scaling up LEN in the context of other HIV prevention interventions including CAB and oral PrEP. While previous studies have evaluated LEN scale up, to my knowledge, this is the first to assess it in the context of other strategies.</td></tr>
<tr><td>1) The authors focus the abstract and the discussion on the finding than LEN is more cost-effective than other HIV prevention interventions. I think the abstract should also highlight the important finding that LEN provision is less cost-effective than increasing ART, HIV testing, and MMC coverage. Indeed increasing ART coverage can save substantially more lives. And in the context of the likely funding disruptions, testing and treatment interventions will become even more cost-effective. This can also be emphasized more in the discussion, eg 8 times more life years are saved with ART expansion compared to LEN.</td><td>Thank you. We agree this is an important finding currently underweighted, particularly in the abstract. We have included reference to this finding in both Abstract: “While promising for HIV prevention, scaling up condoms, ART, HIV testing and medical male circumcision may be more cost-effective than LEN; notably ART at 95% coverage, saved 4.8-8-fold more life years compared to LEN, costing $577/life year saved.”

And Discussion (lines 376-379): “Compared to other interventions, expanding ART to 95% coverage of all diagnosed PLHIV was the only intervention exhibiting greater cost-effectiveness and higher impact in terms of both life years (4.8-8-fold more life years saved) and HIV infections (1.7-2.8-fold more infections averted) compared to LEN scale-up.”</td></tr>
<tr><td>2) The authors mention than successful LEN scale up will depend on efficient delivery and</td><td>While we varied injectable PrEP delivery costs in the probabilistic sensitivity analysis, we</td></tr>
</table>

Comment	Response
demand generation. The PrEP delivery costs are robustly estimated using ingredients based costing. However, the authors estimate that the cost of LEN delivery is similar to that of oral PrEP (eg same staff time and salary costs). It is possible that LEN delivery costs may be higher than oral PrEP due to higher cadre personnel administering injections, consumables, and increased counseling/post injection observation time. This may increase LEN costs relative to oral PrEP (although likely outweighed by the less frequent visits). The authors may consider a sensitivity analysis to asses this.	acknowledge that this approach may not clearly isolate the impact of higher delivery costs on the cost-effectiveness of LEN relative to oral PrEP. We have therefore added a one-way sensitivity analysis in which LEN delivery costs are varied from 50% of the base-case value up to two-fold higher, and we evaluate the effect on the incremental cost-effectiveness ratio (ICER) per life-year saved (LYS) for LEN versus baseline, compared with oral PrEP scale-up versus baseline.<br><br>In these sensitivity analyses, delivery costs increased from \$23–\$28 to \$46–\$56 per person in the conservative LEN scenario, and from \$29–\$40 to \$58–\$80 per person initiated in the optimistic LEN scenario (which assumes longer durations of use). As expected, higher LEN delivery costs reduced cost-effectiveness; however, LEN remained more cost-effective than oral PrEP scale-up, with the ICER/LYS ratio remaining at least 33% lower for LEN.<br><br>We added the following in Methods, lines 258-263:<br>*"We conducted a one-way sensitivity analysis within our main analysis to evaluate the impact of key PrEP parameters, including uptake, differential risk targeting across PrEP options, TDF/FTC duration, LEN/CAB tail protection, and costs. We also conducted sensitivity analysis to examine whether improved or poorer risk targeting, or higher service delivery costs for FSW or MSM, affected impact of different subpopulation distributions in Phase 1, or the relative cost-effectiveness of LEN scale-up in Phase 2 of our third analysis."*<br><br>We added a tornado diagram in the supplementary appendix (Figures S2 and S3), and updated the Results section, lines 343-356:<br>*"One-way sensitivity analyses showed that the cost-effectiveness and impact of LEN, relative to TDF/FTC scale-up, were robust (Figure S2). Across both conservative and optimistic scenarios, the ICER/LYS ratio comparing LEN with TDF/FTC consistently remained below 1, confirming LEN's cost-effectiveness advantage.*

Comment	Response
	Likewise, the LYS ratio remained above 1, indicating greater effectiveness of LEN. The parameters with the greatest influence on cost-effectiveness were cost and risk-stratified uptake, while LEN uptake rates and TDF/FTC duration had the biggest impact on LYS (Figure S2). In addition, CAB remained less cost-effective than TDF/FTC scale-up, unless CAB prices were reduced or CAB use was more strongly targeted toward higher risk populations (Figure S3). When risk-targeted LEN delivery within the AGYW population was assumed to be less efficient within the Phase 1 Global Fund backed roll-out, the relative priority of AGYW versus PBFW became less clear; however, FSW and MSM consistently remained high-priority populations for achieving maximum impact (Figure S4). In the Phase 2 national roll-out, varying high-risk uptake amongst AGYW and increasing service delivery costs for FSW and MSM, altered ICER magnitudes, but did not materially change the ranking of strategies, with prioritization of FSW and MSM remaining most cost-effective strategies (Table S7).”<br><br>In addition, we have included this point in the Discussion (lines 433-438):<br>*“The biggest cost difference between PrEP options lies in the drugs, but further research is needed to determine the true implementation costs and explore innovative delivery methods for injectable PrEP to enhance its cost-effectiveness, especially amongst key populations. However, sensitivity analyses varying delivery costs up to three-fold did not materially alter the cost-effectiveness advantage of targeting key populations.”*
3) Similarly, demand generation costs have been found to be substantial in oral PrEP programs. This is particularly important to achieve high uptake and continuation of LEN at the proposed levels and in the hard to reach populations. It would be helpful for the authors to conduct sensitivity analyses around the demand generation costs as it is not as robustly estimated as the provision costs.	We agree this is an important point, particularly given recent funding disruptions, including PEPFAR withdrawal, which have affected programmes serving hard-to-reach populations and may have implications for demand generation costs. Our LEN provision/delivery cost estimates include demand generation costs, and these provision costs overall are varied in the one-way sensitivity analysis described in our previous response. We believe

Comment	Response
	this addresses the reviewer's concern by examining the impact of higher delivery and demand generation costs on the cost-effectiveness of LEN. Changes to the manuscript relating to the one-way sensitivity analysis this are made in comment (2) above.
4) In light of the newly estimated generic LEN cost of $25 ppy (plus loading dose) it might be helpful to run the CEA and BIA with the lower cost LEN product to highlight differences in efficiency and affordability.	Thank you for pointing this out. We have included this in our one-way sensitivity analysis to measure the impact of a lower drug cost on cost-effectiveness of LEN. Changes to the manuscript relating to the one-way sensitivity analysis this are made in comment (2) above.<br><br>We have also included a scenario for the BIA where we reduce the cost of LEN to $25 PPPY to estimate the annual budget required, with BIA results presented in the Supplementary Appendix in Table S5.
5) (Optional) Due to the high potential of funding cuts/PEPFAR disruption, the authors should consider running a scenario comparing the HIV prevention interventions in the context of lower ART coverage and HIV testing. In this case, interventions to increase HIV testing and ART coverage may be more efficient than LEN or other prevention strategies as they immediately save lives.	We appreciate this thoughtful suggestion. To date, there is limited evidence that ART coverage has been substantially affected at the national population level, although HIV testing has been impacted in some districts where services were primarily delivered through PEPFAR-funded partners.<br><br>While this analysis is beyond the scope of the current manuscript, we are currently undertaking related work aligned with the reviewer's suggestion, in which we assume reductions in ART coverage, HIV testing, MMC, and PrEP for MSM and FSW, and evaluate the impact of protecting core HIV prevention interventions and ART, including the potential role of LEN. We agree this is an important question, particularly in the context of funding uncertainty, and we see it as a valuable area for separate analysis rather than incorporating it into the present study.
6) In the limitation section, the authors should mention that the use of a compartmental model makes it harder to estimate the impact of partnership-level prevention strategies like PrEP. This could result in underestimation of LEN cost-effectiveness which may be why ICERs are higher than those of other network models (in addition to the higher provision costs assumed).	We have added the following in the Discussion section: "Fourth, we have used a frequency-dependent compartmental model to represent heterogeneity in HIV risk; such models are likely to understate the fraction of transmission that can be prevented by targeting interventions to high-risk groups [33]. This might partially explain why our modelled impacts of injectable

Comment	Response
	PrEP are lower than those estimated using network models [34].”

Sincerely,

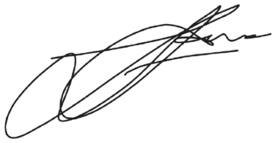

Lise Jamieson, PhD
Health Economics and Epidemiology Research Office (HE^{2}RO)
University of the Witwatersrand, Johannesburg, South Africa
Email: ljamieson@heroza.org
