# Supplementary material for: Optimising scale-up of injectable lenacapavir for HIV pre-exposure prophylaxis in South Africa: A modelling study and economic evaluation
Source: PLoS Med. 2026 Jul 21;23(7):e1004882. doi: 10.1371/journal.pmed.1004882 (PMC13421763; doi:10.1371/journal.pmed.1004882)
Supplement: Attachment — Submitted filename: Responses to reviewers and editorial requests 23.06.2026.docx [file pmed.1004882.s007.docx]

**Requests from Editors:**

| **Request** | **Response** |
| --- | --- |
| Please define all abbreviations at first use, including USD.  For USD in the main text, please define at first occurrence (i.e. not in the Methods). | Thank you for picking this up. We have defined USD upon first use in the main text (lines 44-45) and double checked the entire manuscript to ensure that all abbreviations are correctly defined. |
| Line 54: “under FSW scale-up” as meant? Or should this be “under scale-up to FSW”? | Thank you for correcting this, yes grammatically it should be “under scale-up to FSW”, we have corrected it as such. |
| Author summary:  First bullet point: please mention HIV. Please also use ‘the Global Fund’, for consistency. | We have added HIV to the author summary and made sure to refer to “the Global Fund” consistently here and throughout the manuscript. |
| Line 73: add ‘or’ to read ‘or another injectable option’ | Thank you. We have made this change. |
| GENERAL EDITORIAL REQUESTS  * Please revise your title so that it complies with PLOS Medicine's style. Your title must be nondeclarative and not a question. It should begin with main concept if possible. "Effect of" should be used only if causality can be inferred, i.e., for an RCT. Please place the study design ("A randomized controlled trial," "A retrospective study," "A modelling study," etc.) in the subtitle (ie, after a colon). For example: Scale-up of HIV pre-exposure prophylaxis in South Africa: An economic evaluation | Thank you. We have made another minor addition to this to refer to the work as both a modelling study and economic evaluation, and to write out PrEP in full since it is a specialist abbreviation to the field of HIV: *“Taking injectable pre-exposure prophylaxis to scale: A modelling study and economic evaluation to optimise lenacapavir for South Africa’s HIV response”.* |
| * Please confirm that your abstract complies with our requirements, including format (three sections: Background, Methods and Findings, and Conclusions) and providing all the information relevant to this study type <https://journals.plos.org/plosmedicine/s/submission-guidelines#loc-abstract> | We confirm that the abstract complies with the requirements. |
| * Please ensure that the Introduction ends with a clear description of the study question or hypothesis. | Our last paragraph in the Introduction (lines 137-144) describes our study clearly *“To inform the prioritization of the planned roll-out of LEN in the South Africa, we conducted three economic evaluations: (1) we estimated the cost and cost-effectiveness of a large-scale roll-out of LEN at the generic price of $40 PPPY, compared to CAB and oral TDF/FTC scale-up, and produced budget estimates for the South African government; (2) we compared both LEN and CAB roll-out to scaling up other existing HIV interventions, including testing, prevention and treatment; and (3) we modelled several roll-out strategies to different subpopulations to optimise the epidemiologic impact of the initial 2-year LEN allocation (~500,000 person-years annually in 2026 and 2027) (Phase 1) and the planned large-scale roll-out from 2028 onwards (Phase 2).”* |
| * Please ensure that all abbreviations are defined at first use throughout the text. | We have revised the manuscript and ensured that all abbreviations are defined upon first use. |
| * Please confirm that all numbers presented in the abstract are present and identical to numbers presented in the main manuscript text. | We have revised the abstract and ensured that they are identical to the main manuscript. |
| * Please convert any stacked bar charts to another data representation for example a table, or other type of graph. | We have converted Figure 3 from a discrete stacked vertical bar chart into a continuous stacked area chart to help smooth out some of the jagged edges that may be problematic with such a dense figure. Due to the complex nature of the modelling outputs created in this subanalyses, where 490 distinct allocation combinations are arranged continuously along the x-axis by overall epidemiological impact, representing this in a figure is somewhat restrictive. To aid transparency, we have provided the complete, unedited raw data matrix containing the exact percentages and infection outcomes for all 490 model runs as supplementary information. |
| * Please disclose any use of AI tools and technologies to the study or article content at the end of the Methods. Please specify the name(s) and version(s) of any tool(s) used and a description of their use, including how the AI-assisted or generated content was evaluated by authors. Please also include a statement specifying who retains responsibility for the final manuscript. Please see our guidelines for more information: <https://journals.plos.org/plosmedicine/s/ethical-publishing-practice#loc-artificial-intelligence-tools-and-technologies>. | We have included a statement in the Methods to disclose the use of AI, lines 292-297:  *“Use of artificial intelligence tools and technologies*  *During the preparation of this manuscript, the authors utilized Microsoft Copilot (GPT-4) exclusively to refine grammar and improve phrasing fluidity. No artificial intelligence tools were used to generate original scientific concepts, or perform modeling simulations or analyses. The authors critically evaluated and verified all language refinements to ensure scientific accuracy and contextual integrity. The authors retain full responsibility for the final content and integrity of the completed manuscript.”* |
| FUNDING STATEMENT  * The funding statement should include: specific grant numbers, initials of authors who received each award, URLs to sponsors’ websites. Also, please state whether any sponsors or funders (other than the named authors) played any role in study design, data collection and analysis, the decision to publish, or preparation of the manuscript. If they had no role in the research, include this sentence: “The funders had no role in study design, data collection and analysis, decision to publish, or preparation of the manuscript.” Please include the funder URL(s). | The funding statement is accurate, includes grant numbers and author initials where appropriate, as well as states the role of the funder. We have included the funder URL in the statement. |
| DATA AVAILABILITY  * PLOS defines the “minimal data set” to consist of the data set used to reach the conclusions drawn in the manuscript with related metadata and methods, and any additional data required to replicate the reported study findings in their entirety. Authors do not need to submit their entire data set, or the raw data collected during an investigation. Please confirm that the manuscript includes the following data (note that all may not apply):  The values behind the means, standard deviations and other measures reported; The values used to build graphs; The points extracted from images for analysis.  * Please include the statement on code availability in the data availability statement. Please see guidance here: https://journals.plos.org/plosmedicine/s/code-sharing-guidance#loc-data-availability-statement-examples  We now require a digital identifier for code. Please add the DOI to the data availability statement. | We have updated the data availability statement to include the DOI link to the code repository:  *“The C++ version of Thembisa used for this analysis is publicly available at https://doi.org/10.5281/zenodo.20744379”* |
| FIGURES  * Please provide titles and legends for all figures and tables (including those in Supporting Information files). Please define all acronyms used in each figure or table in its corresponding legend. | Thank you. We have revised the documents and made sure that all legends are listed, and all acronyms are defined where needed. |
| ACKNOWLEDGMENTS  * Please include an Acknowledgments section in your manuscript. | We have included an Acknowledgement section in the manuscript. |
| ECONOMIC ANALYSES INCLUDING COST-EFFECTIVENESS ANALYSES  * Please report your economic analysis according to the appropriate study design provided at <http://www.equator-network.org/?post_type=eq_guidelines&eq_guidelines_study_design=economic-evaluations&eq_guidelines_clinical_specialty=0&eq_guidelines_report_section=0&s>= and provide the relevant completed checklist. In the checklist please include sufficient text excerpted from the manuscript to explain how you accomplished all applicable items. | We have expanded the submitted CHEERS checklist to include text excerpted from the manuscript. |
| MODELING STUDIES  For more details on the requests below please see Geoffrey P Garnett, Simon Cousens, Timothy B Hallett, Richard Steketee, Neff Walker. Mathematical models in the evaluation of health programmes. (2011) Lancet DOI:10.1016/S0140-6736(10)61505-X.  * Please provide a diagram that shows the model structure, including how the disease natural history is represented, the process and determinants of disease acquisition, and how the putative intervention could affect the system. | Because Thembisa v4.8 is a highly comprehensive national transmission framework calibrated to the entire South African population, a complete structural diagram of its internal dynamics is excessively dense for inclusion within the main manuscript. However, the complete representation of the HIV transmission model and detailed schematics of individual components is referenced separately (lines 183-185): "*More detail on the epidemiological model structure, and details of model calibration and sources for assumptions, are available online at Thembisa.org [13]."* Additionally, a focused summary of our specific operational assumptions regarding product effectiveness, risk-differentiated subpopulation profiles, and behavioural dynamics is detailed systematically within the Epidemiological model and Scenarios and assumptions subsections. |
| * Please provide a complete list of model parameters, including clear and precise descriptions of the meaning of each parameter, together with the values or ranges for each, with justification or the primary source cited, and important caveats about the use of these values noted. | Table 1 (and its extension Table S1 in the Appendix) is sufficient for the current description of model parameters (along with the text that refers to these tables further) that are applicable to the current modelling study. For a full set of parameters and detailed assumptions, a reference to the model described in more detail elsewhere is made on lines 183-185: *“More detail on the epidemiological model structure, and details of model calibration and sources for assumptions, are available online at Thembisa.org [13].”* |
| * Please provide a clear statement about how the model was fitted to the data including where relevant goodness-of-fit measure, the numerical algorithm used, which parameter varied, constraints imposed on parameter values, and starting conditions. | We have included a brief sentence describing the model fitting and calibration strategy (lines 153-157): *“Thembisa 4.8 is fitted using a Bayesian approach in which prior distributions are specified for the parameters governing sexual behaviour, HIV transmission and disease progression, and then calibrated against several data sources for HIV prevalence (antenatal clinic data, national household surveys, FSW and MSM prevalence studies) as well as data on recorded deaths, antiretroviral metabolites and age distributions for adult ART patients.”*  With additional reference to the model described in more detail elsewhere (lines 182-184): *“More detail on the epidemiological model structure, and details of model calibration and sources for assumptions, are available online at Thembisa.org [13].”* |
| * For uncertainty analyses, please state the sources of uncertainties quantified and not quantified this can include parameter, data, and model structure. | We have modified Table S3 in the Supplementary Appendix to include the sources of the uncertainty parameters. |
| * Please provide sensitivity analyses to identify which parameter values are most important in the model. Uncertainty estimates seek to derive a range of credible results on the basis of an exploration of the range of reasonable parameter values. The choice of method should be presented and justified. | Methods for both one-way sensitivity analyses and probabilistic sensitivity analyses are described in lines 270-284 with: *“We conducted a one-way sensitivity analysis within our main analysis to evaluate the impact of key PrEP parameters, including uptake, differential risk targeting across PrEP options, TDF/FTC duration, LEN/CAB tail protection, and costs. We also conducted sensitivity analysis to examine whether improved or poorer risk targeting, or higher service delivery costs for FSW or MSM, affected impact of different subpopulation distributions in Phase 1, or the relative cost-effectiveness of LEN scale-up in Phase 2 of our third analysis. Further, a probabilistic sensitivity analysis was conducted to evaluate the impact of the uncertainty of 58 key parameters in Thembisa on the main cost-effectiveness analysis [13], including PrEP-specific parameters such as PrEP effectiveness, duration on TDF/FTC, tail protection for injectable PrEP, and reduction in condom use while on PrEP. We varied the cost of LEN service delivery (excluding drug costs) with lower and upper bounds varying by 50% from our average estimate. A Monte Carlo simulation, encompassing 1,000 iterations, sampled data from pre-determined distributions associated with PrEP-related key parameters as described in Table S3 in the Supplementary Appendix. We report median estimates across cost and health outcomes with 95% uncertainty bounds (UB) estimated using 2.5th and 97.5th percentiles.”* With results presented in Figures S2-S5. |
| * Please discuss the scientific rationale for this choice of model structure and identify points where this choice could influence conclusions drawn. Please also describe the strength of the scientific basis underlying the key model assumptions. | We have included in the methods for this specific choice of model (lines 148-153):  *“This modelling framework is particularly appropriate for a mature, large-scale epidemic, as it captures population-level dynamics while representing HIV acquisition, disease progression, and mortality. The compartmental structure allows integration of prevention and treatment interventions across heterogeneous risk groups, providing a transparent and reproducible basis for evaluating epidemiological impact and cost-effectiveness under different implementation scenarios.”*  In the discussion we mentioned how this model may differ from network models (another potential option for modelling (lines 483-487): “*Fifth, we have used a frequency-dependent compartmental model to represent heterogeneity in HIV risk; such models are likely to understate the fraction of transmission that can be prevented by targeting interventions to high-risk groups [33]. This might partially explain why our modelled impacts of injectable PrEP are lower than those estimated using network models [34].”* |

**Requests from Reviewers:**

| **Comment** | **Response** |
| --- | --- |
| **Reviewer #4** |  |
| The authors have addressed the majority of the reviewer comments which have strengthened the manuscript.  A few minor comments remain:  In determining the optimal allocation authors state that they assumed all populations had an average duration on LEN for one year. This is an uncertain assumption can different persistence among different populations can impact targeted for optimal LEN allocation (eg if some subgroups have higher persistence than others). For example, pregnant women are more likely to discontinue oral PrEP after delivery, this pattern may also apply to LEN. Although the authors vary LEN persistence from 6 months to 1 year, this is done uniformly. It would be helpful to note this in the limitation section as source of uncertainty. | Thank you to the reviewer for taking the time again to review our work and providing their valuable input to improve it.  We agree that this is an uncertainty and something which will hopefully be more accurately measured with a larger scale roll-out within implementation. We have added this as a limitation in the discussion (lines 471-477): *“Third, while we did vary LEN use duration from 6 to 24 months across scenarios and populations, we assumed that AGYW, PBFW and heterosexual men would maintain similar duration of use across scenarios, while MSM had longer durations, loosely based on what we know from the oral PrEP programme. In reality, persistence may differ by subgroup and this differential persistence could shift the optimal targeting of LEN. Real-world data from the upcoming roll-out will be critical to better characterising population-specific persistence and refining these assumptions in future modelling.”* |
| The authors acknowledge their highly optimistic coverage assumptions, particularly among MSM and FSWs. However, the paper does not present sensitivity analyses varying coverage among these groups. The authors should consider evaluating this or providing advice to policymakers in the discussion section regarding optimal allocation of extra doses if target population coverage is not reached. For example, should doses that could not be delivered to MSM or FSWs be allocated to pregnant women or AGYW? What should happen with extra doses if coverage among these target populations is not realized or continuation is lower than expected? | We thank the reviewer for raising this important practical consideration. We have partially addressed this through modelling different coverage levels for a limited LEN supply over 2026-27 in our Phase 1 analysis (presented in Figure 3), and this provides a basis for our advice.  Therefore, to add clarity to this point and some practical advice to policy makers, we have included the following in the Discussion (lines 424-432): *“**If doses prioritised for FSW and MSM in the strategic allocation described here are not delivered at the anticipated volumes, impact would be preserved best by allocating additional available stocks to AGYW at higher risk of acquisition and then PBFW in high incidence locations, rather than allowing product to go unused or expire. These lower volumes could be due to, for example, smaller populations sizes than modelled, lower uptake than modelled coverage targets, or lower continuation than assumed, all of which could result from the ongoing defunding of key population programmes. Learning and dynamically adapting programme implementation to reach those at highest risk of acquisition during early programme rollout will be critical to ensure programme attain impacts close to those suggested by our modelling”* |
| Additionally, the authors state in the introduction: "Kaftan et al. estimated a range between $27-$106 PPPY depending on risk prioritisation, while Wu et al. estimated $177-$213 PPPY."  This should say: $32-$213 PPPY for the Wu at all paper as the lowest price per dose were found in Kenya ($16 per 6 monthly dose). The maximum prices varied widely by country and were highest for South Africa. It might be useful to mention in the discussion that this analysis may not be generalizable to other parts of Africa as South Africa is unique in its healthcare system and HIV epidemic. LEN may not be cost effective at current prices elsewhere in ESA. | The estimated prices cited here were specifically for South Africa. We amended this text to be clearer about what we are referring to, but also to note that other countries had lower price estimates (lines 133-136): *“Results varied by epidemic setting according to underlying incidence, risk prioritization, and coverage achieved: Kaftan et al. estimated for South Africa a price range between $23-$106 PPPY and Wu et al. estimated $177-$213 PPPY [11,12], with both studies estimating substantially lower pricing for Zimbabwe and Western Kenya overall.”*  We also added this to the discussion to highlight the lack of generalisability for our results compared to other parts of Africa (lines 450-455): *“Further, while our findings provide useful and practical information for policymakers, our cost-effectiveness results may not be generalisable to other countries in eastern and southern Africa, as South Africa’s health system is fundamentally different in terms of healthcare capacity, domestic funding and resources, as well as underlying HIV incidence and risk-group dynamics. This has been reflected by the variation in price thresholds estimated across the region [11,12]. Country-specific analyses would better capture the programmatic priorities of LEN roll-out in other settings.”* |
