# Supplementary material for: Optimising scale-up of injectable lenacapavir for HIV pre-exposure prophylaxis in South Africa: A modelling study and economic evaluation
Source: PLoS Med. 2026 Jul 21;23(7):e1004882. doi: 10.1371/journal.pmed.1004882 (PMC13421763; doi:10.1371/journal.pmed.1004882)
Supplement: Peer review history

# Editor report: Decision Letter 0

**DOI:** 10.1371/journal.pmed.1004882.r001

**Authors:** Alison Farrell

15 Dec 2025

Dear Dr Jamieson,

Thank you for submitting your manuscript entitled "Taking injectable PrEP to scale: Optimising the value of lenacapavir for South Africa’s HIV response" for consideration by PLOS Medicine.

Your manuscript has now been evaluated by the PLOS Medicine editorial staff, and I am writing to let you know that we would like to send your submission out for external peer review.

However, before we can send your manuscript to reviewers, we need you to complete your submission by providing the metadata that is required for full assessment. To this end, please login to Editorial Manager where you will find the paper in the 'Submissions Needing Revisions' folder on your homepage. Please click 'Revise Submission' from the Action Links and complete all additional questions in the submission questionnaire.

Please also provide some additional brief explanatory information in the Background section of the Abstract for the general reader, and pertaining to why this analysis is important at this time, in this population.

Please remove use of vast (X2) from the abstract and use more objective language.

Please note the study limitations in the last sentence of the Methods & Findings section of the Abstract.

Please explain what Thembisa v4.8 is for the general reader. Please provide greater clarity in the Discussion on potential discrepancies in cost estimates with related studies that you cite.

Please provide some additional clarifying information to the sentence beginning on line 43 of the Abstract.

Please correct phrasing on line 51.

Line 27--please qualify impact (impact on what?).

Please re-submit your manuscript within two working days, i.e. by Dec 17 2025 11:59PM.

Login to Editorial Manager here: https://www.editorialmanager.com/pmedicine

Once your full submission is complete, your paper will undergo a series of checks in preparation for peer review. Once your manuscript has passed all checks it will be sent out for review.

Feel free to email us at plosmedicine@plos.org if you have any queries relating to your submission.

Kind regards,

Alison Farrell, Ph.D.

Senior Editor

PLOS Medicine

# Author comment: Author response to Decision Letter 1

**DOI:** 10.1371/journal.pmed.1004882.r002

19 Dec 2025

Attachment Submitted filename: Response to reviewers 19 Dec 2025.pdf

# Peer review: Decision Letter 1

**DOI:** 10.1371/journal.pmed.1004882.r003

**Authors:** Alison Farrell

5 Mar 2026

Dear Dr Jamieson,

Many thanks for submitting your manuscript "Taking injectable PrEP to scale: Optimising the value of lenacapavir for South Africa’s HIV response" (PMEDICINE-D-25-04398R1) to PLOS Medicine. The paper has been reviewed by subject experts and a statistician; their comments are included below and can also be accessed here: [LINK]

As you will see, the reviewers find the study interesting and timely and have made numerous suggestions to strengthen the results and presentation, including additional sensitivity analyses, consideration of alternate scenarios, and further discussion of study limitations, among other issues. After discussing the paper with the editorial team and an academic editor with relevant expertise, I'm pleased to invite you to revise the paper in response to the reviewers' comments. We plan to send the revised paper to some or all of the original reviewers, and we cannot provide any guarantees at this stage regarding publication.

In addition to these revisions, you may need to complete some formatting changes, which you will receive in a follow up email. A member of our team will be in touch with a set of requests shortly. If you do not receive a separate email within a few days, please assume that checks have been completed, and no additional changes are required.

When you upload your revision, please include a point-by-point response that addresses all of the reviewer and editorial points, indicating the changes made in the manuscript and either an excerpt of the revised text or the location (eg: page and line number) where each change can be found. Please also be sure to check the general editorial comments at the end of this letter and include these in your point-by-point response. When you resubmit your paper, please include a clean version of the paper as the main article file and a version with changes tracked as a marked-up manuscript. It may also be helpful to check the guidelines for revised papers at http://journals.plos.org/plosmedicine/s/revising-your-manuscript for any that apply to your paper.

We ask that you submit your revision by Mar 26 2026 11:59PM. However, if this deadline is not feasible, please contact me by email, and we can discuss a suitable alternative.

Don't hesitate to contact me directly with any questions (afarrell@plos.org).

Best regards,

Alison

Alison Farrell, Ph.D.

Senior Editor

PLOS Medicine

afarrell@plos.org

-----------------------------------------------------------

Comments from the reviewers:

Reviewer #1: This is a timely, policy-relevant modelling analysis addressing an immediate implementation decision- how South Africa should deploy long-acting injectable lenacapavir for PrEP under constrained resources and reduced external funding. The work is clearly motivated, uses an established national HIV model, and provides decision-facing outputs on impact, cost-effectiveness, and prioritisation of limited initial supply.

My comments focus on the issues that most affect the validity and policy interpretation of the results.

1) Targeting performance is the dominant structural uncertainty and needs explicit stress-testing

Your conclusions about LEN's efficiency and the prioritisation of early supply rely heavily on how well uptake is concentrated among those at highest acquisition risk. This is not a minor uncertainty, even modest leakage of uptake towards lower-risk individuals within eligible groups can materially reduce infections averted per initiation and weaken cost-effectiveness.

At present, the analysis implicitly assumes a strong relationship between risk and uptake within groups. In real-world South African PrEP delivery, targeting is imperfect, and scale-up typically expands beyond the highest-incidence individuals first reached.

Perhaps include at least one structural sensitivity analysis where uptake is less well targeted within groups (e.g., a weaker correlation between uptake and underlying incidence, or a defined fraction of initiations occurring among lower-risk individuals). Report whether this changes (i) the ranking of prioritised groups for early LEN, and (ii) the relative advantage of LEN vs CAB and oral PrEP.

This would substantially strengthen the policy reliability of the prioritisation conclusions.

2) The constrained allocation exercise needs clearer decision-analytic specification

The early-supply allocation analysis is one of the paper's most valuable contributions, but it currently reads more like a set of scenario comparisons than a formally defined constrained optimisation problem. For a policy audience, it is essential to be explicit about:

the objective function being optimised (e.g., infections averted vs life-years saved),

the constraint (e.g., person-years, initiations, doses, budget),

the time horizon over which the objective is evaluated, and

the feasibility constraints applied

Without this, "optimised allocation" can be interpreted as universally optimal, when in fact it is conditional on the chosen objective and constraint.

3) Continuation/persistence assumptions remain a key driver and should be treated as a core uncertainty, not a scenario detail. LEN's comparative advantage over oral PrEP is strongly mediated by persistence on product and re-initiation patterns, particularly in populations with time-varying risk (e.g., periods of partnership change, pregnancy/postpartum). While scenario-based continuation assumptions are reasonable, the paper would be strengthened by making explicit that effective protection time (initiation × continuation × return after discontinuation) is the primary determinant of impact, and including at least one mixed performance scenario that reflects a plausible operational reality (e.g., high initiation but lower persistence, or strong persistence but weaker targeting).

4) The paper presents ambitious scale-up trajectories. The missing step is to interpret what those imply for service delivery in South Africa in terms of:

clinic throughput

staff time requirements

stock management and continuity of injectable supply

the degree to which injectable PrEP competes for capacity with other high-priority services

This matters because the feasibility bottleneck for injectables is often not demand, but delivery capacity. If delivery constraints bind, realised coverage and persistence will fall below assumed levels, and the incremental advantage of LEN may narrow. Could the authors include a short operational feasibility discussion that links the projected scale-up volumes to plausible delivery platforms (ANC, PHC, differentiated services, KP services) and explicitly states which constraints are not represented structurally in the model? This does not require new modelling, but materially improves interpretability.

5) Comparisons between oral PrEP, CAB, and LEN are highly sensitive to assumptions about persistence and discontinuation, switching between products, visit schedules and behavioural response etc. Even when each modality is modelled reasonably, differences in these assumptions can unintentionally embed a programme advantage for one modality. Please add a brief statement explaining how you ensure comparability across modalities (i.e., that differences in outcomes reflect product and delivery characteristics rather than inconsistent modelling choices). If you already have a harmonised framework, make it explicit in the main text.

6) The model stratifies populations in a way that is appropriate for national planning, but PrEP cost-effectiveness and prioritisation can be highly sensitive to geographic heterogeneity (incidence hotspots, urban service access, mobility-linked risk) and to within-group risk gradients. A short discussion of how geographic targeting (or hotspot prioritisation) could alter the optimal early allocation of LEN, and whether the main conclusions are expected to be robust or could change under more granular targeting wuld be useful.

Summary recommendation

This is a strong submission with substantial policy relevance. The most important improvements to strengthen scientific robustness and decision credibility are:

1. a structural sensitivity analysis for imperfect targeting within groups,

2. a clearer formal definition of the constrained allocation objective and constraint, and

3. explicit treatment of persistence and delivery feasibility as the primary determinants of real-world impact.

Addressing these points would substantially increase confidence that the prioritisation conclusions will hold under realistic implementation conditions in South Africa.

Reviewer #2: Jamieson and colleagues examined the epidemiological impact and cost effectiveness of integrating lenacapavir into South Africa's HIV prevention response. This is a timely and important question: lenacapavir has demonstrated higher efficacy than oral PrEP, may help overcome the adherence challenges commonly seen with daily oral regimens, and is now available at a price of 40 USD. The authors used the well established Thembisa model—South Africa's primary HIV transmission model—to simulate the introduction of oral PrEP, cabotegravir, and twice yearly injectable lenacapavir (LEN) from 2026 to 2045. Their analysis focused on key populations at elevated HIV risk, including adolescent girls and young women, sex workers, pregnant and breastfeeding women, men who have sex with men, and heterosexual men.

The study found that LEN produced the greatest epidemiological impact. If 1.7-2.9 million people used LEN annually, it could avert roughly one fifth to one third of new HIV infections and preserve several percent of the life years otherwise expected to be lost to HIV. LEN scale up could also reduce national HIV incidence to below 0.1% more than a decade earlier than projected under current trends.

When LEN supply is limited, prioritising pregnant and breastfeeding women, sex workers, and men who have sex with men yields the largest reduction in infections. For broader national roll out, prioritising sex workers and MSM remains the most cost effective strategy.

Overall, providing LEN to individuals at highest HIV risk in South Africa appears substantially more cost effective than expanding existing PrEP options and could accelerate progress toward reducing HIV incidence.

The manuscript is well written and provides valuable insights. I do, however, have several comments.

Main comments

1.Assumptions favouring lenacapavir uptake

The model assumes that LEN uptake matches oral TDF/FTC in the conservative scenario and is twice as high in the optimistic scenario. These assumptions imply equal or greater preference for LEN over oral PrEP, which is not clearly supported by existing preference studies in other settings. Can the authors provide evidence that individuals at risk of HIV prefer LEN at these levels? Should a third scenario be included in which LEN uptake is lower than oral PrEP?

2.Longer duration of LEN use

LEN is modelled with the longest duration of use (6-12 months), compared with 4-8 months for cabotegravir and 3-6 months for oral PrEP. This inherently gives LEN a longer period of protection. I recommend adding an analysis in which all PrEP modalities are assigned the same duration of use to isolate the effect of the drug itself from the effect of duration.

3.Effectiveness during the pharmacokinetic tail

The authors appropriately include the pharmacokinetic tail of long acting PrEP. However, it is unclear whether the model incorporates the reduced effectiveness of cabotegravir during the tail phase, which has been documented in the literature (e.g., Landovitz et al., Lancet HIV 2020; Moore et al., IAS 2025). The tail phase for LEN remains unknown.

4.Length of the tail phase

Related to point 3, the model assumes a 3 month tail for cabotegravir, which appears to underestimate the duration reported in published studies. Conversely, the assumed 6 month tail for LEN may be an overestimate. Although LEN's tail is not yet characterised, available pharmacokinetic data (Jogiraju et al., Lancet 2025) suggest that six months may exceed the tail duration for some individuals.

Reviewer #3: This is a well conducted study on the epidemiological impact on HIV infections and life years lost, cost-effectiveness, and optimal populations for Lenacapavir roll-out in South Africa. The study design, statistical methods and analyses, and presentation (tables and figures) and interpretation of the results are mostly adequate and of a good standard. However, still a few issues needing attention.

1. Assumption and scenarios. Table 1 shows Key assumptions on coverage, duration and effectiveness 496 for main scenarios. However, there are too many assumptions and combinations which are difficult to follow. Usually, we have projected, worst and best scenarios, but here scenarios are all over the place so that the table becomes less informative and key messages are a bit lost. Is there any innovative way to summarise the key messages in the table?

2. Table 2 shows Impact and cost-effectiveness of oral TDF/FTC, injectable LEN or CAB scale-up compared with baseline over 2026-45. What are the models for projections here? deterministic or probabilistic?

3. Table 3 comparing 20-year impact (2026-2045) and cost-effectiveness of LEN and CAB scale-up scenarios to existing HIV interventions, with incremental cost effectiveness ratios compared to baseline. Again, what are the models used for ICER calculation? Have Markov models been used for ICER calculation and projection?

Reviewer #4: This is an interesting and relevant modeling study assessing the impact of scaling up LEN in the context of other HIV prevention interventions including CAB and oral PrEP. While previous studies have evaluated LEN scale up, to my knowledge, this is the first to assess it in the context of other strategies.

The authors focus the abstract and the discussion on the finding than LEN is more cost-effective than other HIV prevention interventions. I think the abstract should also highlight the important finding that LEN provision is less cost-effective than increasing ART, HIV testing, and MMC coverage. Indeed increasing ART coverage can save substantially more lives. And in the context of the likely funding disruptions, testing and treatment interventions will become even more cost-effective. This can also be emphasized more in the discussion, eg 8 times more life years are saved with ART expansion compared to LEN.

The authors mention than successful LEN scale up will depend on efficient delivery and demand generation. The PrEP delivery costs are robustly estimated using ingredients based costing. However, the authors estimate that the cost of LEN delivery is similar to that of oral PrEP (eg same staff time and salary costs). It is possible that LEN delivery costs may be higher than oral PrEP due to higher cadre personnel administering injections, consumables, and increased counseling/post injection observation time. This may increase LEN costs relative to oral PrEP (although likely outweighed by the less frequent visits). The authors may consider a sensitivity analysis to asses this.

Similarly, demand generation costs have been found to be substantial in oral PrEP programs. This is particularly important to achieve high uptake and continuation of LEN at the proposed levels and in the hard to reach populations. It would be helpful for the authors to conduct sensitivity analyses around the demand generation costs as it is not as robustly estimated as the provision costs.

In light of the newly estimated generic LEN cost of $25 ppy (plus loading dose) it might be helpful to run the CEA and BIA with the lower cost LEN product to highlight differences in efficiency and affordability.

(Optional) Due to the high potential of funding cuts/PEPFAR disruption, the authors should consider running a scenario comparing the HIV prevention interventions in the context of lower ART coverage and HIV testing. In this case, interventions to increase HIV testing and ART coverage may be more efficient than LEN or other prevention strategies as they immediately save lives.

In the limitation section, the authors should mention that the use of a compartmental model makes it harder to estimate the impact of partnership-level prevention strategies like PrEP. This could result in underestimation of LEN cost-effectiveness which may be why ICERs are higher than those of other network models (in addition to the higher provision costs assumed).

Any attachments provided with reviews can be seen via the following link: [LINK]

--------------------------------------------------------- ---

General editorial requests:

(Note: not all will apply to your paper, but please check each item carefully)

*We ask every co-author listed on the manuscript to fill in a contributing author statement, making sure to declare all competing interests. If any of the co-authors have not filled in the statement, we will remind them to do so when the paper is revised. If all statements are not completed in a timely fashion this could hold up the re-review process. If new competing interests are declared later in the revision process, this may also hold up the submission. Should there be a problem getting one of your co-authors to fill in a statement we will be in contact. Please do not add or remove authors without first discussing this with the handling editor. You can see our competing interests policy here: http://journals.plos.org/plosmedicine/s/competing-interests.

*Please upload any figures associated with your paper as individual TIF or EPS files with 300dpi resolution at resubmission; please read our figure guidelines for more information on our requirements: http://journals.plos.org/plosmedicine/s/figures. While revising your submission, we strongly recommend that you use PLOS's NAAS tool (https://ngplosjournals.pagemajik.ai/artanalysis) to test your figure files. NAAS can convert your figure files to the TIFF file type and meet basic requirements (such as print size, resolution), or provide you with a report on issues that do not meet our requirements and that NAAS cannot fix.

After uploading your figures to PLOS's NAAS tool - https://ngplosjournals.pagemajik.ai/artanalysis, NAAS will process the files provided and display the results in the "Uploaded Files" section of the page as the processing is complete.

If the uploaded figures meet our requirements (or NAAS is able to fix the files to meet our requirements), the figure will be marked as "fixed" above. If NAAS is unable to fix the files, a red "failed" label will appear above.

When NAAS has confirmed that the figure files meet our requirements, please download the file via the download option, and include these NAAS processed figure files when submitting your revised manuscript.

*Please ensure that the paper adheres to the PLOS Data Availability Policy (see http://journals.plos.org/plosmedicine/s/data-availability), which requires that all data underlying the study's findings be provided in a repository or as Supporting Information. For data residing with a third party, authors are required to provide instructions with contact information (web or email address) for obtaining the data. Please note that a study author cannot be the contact person for the data. PLOS journals do not allow statements supported by "data not shown" or "unpublished results." For such statements, authors must provide supporting data or cite public sources that include it.

*We expect all researchers with submissions to PLOS in which author-generated code underpins the findings in the manuscript to make all author-generated code available without restrictions upon publication of the work. In cases where code is central to the manuscript, we may require the code to be made available as a condition of publication. Authors are responsible for ensuring that the code is reusable and well documented. Please make any custom code available, either as part of your data deposition or as a supplementary file. Please add a sentence to your data availability statement regarding any code used in the study, e.g. "The code used in the analysis is available from Github [URL] and archived in Zenodo [DOI link]" Please review our guidelines at https://journals.plos.org/plosmedicine/s/materials-software-and-code-sharing and ensure that your code is shared in a way that follows best practice and facilitates reproducibility and reuse. Because Github depositions can be readily changed or deleted, we encourage you to make a permanent DOI'd copy (e.g. in Zenodo) and provide the URL.

*Please ensure that the study is reported according to the appropriate guideline and include the completed checklist as Supporting Information. When completing the checklist, please use section and paragraph numbers, rather than page numbers. Please add the following statement, or similar, to the Methods: "This study is reported as per [XXXX] guideline (S1 Checklist)."

FORMATTING - GENERAL

*Abstract: Please structure your abstract using the PLOS Medicine headings (Background, Methods and Findings, Conclusions). Please combine the Methods and Findings sections into one section. Please rename the Interpretation section.

*At this stage, we ask that you include a short, non-technical Author Summary of your research to make findings accessible to a wide audience that includes both scientists and non-scientists. The Author Summary should immediately follow the Abstract in your revised manuscript. This text is subject to editorial change and should be distinct from the scientific abstract. Ideally each sub-heading should contain 2-3 single sentence, concise bullet points containing the most salient points from your study. In the final bullet point of 'What Do These Findings Mean?', please include the main limitations of the study in non-technical language. Please see our author guidelines for more information: https://journals.plos.org/plosmedicine/s/revising-your-manuscript#loc-author-summary.

* Please include the funder URL in the study metadata.

*Please express the main results with 95% CIs as well as p values. When reporting p values please report as p<0.001 and where higher as the exact p value p=0.002, for example. Throughout, suggest reporting statistical information as follows to improve clarity for the reader "22% (95% CI [13%,28%]; p</=)". Please be sure to define all numerical values at first use.

*Please include page numbers and line numbers in the manuscript file. Use continuous line numbers (do not restart the numbering on each page).

*Please cite the reference numbers in square brackets. Citations should precede punctuation.

FIGURES AND TABLES

*Please provide titles and legends for all figures and tables (including those in Supporting Information files).

*Please define all abbreviations used in each figure/table (including those in Supporting Information files).

*Please consider avoiding the use of red and green in order to make your figure more accessible to those with color blindness.

SUPPLEMENTARY MATERIAL

*Please note that supplementary material will be posted as supplied by the authors. Therefore, please amend it according to the relevant comments outlined here.

*Please cite your Supporting Information as outlined here: https://journals.plos.org/plosmedicine/s/supporting-information

REFERENCES

*PLOS uses the numbered citation (citation-sequence) method and first six authors, et al.

*Please ensure that journal name abbreviations match those found in the National Center for Biotechnology Information (NCBI) databases (http://www.ncbi.nlm.nih.gov/nlmcatalog/journals), and are appropriately formatted and capitalised.

*Where website addresses are cited, please include the complete URL and specify the date of access (e.g. [accessed: 12/06/2023]).

*Please also see https://journals.plos.org/plosmedicine/s/submission-guidelines#loc-references for further details on reference formatting.

STUDY TYPE-SPECIFIC REQUESTS

MODELLING STUDIES

The following list is derived from Geoffrey P Garnett, Simon Cousens, Timothy B Hallett, Richard Steketee, Neff Walker. Mathematical models in the evaluation of health programmes. (2011) Lancet DOI:10.1016/S0140-6736(10)61505-X:

*If pertinent, please provide a diagram that shows the model structure, including how the natural history of the disease is represented, the process and determinants of disease acquisition, and how the putative intervention could affect the system.

*Please provide a complete list of model parameters, including clear and precise descriptions of the meaning of each parameter, together with the values or ranges for each, with justification or the primary source cited and important caveats about the use of these values noted.

*Please provide a clear statement about how the model was fitted to the data, including goodness-of-fit measure, the numerical algorithm used, which parameter varied, constraints imposed on parameter values, and starting conditions.

*For uncertainty analyses, please state the sources of uncertainties quantified and not quantified [can include parameter, data, and model structure].

*Please provide sensitivity analyses to identify which parameter values are most important in the model. Uncertainty estimates seek to derive a range of credible results on the basis of an exploration of the range of reasonable parameter values. The choice of method should be presented and justified.

*Please discuss the scientific rationale for the choice of model structure and identify points where this choice could influence conclusions drawn. Please also describe the strength of the scientific basis underlying the key model assumptions.

*For studies that develop a prediction model or evaluate its performance, please ensure that the study is reported according to the TRIPOD statement (https://www.equator-network.org/reporting-guidelines/tripod-statement) and include the completed checklist as Supporting Information. Please add the following statement, or similar, to the Methods: "This study is reported as per the Transparent Reporting of a Multivariable Prediction Model for Individual Prognosis Or Diagnosis (TRIPOD) statement (S1 Checklist)." For studies using machine learning, please use the TRIPOD-AI checklist. When completing the checklist, please use section and paragraph numbers, rather than page numbers.

HEALTH ECONOMICS / COST-EFFECTIVENESS STUDIES

*Please ensure that the study is reported according to the CHEERS guideline (available from: https://www.equator-network.org/reporting-guidelines/cheers) and include the completed checklist as Supporting Information. Please add the following statement, or similar, to the Methods: "This study is reported as per the Strengthening the Consolidated Health Economic Evaluation Reporting Standards 2022 (CHEERS 2022) Statement (S1 Checklist)." When completing the checklist, please use section and paragraph numbers, rather than page numbers.

# Author comment: Author response to Decision Letter 2

**DOI:** 10.1371/journal.pmed.1004882.r004

6 May 2026

Attachment Submitted filename: Responses to reviewers and editorial requests FINAL 05May26.pdf

# Peer review: Decision Letter 2

**DOI:** 10.1371/journal.pmed.1004882.r005

**Authors:** Alison Farrell

11 Jun 2026

Dear Dr. Jamieson,

Thank you very much for re-submitting your manuscript "Taking injectable PrEP to scale: An economic evaluation to optimise lenacapavir for South Africa’s HIV response " (PMEDICINE-D-25-04398R2) for review by PLOS Medicine.

I have discussed the paper with my colleagues and the academic editor and it was also seen again by four reviewers. I am pleased to say that provided the remaining editorial, reviewer and production issues are dealt with we are planning to accept the paper for publication in the journal.

The remaining issues that need to be addressed are listed at the end of this email. Any accompanying reviewer attachments can be seen via the link below. Please take these into account before resubmitting your manuscript:

[LINK]

***Please note while forming your response, if your article is accepted, you may have the opportunity to make the peer review history publicly available. The record will include editor decision letters (with reviews) and your responses to reviewer comments. If eligible, we will contact you to opt in or out.***

In revising the manuscript for further consideration here, please ensure you address the specific points made by each reviewer and the editors. In your rebuttal letter you should indicate your response to the reviewers' and editors' comments and the changes you have made in the manuscript. Please submit a clean version of the paper as the main article file. A version with changes marked must also be uploaded as a marked up manuscript file.

Please also check the guidelines for revised papers at http://journals.plos.org/plosmedicine/s/revising-your-manuscript for any that apply to your paper. If you haven't already, we ask that you provide a short, non-technical Author Summary of your research to make findings accessible to a wide audience that includes both scientists and non-scientists. The Author Summary should immediately follow the Abstract in your revised manuscript. This text is subject to editorial change and should be distinct from the scientific abstract.

In addition to these revisions, you may need to complete some formatting changes, which you will receive in a follow up email. A member of our team will be in touch with a set of requests shortly. If you do not receive a separate email within a few days, please assume that checks have been completed, and no additional changes are required.

We expect to receive your revised manuscript within 1 week. Please email us (plosmedicine@plos.org) if you have any questions or concerns.

We ask every co-author listed on the manuscript to fill in a contributing author statement. If any of the co-authors have not filled in the statement, we will remind them to do so when the paper is revised. If all statements are not completed in a timely fashion this could hold up the re-review process. Should there be a problem getting one of your co-authors to fill in a statement we will be in contact. YOU MUST NOT ADD OR REMOVE AUTHORS UNLESS YOU HAVE ALERTED THE EDITOR HANDLING THE MANUSCRIPT TO THE CHANGE AND THEY SPECIFICALLY HAVE AGREED TO IT.

Please ensure that the paper adheres to the PLOS Data Availability Policy (see http://journals.plos.org/plosmedicine/s/data-availability), which requires that all data underlying the study's findings be provided in a repository or as Supporting Information. For data residing with a third party, authors are required to provide instructions with contact information for obtaining the data. PLOS journals do not allow statements supported by "data not shown" or "unpublished results." For such statements, authors must provide supporting data or cite public sources that include it.

To enhance the reproducibility of your results, we recommend that you deposit your laboratory protocols in protocols.io, where a protocol can be assigned its own identifier (DOI) such that it can be cited independently in the future. Additionally, PLOS ONE offers an option to publish peer-reviewed clinical study protocols. Read more information on sharing protocols at https://plos.org/protocols?utm_medium=editorial-email&utm_source=authorletters&utm_campaign=protocols

Please review your reference list to ensure that it is complete and correct. If you have cited papers that have been retracted, please include the rationale for doing so in the manuscript text, or remove these references and replace them with relevant current references. Any changes to the reference list should be mentioned in the rebuttal letter that accompanies your revised manuscript.

Please note, when your manuscript is accepted, an uncorrected proof of your manuscript will be published online ahead of the final version, unless you've already opted out via the online submission form. If, for any reason, you do not want an earlier version of your manuscript published online or are unsure if you have already indicated as such, please let the journal staff know immediately at plosmedicine@plos.org.

If you have any questions in the meantime, please contact me or the journal staff on plosmedicine@plos.org.

We look forward to receiving the revised manuscript by Jun 18 2026 11:59PM.

Sincerely,

Alison Farrell, Ph.D.

Senior Editor

PLOS Medicine

plosmedicine.org

------------------------------------------------------------

Requests from Editors:

Abstract:

Please define all abbreviations at first use, including USD.

For USD in the main text, please define at first occurrence (i.e. not in the Methods).

Line 54: “under FSW scale-up” as meant? Or should this be “under scale-up to FSW”?

Author summary:

First bullet point: please mention HIV. Please also use ‘the Global Fund’, for consistency.

Line 73: add ‘or’ to read ‘or another injectable option’

GENERAL EDITORIAL REQUESTS

* Please revise your title so that it complies with PLOS Medicine's style. Your title must be nondeclarative and not a question. It should begin with main concept if possible. "Effect of" should be used only if causality can be inferred, i.e., for an RCT. Please place the study design ("A randomized controlled trial," "A retrospective study," "A modelling study," etc.) in the subtitle (ie, after a colon). For example: Scale-up of HIV pre-exposure prophylaxis in South Africa: An economic evaluation

* Please confirm that your abstract complies with our requirements, including format (three sections: Background, Methods and Findings, and Conclusions) and providing all the information relevant to this study type https://journals.plos.org/plosmedicine/s/submission-guidelines#loc-abstract

* Please ensure that the Introduction ends with a clear description of the study question or hypothesis.

* Please ensure that all abbreviations are defined at first use throughout the text.

* Please confirm that all numbers presented in the abstract are present and identical to numbers presented in the main manuscript text.

* Please convert any stacked bar charts to another data representation for example a table, or other type of graph.

* Please disclose any use of AI tools and technologies to the study or article content at the end of the Methods. Please specify the name(s) and version(s) of any tool(s) used and a description of their use, including how the AI-assisted or generated content was evaluated by authors. Please also include a statement specifying who retains responsibility for the final manuscript. Please see our guidelines for more information: https://journals.plos.org/plosmedicine/s/ethical-publishing-practice#loc-artificial-intelligence-tools-and-technologies.

FUNDING STATEMENT

* The funding statement should include: specific grant numbers, initials of authors who received each award, URLs to sponsors’ websites. Also, please state whether any sponsors or funders (other than the named authors) played any role in study design, data collection and analysis, the decision to publish, or preparation of the manuscript. If they had no role in the research, include this sentence: “The funders had no role in study design, data collection and analysis, decision to publish, or preparation of the manuscript.” Please include the funder URL(s).

DATA AVAILABILITY

* PLOS defines the “minimal data set” to consist of the data set used to reach the conclusions drawn in the manuscript with related metadata and methods, and any additional data required to replicate the reported study findings in their entirety. Authors do not need to submit their entire data set, or the raw data collected during an investigation. Please confirm that the manuscript includes the following data (note that all may not apply):

The values behind the means, standard deviations and other measures reported;

The values used to build graphs;

The points extracted from images for analysis.

* Please include the statement on code availability in the data availability statement. Please see guidance here:https://journals.plos.org/plosmedicine/s/code-sharing-guidance#loc-data-availability-statement-examples

We now require a digital identifier for code. Please add the DOI to the data availability statement.

FIGURES

* Please provide titles and legends for all figures and tables (including those in Supporting Information files). Please define all acronyms used in each figure or table in its corresponding legend.

ACKNOWLEDGMENTS

* Please include an Acknowledgments section in your manuscript.

ECONOMIC ANALYSES INCLUDING COST-EFFECTIVENESS ANALYSES

* Please report your economic analysis according to the appropriate study design provided at http://www.equator-network.org/?post_type=eq_guidelines&eq_guidelines_study_design=economic-evaluations&eq_guidelines_clinical_specialty=0&eq_guidelines_report_section=0&s= and provide the relevant completed checklist. In the checklist please include sufficient text excerpted from the manuscript to explain how you accomplished all applicable items.

MODELING STUDIES

For more details on the requests below please see Geoffrey P Garnett, Simon Cousens, Timothy B Hallett, Richard Steketee, Neff Walker. Mathematical models in the evaluation of health programmes. (2011) Lancet DOI:10.1016/S0140-6736(10)61505-X.

* Please provide a diagram that shows the model structure, including how the disease natural history is represented, the process and determinants of disease acquisition, and how the putative intervention could affect the system.

* Please provide a complete list of model parameters, including clear and precise descriptions of the meaning of each parameter, together with the values or ranges for each, with justification or the primary source cited, and important caveats about the use of these values noted.

* Please provide a clear statement about how the model was fitted to the data including where relevant goodness-of-fit measure, the numerical algorithm used, which parameter varied, constraints imposed on parameter values, and starting conditions.

* For uncertainty analyses, please state the sources of uncertainties quantified and not quantified this can include parameter, data, and model structure.

* Please provide sensitivity analyses to identify which parameter values are most important in the model. Uncertainty estimates seek to derive a range of credible results on the basis of an exploration of the range of reasonable parameter values. The choice of method should be presented and justified.

* Please discuss the scientific rationale for this choice of model structure and identify points where this choice could influence conclusions drawn. Please also describe the strength of the scientific basis underlying the key model assumptions.

Comments from Reviewers:

Reviewer #1: The responses to reviewers are all sufficient and I appreciate the time taken to do an additional sensitivity analysis to add credibility to this study.

Reviewer #2: Thank you for carefully addressing my comments.

Reviewer #3: Thanks authors for their great effort to improve the manuscript. The authors have addressed my comments well in a professional way. I am satisfied with the response and revision. No further issues needing attention.

Reviewer #4: The authors have addressed the majority of the reviewer comments which have strengthened the manuscript. A few minor comments remain:

In determining the optimal allocation authors state that they assumed all populations had an average duration on LEN for one year. This is an uncertain assumption can different persistence among different populations can impact targeted for optimal LEN allocation (eg if some subgroups have higher persistence than others). For example, pregnant women are more likely to discontinue oral PrEP after delivery, this pattern may also apply to LEN. Although the authors vary LEN persisistance from 6 months to 1 year, this is done uniformly. It would be helpful to note this in the limitation section as source of uncertainty.

The authors acknowledge their highly optimistic coverage assumptions, particularly among MSM and FSWs. However, the paper does not present sensitivity analyses varying coverage among these groups. The authors should consider evaluating this or providing advice to policymakers in the discussion section regarding optimal allocation of extra doses if target population coverage is not reached. For example, should doses that could not be delivered to MSM or FSWs be allocated to pregnant women or AGYW? What should happen with extra doses if coverage among these target populations is not realized or continuation is lower than expected?

Additionally, the authors state in the introduction: "Kaftan et al. estimated a range between $27-$106 PPPY depending on risk prioritisation, while Wu et al. estimated $177-$213 PPPY."

This should say: $32-$213 PPPY for the Wu at all paper as the lowest price per dose were found in Kenya ($16 per 6 monthly dose). The maximum prices varied widely by country and were highest for South Africa. It might be useful to mention in the discussion that this analysis may not be generalizable to other parts of Africa as South Africa is unique in its healthcare system and HIV epidemic. LEN may not be cost effective at current prices elsewhere in ESA.

Any attachments provided with reviews can be seen via the following link:

[LINK]

# Author comment: Author response to Decision Letter 3

**DOI:** 10.1371/journal.pmed.1004882.r006

26 Jun 2026

Attachment Submitted filename: Responses to reviewers and editorial requests 23.06.2026.docx

# Editor report: Decision Letter 3

**DOI:** 10.1371/journal.pmed.1004882.r007

**Authors:** Alison Farrell

26 Jun 2026

Dear Dr Jamieson,

On behalf of my colleagues and the Academic Editor, Simon Cauchemez, I am pleased to inform you that we have agreed to publish your manuscript "Taking injectable pre-exposure prophylaxis to scale: A modelling study and economic evaluation to optimise lenacapavir for South Africa’s HIV response" (PMEDICINE-D-25-04398R3) in PLOS Medicine.

Before your manuscript can be formally accepted you will need to complete some formatting changes, which you will receive in a follow up email. Please be aware that it may take several days for you to receive this email; during this time no action is required by you. Once you have received these formatting requests, please note that your manuscript will not be scheduled for publication until you have made the required changes.

Please also note that we need a further revision to your title to adhere to PLOS Medicine guidelines, which require that only the study type follow the colon. Please therefore consider the following options:

'Optimising scale-up of injectable lenacapavir for HIV pre-exposure prophylaxis in South Africa: A modelling study and economic evaluation"

or "Scale-up of injectable lenacapavir for pre-exposure prophylaxis to optimise South Africa's HIV response: A modelling study and economic evaluation"

Or provide an alternative, with only "A modelling study and economic evaluation" after the colon.

Please also note the following requested text revisions:

Line 318: please indicate 'LEN saved 593,000-...' if correct (unclear at present)

Line 415: add women after 'pregnant'

Line 432: make programme plural (programmes attain) or reword (the programme attains)

Line 466: revise "costs of implementing of long-acting" to remove the second 'of'

In the meantime, please log into Editorial Manager at http://www.editorialmanager.com/pmedicine/, click the "Update My Information" link at the top of the page, and update your user information to ensure an efficient production process.

PRESS

We frequently collaborate with press offices. If your institution or institutions have a press office, please notify them about your upcoming paper at this point, to enable them to help maximise its impact. If the press office is planning to promote your findings, we would be grateful if they could coordinate with medicinepress@plos.org. If you have not yet opted out of the early version process, we ask that you notify us immediately of any press plans so that we may do so on your behalf.

We also ask that you take this opportunity to read our Embargo Policy regarding the discussion, promotion and media coverage of work that is yet to be published by PLOS. As your manuscript is not yet published, it is bound by the conditions of our Embargo Policy. Please be aware that this policy is in place both to ensure that any press coverage of your article is fully substantiated and to provide a direct link between such coverage and the published work. For full details of our Embargo Policy, please visit http://www.plos.org/about/media-inquiries/embargo-policy/.

To enhance the reproducibility of your results, we recommend that you deposit your laboratory protocols in protocols.io, where a protocol can be assigned its own identifier (DOI) such that it can be cited independently in the future. Additionally, PLOS ONE offers an option to publish peer-reviewed clinical study protocols. Read more information on sharing protocols at https://plos.org/protocols?utm_medium=editorial-email&utm_source=authorletters&utm_campaign=protocols

Thank you again for submitting to PLOS Medicine. We look forward to publishing your paper.

Sincerely,

Alison Farrell, Ph.D.

Senior Editor

PLOS Medicine
